# Photoswitchable interlocked thiodiglycolamide as a cocatalyst of a chalcogeno-Baylis–Hillman reaction†
†Electronic supplementary information (ESI) available: Experimental procedures, spectroscopic and mass spectrometry data for all of the new compounds, and the full crystallographic details of **3a**. CCDC 1532346. For ESI and crystallographic data in CIF or other electronic format see DOI: 10.1039/c7sc00724h



**DOI:** 10.1039/c7sc00724h

**Published:** 2017-03-07

**Authors:** Alberto Martinez-Cuezva, Adrian Saura-Sanmartin, Tomas Nicolas-Garcia, Cristian Navarro, Raul-Angel Orenes, Mateo Alajarin, Jose Berna

**Affiliations:** a Departamento de Química Orgánica , Facultad de Química , Regional Campus of International Excellence “Campus Mare Nostrum” , Universidad de Murcia , E-30100 , Murcia , Spain . Email: ppberna@um.es; b SAI , Universidad de Murcia , E-30100 , Murcia , Spain

## Abstract

En route to a photoswitchable interlocked catalyst we have proved the ability of thiodiglycolamide to act as a template in the formation of hydrogen-bonded [2]rotaxanes. X-ray diffraction studies reveal the shielding of the sulfide atom by the macrocycle. A series of molecular shuttles are described as having an isomerizable fumaramide and thiodiglycolamide binding sites for controlling the relative ring position at will. By employing these systems as photoregulated catalysts, the TiCl_4_-mediated chalcogeno-Morita–Baylis–Hillman reaction is tested. In the presence of the maleamide shuttle, in which the sulfide function is encapsulated by the macrocycle, a complete loss in control of the geometry of the produced aldol is observed. The *E*-aldol adduct is predominantly obtained when the photoisomerized fumaramide shuttle, in which the sulfide function is exposed, is used.

## Abstract 2

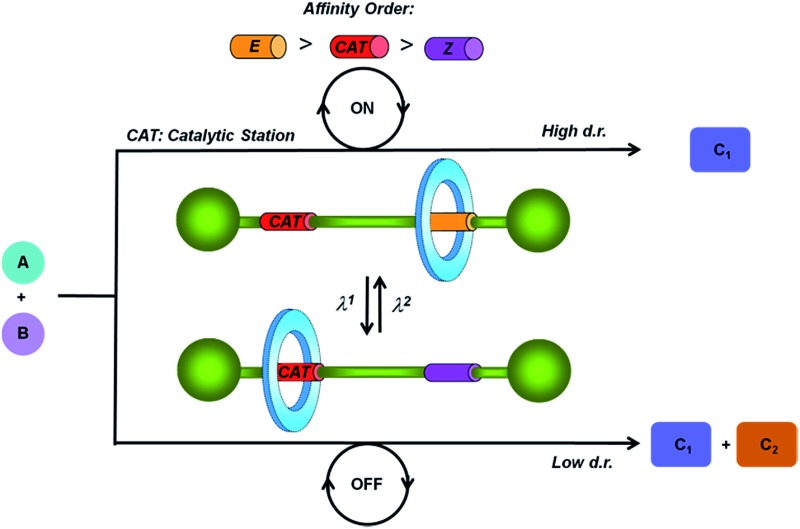
The sulfur-based template of light-driven molecular shuttles is able to modulate its catalytic activity in a diastereoselective chalcogeno-Baylis–Hillman reaction.

## Introduction

Taking as model the majority of the enzymatically driven processes that are ubiquitous in nature,^1^ chemists are devoting much effort to the designing of catalysts with switchable activity.^2^ These compounds are able to control programmed multistep procedures or selectively afford synthetic targets. Among the external triggering stimuli, both light^3^ and chemical reactions,^4^ including coordination events,^4c–e^ redox reactions,^4*f*^ and changes in pH,^4a,b^ solvent^4*g*^ or temperature,^4*h*^ have been employed to control the catalytic activity and the regio- and stereoselectivity of the reactions in which they are involved.^2^

Although the first examples of the incorporation of catalytic centers in rotaxanes^5^ were reported in the last decade,^6^ the use of interlocked molecular architectures^7^ as switchable catalysts is a growing topic nowadays.^2,8^ In this area, pH-driven molecular shuttles have been used for controlling the catalytic activity or the result of a particular reaction. Some recent examples of these systems include the control of the rate^9^ and the stereochemistry^10^ of Michael addition reactions or participation in selected chemical transformations through different activation modes.^11^ To the best of our knowledge, light-driven threaded systems^12^ programmed to modify their catalytic activity at will remain unexplored. In order to tackle this issue, herein we have designed a photo-responsive molecular shuttle that could communicate the reactivity of a nucleophilic center in one of its states, whereas the same feature remained silenced in the other state.

**Fig. 1:**
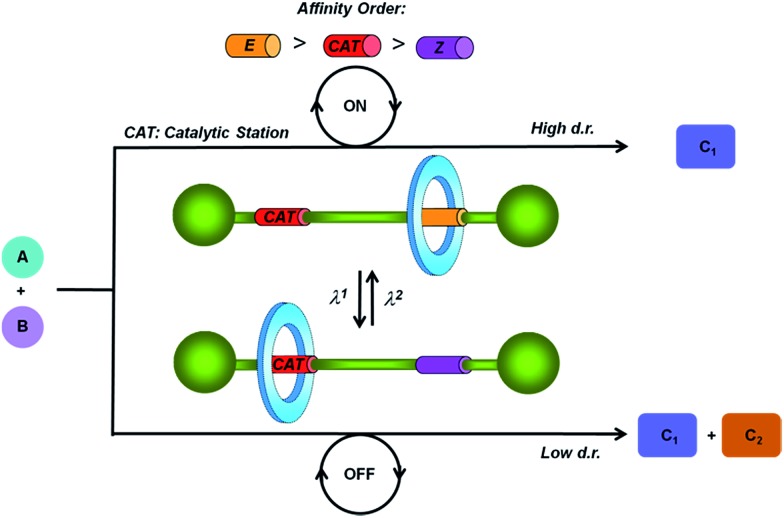
Representation of a photoswitchable *E*/*Z* interlocked catalyst.

In this regard we have envisioned a photoswitchable interlocked catalyst by the incorporation of a sulfide functionality^13^ in one of the two binding sites of a hydrogen-bonded molecular shuttle^5,7,8a,14^ that also contains a photoisomerizable fumaramide station (Fig. 1).^15^ We expected that in the active mode of this catalyst, in which the sulfide is exposed to the surrounding reaction medium, the system catalyzes an organic transformation in a selective fashion (catalysis ON). This chemical behavior would drastically change by the light-promoted *E* to *Z* interconversion of the olefinic station promoting the coverage of the sulfur-based station by the macrocycle, and thus precluding its participation in the considered process (catalysis OFF).

For the catalytic model reaction we selected one of the most appealing organic transformations that is catalyzed by sulfides,^16^ the Morita–Baylis–Hillman (MBH) reaction. This atom-economic carbon–carbon bond forming reaction is broadly used for the functionalization of activated alkenes or alkynes by reaction with different electrophiles under the influence of a catalytic system.^17^

## Results and discussion

### Hydrogen-bonding directed assembly of thiodiglycolamide [2]rotaxanes

The formation of amide-based rotaxanes requires the use of a suitable template to maximize the efficiency of the assembly.^18^ In the case of tetralactam-based rotaxanes having four benzylic amide functions in the ring, a variety of structural motifs have been employed, including 1,4-dicarboxamides,^19^ nitrones,^20^ squaraines,^21^ organophosphorus species,^22^ di(acylamino)pyrididines^23^ and azodicarboxamides.^6d,24^

**Scheme 1:**
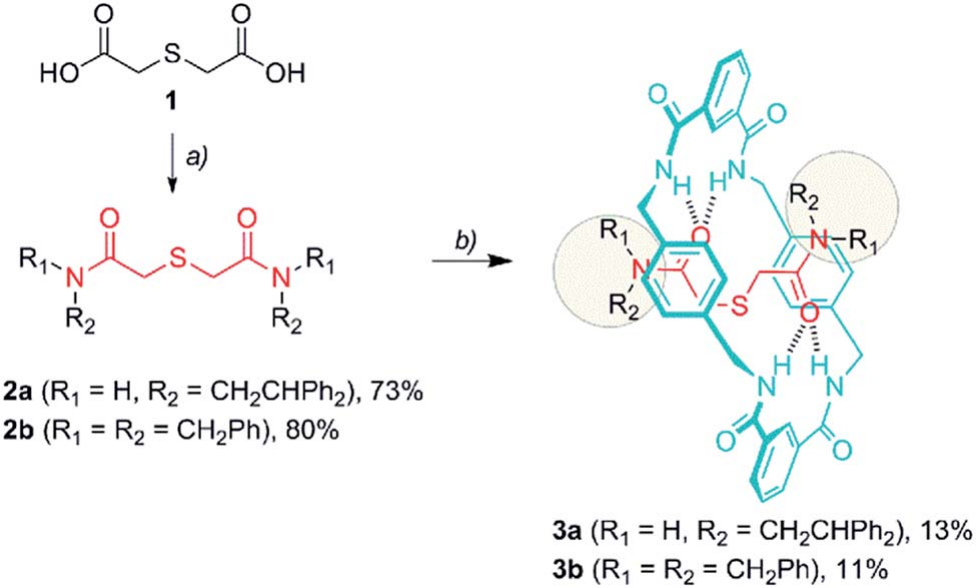
Synthesis of thiodiglycolamides **2** and the [2]rotaxanes **3**. Reagents and conditions: (a) (i) (COCl)_2_, DMF (cat) and CH_2_Cl_2_; (ii) 2,2-diphenylethylamine or dibenzylamine, Et_3_N, CHCl_3_, **2a** (73%) and **2b** (80%), and (b) isophthaloyl dichloride, *p*-xylylenediamine, Et_3_N, CHCl_3_, **3a** (13%), and **3b** (11%).

Thiodiglycolamides **2**, that are easily obtained from commercially available thiodiglycolic acid **1** (Scheme 1), were chosen as the sulfur-containing threads. These compounds contain two carbonyl groups as hydrogen bond acceptors, two bulky groups as stoppers and an embedded sulfur atom as the future catalytic active center. Firstly, the ability of these compounds to template the assembly of benzylic amide [2]rotaxanes was assayed. The five-component clipping reaction of **2a** using *p*-xylylenediamine and isophthaloyl chloride in the presence of triethylamine afforded the [2]rotaxane **3a**. The surrogate **3b** was also prepared, which bears two dibenzylamino groups as the stoppers of the interlocked species, enhancing its solubility in chlorinated solvents when compared to **3a**. As we expected, both of the rotaxanes were obtained in low yields (**3a**, 13%; **3b**, 11%) indicating a moderate affinity of the thiodiglycolamide binding site for the macrocycle.

### Molecular structure of the hydrogen-bonded [2]rotaxane **3a** in the solid state

**Fig. 2:**
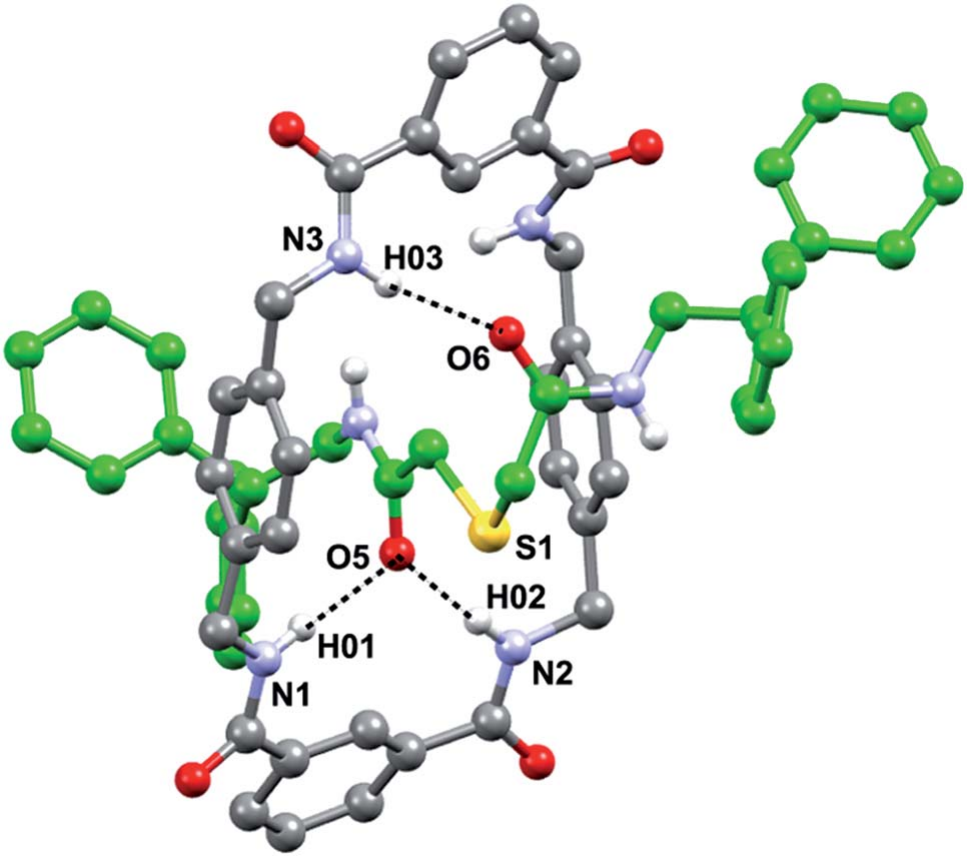
X-ray structure of the thiodiglycolamide [2]rotaxane **3a** crystallized from CH_3_CN. For clarity, the carbon atoms of the macrocycle are shown in grey, the carbon atoms of the thread are shown in green, the oxygen atoms are depicted in red, the nitrogen atoms are depicted in blue, and selected hydrogen atoms are shown in white. Also for clarity, the solvent molecules have been omitted. Intramolecular hydrogen-bond lengths [Å] (and angles [deg]): O5HN1 2.204 (176.8), O5HN2 2.096 (177.5) and O6HN3 2.096 (152.6).

In order to get a detailed view of the chemical environment of the active center once encapsulated by the macrocycle, we grew suitable monocrystals for X-ray diffraction analysis by slowly evaporating a solution of **3a** in acetonitrile. The resulting interlocked molecular structure of **3a** (Fig. 2) shows a bifurcated hydrogen bond (HB) between two of the NH groups of the macrocycle with one of the CO groups of the thiodiglycolamide thread, and a single HB between another NH group of the macrocycle with the second CO group of the thread. The CH_2_SCH_2_ skeleton between the HB acceptor of the thread adopts a folded conformation that is established by CH···π interactions between one of its methylenic hydrogen atoms and one of the *p*-xylylene groups of the tetralactam ring (3.4 Å, 86°) (Fig. S3, see ESI†). In contrast with other sulfur-containing functionalities used as templates in the building of amide based rotaxanes,^13*c*^ the sulfur atom incorporated in the thiodiglycolamide **2a** seems to be a mere spectator during the rotaxane formation.

An inspection of the structure of the interlocked thiodiglycolamide **3a** in the solid state reveals a reasonable shielding of the sulfur functionality which could reduce or even preclude its participation in a chemical reaction. The next step in our research was directed towards the incorporation of this binding site station into a photoswitchable molecular shuttle.

### Synthesis of photoswitchable thiodiglycolamide molecular shuttles

**Scheme 2:**
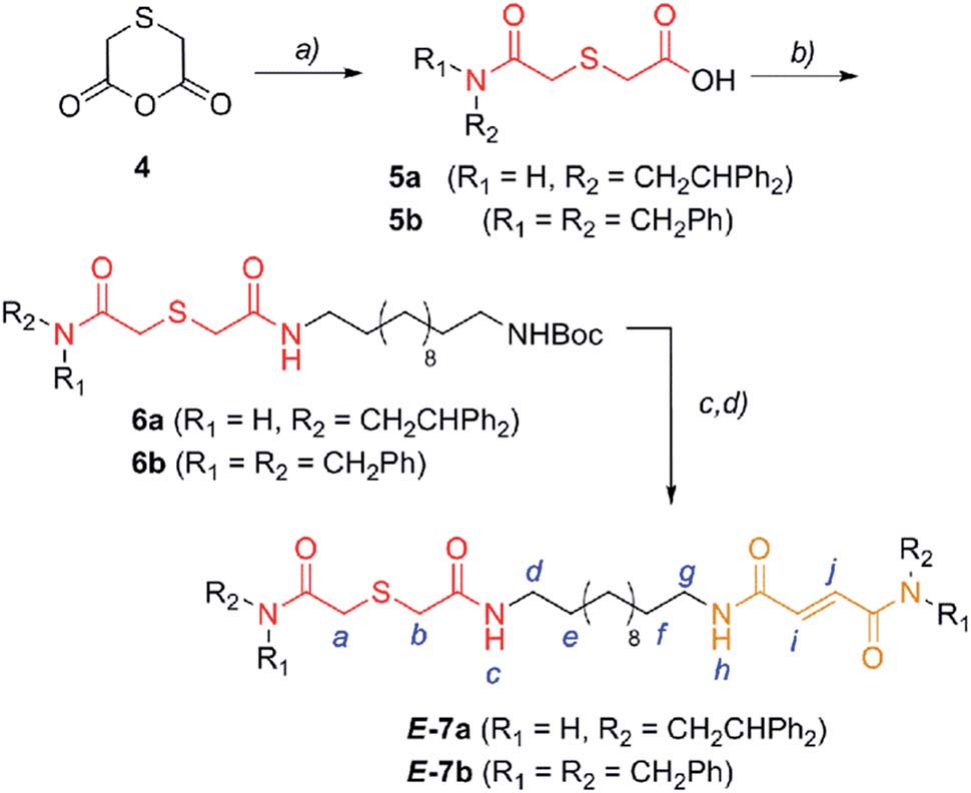
Synthesis of two binding site threads ***E*-7a,b**. Reagents and conditions: (a) 2,2-diphenylethylamine or dibenzylamine, pyridine, reflux, Et_2_O, **5a** (70%) and **5b** (48%); (b) 12-(*tert*-butoxycarbonylamino)dodecylamine (**S1**), EDCI, DMAP, CH_2_Cl_2_, **6a** (43%) and **6b** (94%); (c) CF_3_CO_2_H and CH_2_Cl_2_; and (d) (*E*)-4-(2,2-diphenylethylamino)-4-oxobut-2-enoic acid (**S2a**) or (*E*)-4-(dibenzylamino)-4-oxobut-2-enoic acid (**S2b**), EDCI, DMAP, CH_2_Cl_2_, **7a** (59%) and **7b** (66%). The full experimental procedure can be found in the ESI.†

En route to the targeted interlocked catalysts, the first synthetic step required the nucleophilic opening of anhydride **4**,^25^ which was easily obtained from thiodiglycolic acid (**1**), with 2,2-diphenylethylamine or dibenzylamine to provide the carboxylic acids **5** (Scheme 2). Next, the amide formation reaction of **5** with a monoBoc protected dodecyldiamine (**S1**) was carried out to produce the carbamates **6**. After amine deprotection, a second amide formation reaction with a fumaric acid semiamide^12f,26^ (**S2**) allowed for the incorporation of the olefinic station into the dumbbell-shaped compounds ***E*-7** (Scheme 2).

**Scheme 3:**
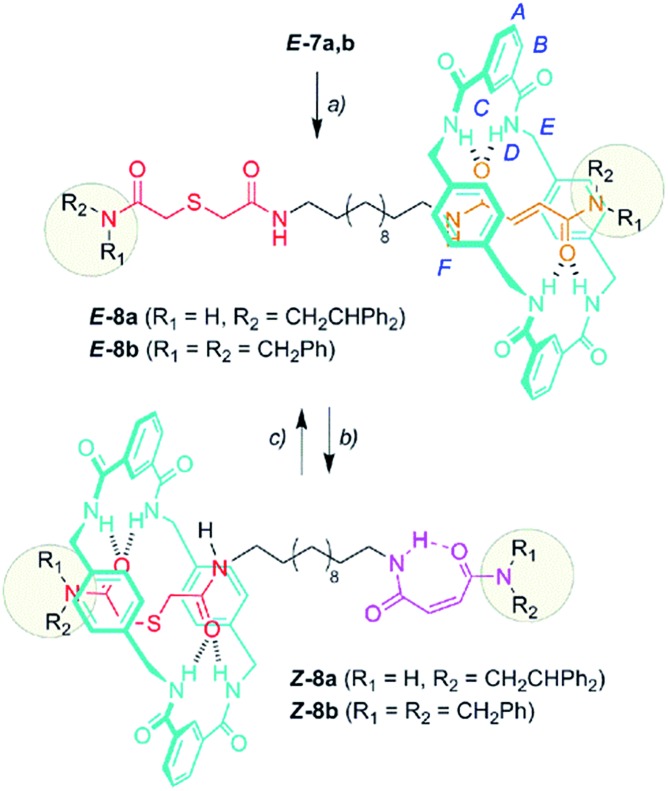
Synthesis of molecular shuttles **8** and the interconversion of their translational co-conformers. Reagents and conditions: (a) isophthaloyl dichloride, *p*-xylylenediamine, Et_3_N, CHCl_3_, ***E*-8a** (25%) and ***E*-8b** (46%); (b) 254 nm, CH_2_Cl_2_, ***Z*-8a** (49%) and ***Z*-8b** (53%); and (c) 312 nm, CH_2_Cl_2_, ***E*-8a** (46%) and ***E*-8b** (58%). The full experimental procedure can be found in the ESI.†

With the sulfur containing threads ***E*-7** in hand, we assembled the benzylic amide [2]rotaxanes ***E*-8** *via* a five component clipping reaction using *p*-xylylenediamine, isophthaloyl dichloride and triethylamine in chloroform (Scheme 3). The photoisomerization of the *trans* double bond of the interlocked compounds ***E*-8** led to the corresponding maleamide counterparts ***Z*-8**. The recovery of the starting shuttle was efficiently reached *via cis*-to-*trans* isomerization promoted by irradiating at 312 nm (conditions c, Scheme 3).

**Fig. 3:**
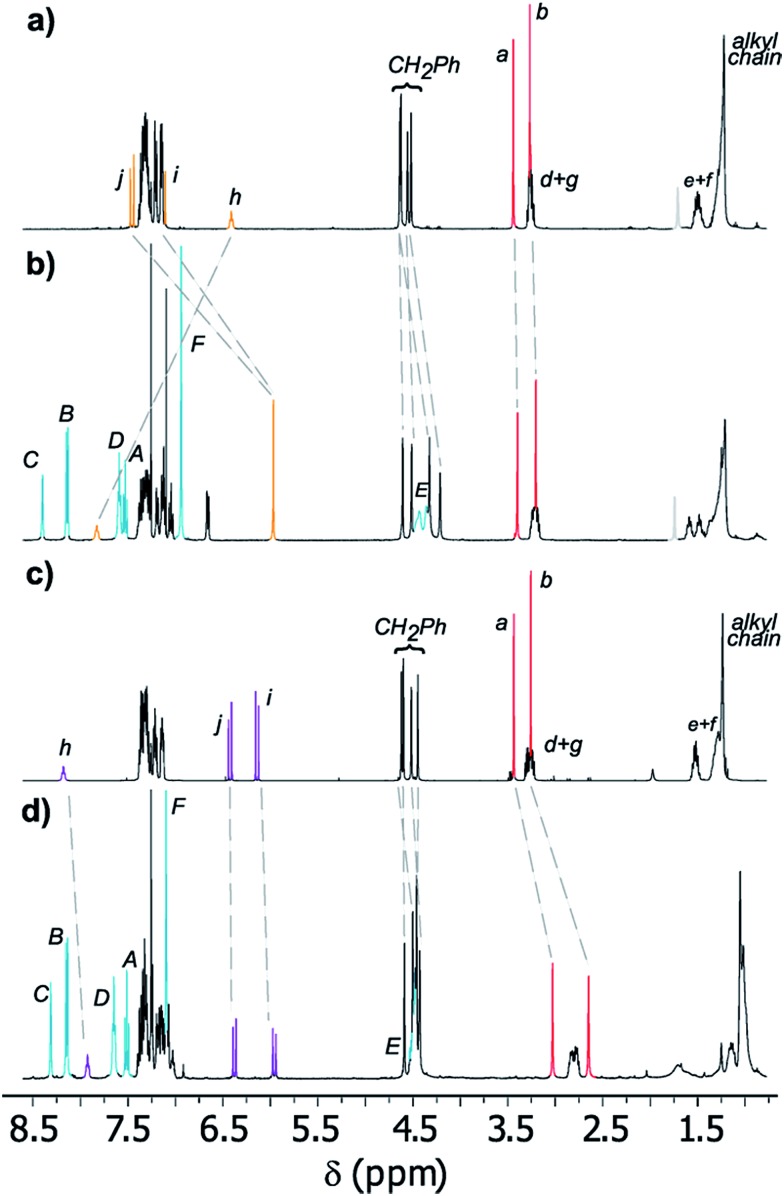
Partial ^1^H NMR spectra (400 MHz, CDCl_3_, 298 K) of (a) thread ***E*-7b**, (b) [2]rotaxane ***E*-8b**, (c) thread ***Z*-7b**, and (d) [2]rotaxane ***Z*-8b**. The assignments correspond to the lettering shown in Schemes 2 and 3.

Fig. 3 displays the stacked ^1^H NMR spectra of threads **7b** ^27^ and rotaxanes **8b**. The comparison of these spectra allows us to establish the relative location of the ring in the interlocked systems. The thiodiglycolamide signals (H_a_ and H_b_, red) are at similar chemical shifts in both the thread and rotaxane (see the traces in Fig. 3a and b), thus proving that this station is empty in ***E*-8b**, whereas the hydrogens of the methyne fumaramide (H_i_ and H_j_, orange) are shifted ∼1.33 ppm upfield in this rotaxane with respect to its naked thread ***E*-7b** due to the shielding effect of the xylylene aromatic rings. So, in this co-conformer the macrocycle is mainly located at the fumaramide station of ***E*-8b**.

Additionally, while the maleamide signals of ***Z*-8b** appear at similar chemical shifts to those of thread ***Z*-7b**, the thiodiglycolamide signals of ***Z*-8b** are shifted by 0.40 ppm with respect to those of ***Z*-7b** (see the traces in Fig. 3c and d), suggesting that the ring moves over to this binding site and the light-driven ring translocation from the fumaramide unit to the sulfide moiety occurs with excellent positional integrity.

### Carbon–carbon bond forming reactions using thiodiglycolamide [2]rotaxanes as catalysts

Having obtained our photoswitchable sulfide-based [2]rotaxanes, we focused on the chalcogeno-Baylis–Hillman reaction developed by Kataoka *et al.* ^28^ The presence of a catalytic amount of a chalcogenide (a sulfur or selenide derivative) enabled the control of the stereochemistry (*cis*–*trans* configuration) of the chloromethylene aldols obtained *via* TiCl_4_ promoted reactions between aldehydes and activated alkynes. Thus the presence of the catalytic amounts of dimethyl sulfide predominantly led to the formation of the *E*-isomer at short reaction times and low temperatures. In order to explore the viability of the thiodiglycolamide-based [2]rotaxanes for acting as catalysts of this transformation we tested the TiCl_4_-promoted Baylis–Hillman reaction between *p*-nitrobenzaldehyde and 3-butyn-2-one in the presence of catalytic amounts of our interlocked and non-interlocked thiodiglycolamides. First, we carried out this reaction in the presence of sulfide **2a** (0.1 equiv.; Table 1, entry 1) and TiCl_4_ (1 equiv.) at 5 °C for 3 h affording a mixture of the geometrical isomers of α-chloromethylene aldol **9** with the olefinic *E* product as the major isomer (85 : 15 dr). The better solubility of **2b** enhances this ratio up to 91 : 9 (Table 1, entry 2). If the single-binding site rotaxanes **3** are used as the sulfide sources, the stereochemical control is null and a practically equimolar mixture of *E* and *Z* aldols is obtained (Table 1, entries 3 and 4) as a consequence of the shielding of the sulfide fragment by the macrocycle, which precludes its participation in the reaction. The use of the interlocked catalyst ***E*-8a** affords aldol **9** in a 56 : 44 ratio in favor of the *E* isomer (Table 1, entry 5). When the most soluble derivative ***E*-8b** is employed, better control is obtained (80 : 20) as a result of an increase in the interlocked sulfide concentration in the reaction (Table 1, entry 6) (see Fig. S1†). Finally, a 1 : 1 mixture of both isomers of **9** are obtained in the presence of the interlocked catalyst ***Z*-8b** in which the ring is positioned over the thiodiglycolamide station (Table 1, entry 7). As expected, the diastereomeric ratios achieved in the presence of **3a**, **3b** and ***Z*-8b** are similar to the one obtained in the absence of the chalcogenide derivative (Table 1, entry 8). Notably, the respective sulfide-based catalyst remains unaltered at the end of these experiments, and it could be recovered for further use.

**Table 1:** Thiodiglycolamide-catalyzed Baylis–Hillman reaction mediated by TiCl_4_ ^*a*^

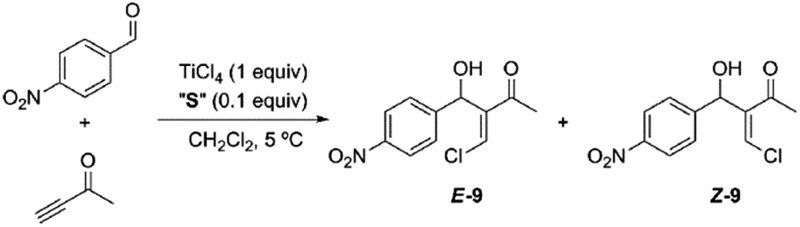			
Entry	Sulfide “**S**”	% conversion^*b*^	d.r. (*E* : *Z*)^*c*^
1	**2a**	94	85 : 15
2	**2b**	92	91 : 9
3	**3a**	90	55 : 45
4	**3b**	85	50 : 50
5	***E*-8a**	70	56 : 44
6	***E*-8b**	71	80 : 20
7	***Z*-8b**	54	50 : 50
8	—	92	50 : 50

^*a*^Reaction conditions: a mixture of *p*-nitrobenzaldehyde (0.10 mmol), 3-butyn-2-one (0.30 mmol) and the corresponding sulfide “**S**” (0.01 mmol) in dry CH_2_Cl_2_ under an N_2_ atmosphere was stirred at 5 °C. TiCl_4_ (0.10 mmol) was added and the mixture was stirred for 3 h at the indicated temperature.

^*b*^The conversions were determined using ^1^H NMR analysis.

^*c*^The diastereomeric ratio was determined using ^1^H NMR analysis.

Although the reaction conversion using the two station [2]rotaxane decreased with respect to those obtained using non-interlocked catalysts, the employment of the optimized *trans* co-conformer ***E*-8b** allowed a maximum 80 : 20 ratio to be achieved in favor of the chloromethylene aldol ***E*-9**. The photoisomerized *cis* co-conformer ***Z*-8** keeps the sulfide function in a latent state and is kinetically protected by the surrounding ring against other external reagents.^8*d*^

This example shows for the first time that the employment of photoswitchable interlocked catalysts can be useful to control the stereochemical course of a reaction. In contrast to pH-driven catalysts, this unprecedented light-responsive interlocked catalyst is enabled to work in neutral media, expanding its potential applicability to a broad variety of transformations in which these molecular shuttles can play an important role in regulating the outcome of the reactions at will.

## Conclusions

In summary, we have shown that thiodiglycolamides are able to act as a template in the formation of benzylic amide rotaxanes. The molecular structure of one of these interlocked compounds in the solid state reveals that its sulfide functional group is well covered by the macrocyclic tetralactam and so is isolated from its surroundings. These findings were used to design a switchable interlocked sulfide which includes a fumaramide binding site as a photoisomerizable unit for controlling the relative ring position. Two versions of this prototype varying the number of secondary amides in the thread (and then differing in solubility in halogenated solvents) were straightforwardly synthesized from thiodiglycolic anhydride. The behavior of the sulfide function of both translational co-conformers of these molecular shuttles was assayed in a MBH type reaction promoted by TiCl_4_ using *p*-nitrobenzaldehyde and 3-butyn-2-one. With the fumaramide shuttle, in which the sulfide function is exposed, the *E*-aldol adduct is predominantly obtained. Using the photoisomerized shuttle, in which the sulfide function is encapsulated by the macrocycle, a complete loss of diastereoselectivity is observed.
