# Supplementary material for: Photoswitchable interlocked thiodiglycolamide as a cocatalyst of a chalcogeno-Baylis–Hillman reaction
Source: Chem Sci. 2017 Mar 7;8(5):3775–80. doi: 10.1039/c7sc00724h (PMC5436546; doi:10.1039/c7sc00724h)

Electronic Supplementary Material (ESI) for Chemical Science.
This journal is © The Royal Society of Chemistry 2017

Electronic Supplementary Information

Photoswitchable Interlocked Thiodiglycolamide as Cocatalyst of a Chalcogeno-Baylis-Hillman Reaction

*Alberto Martinez-Cuezva,[a] Adrian Saura-Sanmartin,[a] Tomas Nicolas-Garcia,[a] Cristian Navarro,[a] Raul-Angel Orenes,[b] Mateo Alajarin,[a] and Jose Berna[a,]**

a) Departamento de Química Orgánica, Facultad de Química, Regional Campus of International Excellence "Campus Mare Nostrum", Universidad de Murcia, E-30100 Murcia (Spain).

b) Sección Universitaria de Instrumentación Científica (SUIC), Servicio de Apoyo a la Investigación (SAI), Regional Campus of International Excellence "Campus Mare Nostrum", Universidad de Murcia, E-30100 Murcia (Spain).

Email: ppberna@um.es

Table of Contents………………………………………………………………………………………. S2

1. General experimental section………………………………………………………………………. S3

2. Synthesis of threads 2 ……………………………………………………………………………… S4

3. Synthesis of the threads *E*-7 ……………………………………………………………………… S5

4. Synthesis of the threads *Z*-7 ……………………………………………………………………… S10

5. General procedure for the preparation of the [2]rotaxanes 3 and *E*-8 ..…………………………… S12

6. Photoisomerization of the molecular shuttles 8 ……………………………………………………… S15

7. Chalcogenide-$TiCl_4$ catalyzed Baylis-Hillman reaction …………………………………………….. S17

8. Crystal data and structure refinement for rotaxane 3a …………………………………………….. S19

9. ^{1}H and ^{13}C NMR spectra of synthesized compounds ………………………………………………… S22

1. General Experimental Section

Unless stated otherwise, all reagents were purchased from Aldrich Chemicals and used without further purification. HPLC grade solvents (Scharlab) were nitrogen saturated and were dried and deoxygenated using an Innovative Technology Inc. Pure-Solv 400 Solvent Purification System. Column chromatography was carried out using silica gel (60 Å, 70-200 μm, SDS) as stationary phase, and TLC was performed on precoated silica gel on aluminun cards (0.25 mm thick, with fluorescent indicator 254 nm, Fluka) and observed under UV light. All melting points were determined on a Kofler hot-plate melting point apparatus and are uncorrected. ^{1}H- and ^{13}C-NMR spectra were recorded at 298 K on a Bruker Avance 300 and 400 MHz instruments. ^{1}H NMR chemical shifts are reported relative to Me_4Si and were referenced via residual proton resonances of the corresponding deuterated solvent whereas ^{13}C NMR spectra are reported relative to Me_4Si using the carbon signals of the deuterated solvent. Signals in the ^{1}H and ^{13}C NMR spectra of the synthesized compounds were assigned with the aid of DEPT, APT, or two-dimensional NMR experiments (COSY, HMQC and HMBC). Abbreviations of coupling patterns are as follows: br, broad; s, singlet; d, doublet; t, triplet; q, quadruplet; m, multiplet. Coupling constants *(J)* are expressed in Hz. High-resolution mass spectra (HRMS) were obtained using a time-of-flight (TOF) instrument equipped with electrospray ionization (ESI)..

Abbreviation list:
THF: tetrahydrofuran
DMSO: dimethylsulfoxide
DMAP: dimethylaminopyridine
EDCI: *N*-(3-Dimethylaminopropyl)-**N**′-ethylcarbodiimide hydrochloride
DMF: *N,N*-dimethylformamide
BOP: (Benzotriazol-1-yloxy)tris(dimethylamino)phosphonium hexafluorophosphate
TFA: trifluoroacetic acid

2. Synthesis of threads 2

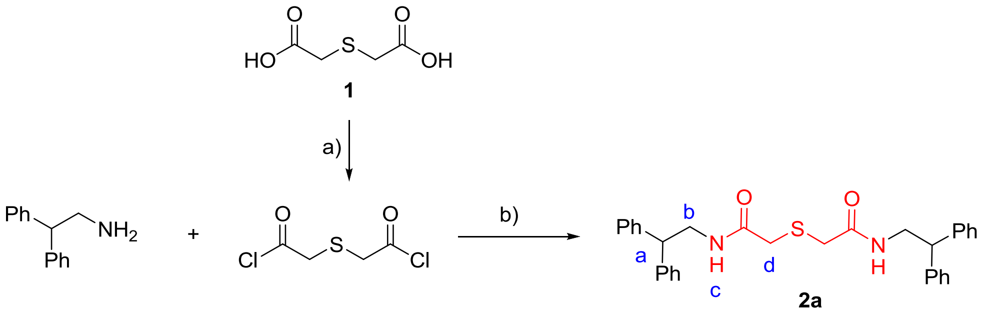

Scheme S1. Synthesis of thread **2a**: a) oxalyl chloride, DMF (cat.), CH_2Cl_2, reflux, 3 h; b) Et_3N, $CHCl_3$, r.t. to reflux, 3 h.

To a suspension of 2,2'-thiodiacetic acid **1** (1.5 g, 10.0 mmol) in dry dichloromethane (40 mL) under N_2 atmosphere was added oxalyl chloride (3.81 g, 30.0 mmol) and DMF (4 drops). The mixture was stirred for 3 hours under reflux. After this time the solvent and the excess of oxalyl chloride were removed under reduced pressure. The resulting residue was used in the next step without purification.

To a solution of 2,2-diphenylethanamine (2.30 g, 11.7 mmol) and Et_3N (2.25 mL, 16.0 mmol) in $CHCl_3$ (60 mL) was added dropwise the residue obtained in the previous step (1.00 g, 5.35 mmol), dissolved in 10 mL of $CHCl_3$, during a period of 1 hour. After stirring at room temperature for another hour, the reaction was refluxed for 3 hours. The reaction mixture was washed with water (2 x 100 mL), HCl 1N (2 x 100 mL), NaOH 1N (2 x 100 mL) and brine (2 x 100 mL). The organic phase was dried over anhydrous $MgSO_4$ and concentrated under reduced pressure. The resulting residue was extensively washed with Et_2O, giving the title product as a white solid (**2a**, 1.98 g, 73%); mp 128-130 ºC; ^1H NMR (400 MHz, $CDCl_3$, 298 K) δ = 7.32-7.18 (m, 20H, Ph), 6.32 (t, J = 5.5 Hz, 2H, H_c), 4.21 (t, J = 8.2 Hz, 2H, H_a), 3.88 (dd, J = 5.5, 8.2 Hz, 4H, H_b), 2.71 (s, 4H, H_d); ^{13}C NMR (100 MHz, $CDCl_3$, 298 K) δ = 168.2 (CO), 141.7 (C), 128.9 (CH), 128.1 (CH), 127.0 (CH), 50.6 (CH), 44.1 (CH_2), 35.0 (CH_2); HRMS (ESI) calcd for $C_{32}H_{33}N_2O_2S$ $[M + H]^+$ 509.2257, found 509.2271.

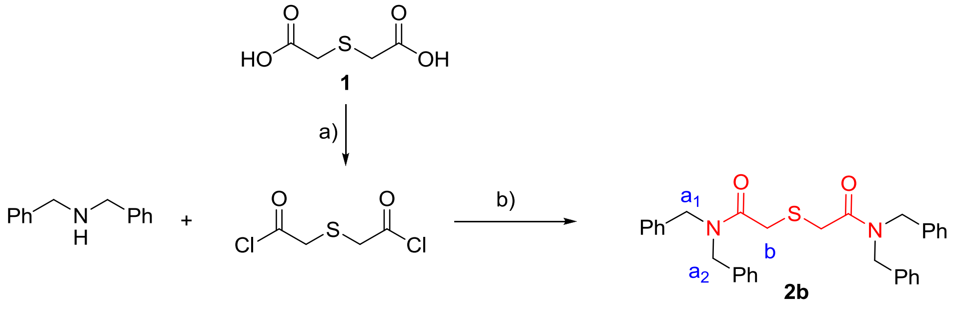

Scheme S2. Synthesis of thread **2b**: a) oxalyl chloride, DMF (cat.), CH_2Cl_2, reflux, 3 h; b) Et_3N, $CHCl_3$, r.t., overnight.

To a suspension of 2,2'-thiodiacetic acid **1** (1.5 g, 10.0 mmol) in dry dichloromethane (40 mL) under N_2 atmosphere was added oxalyl chloride (3.81 g, 30.0 mmol) and DMF (4 drops). The mixture was stirred

for 3 hours under reflux. After this time the solvent and the excess of oxalyl chloride were removed under reduced pressure. The resulting residue was used in the next step without purification.

To a solution of dibenzylamine (4.23 mL, 22 mmol) and Et_3N (4.18 mL, 30 mmol) in $CHCl_3$ (60 mL) was added dropwise the residue obtained in the previous step (1.82 g, 10 mmol), dissolved in 20 mL of $CHCl_3$, during a period of 30 min. The reaction was refluxed overnight. After this time the reaction mixture was washed with water (2 x 100 mL), HCl 1N (2 x 100 mL), NaOH 1N (2 x 100 mL) and brine (2 x 100 mL). The organic phase was dried over anhydrous $MgSO_4$ and concentrated under reduced pressure. The resulting residue was subjected to column chromatography on silica gel using $CHCl_3$/MeOH (99/1) mixture as eluent to give the title product as a white solid (**2b**, 3.98 g, 80%); mp 97-99 ºC; ^{1}H NMR (400 MHz, $CDCl_3$, 298 K) δ = 7.40-7.12 (m, 20H, Ph), 4.62 (s, 4H, H_{a1}), 4.53 (s, 4H, H_{a2}), 3.62 (s, 4H, H_b); ^{13}C NMR (100 MHz, $CDCl_3$, 298 K) δ = 169.5 (CO), 137.1 (C), 136.2 (C), 129.1 (CH), 128.8 (CH), 128.2 (CH), 127.9 (C), 127.6 (C), 126.7 (CH), 50.7 (CH_2), 48.7 (CH_2), 34.1 (CH_2); HRMS (ESI) calcd for $C_{32}H_{33}N_2O_2S$ $[M + H]^+$ 509.2257, found 509.2267.

3. Synthesis of the threads *E*-7

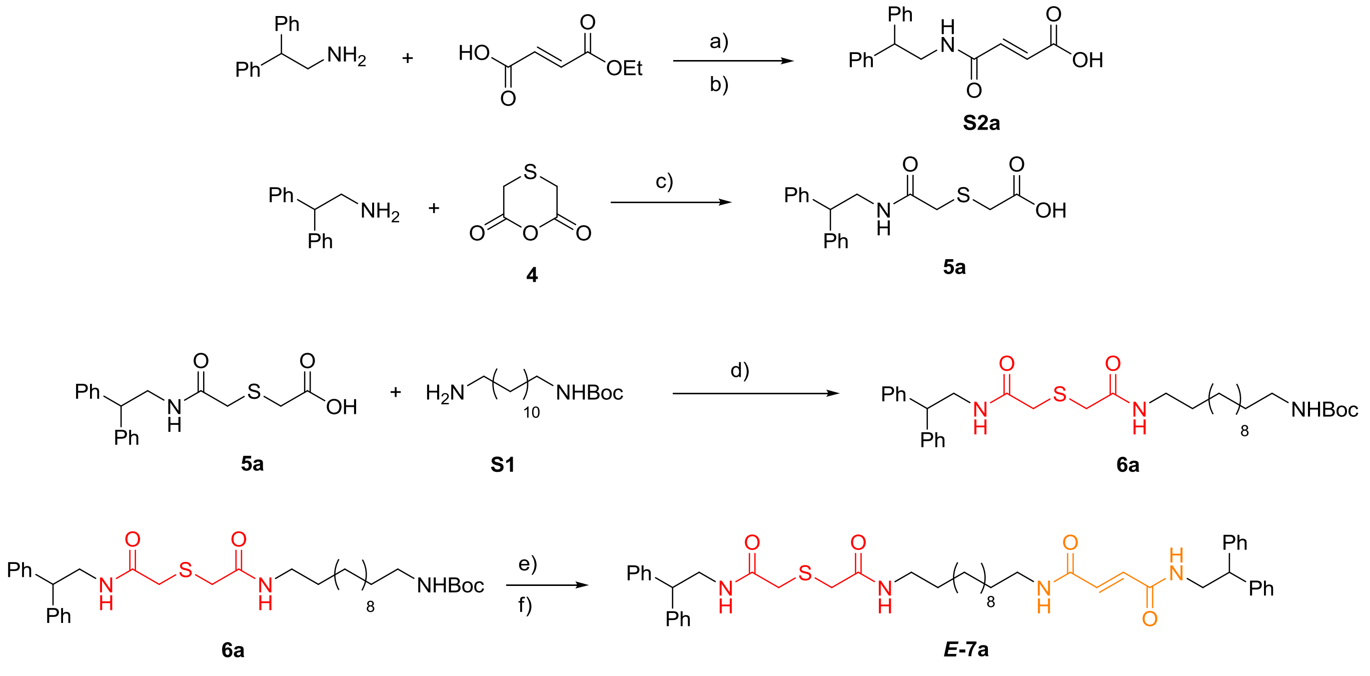

Scheme S3. Synthesis of thread ***E*-7a**: a) DMAP, EDCI, CH_2Cl_2, r.t., 48h; b) NaOH, EtOH, r.t., 12h; c) pyridine, Et_2O, reflux, overnight; d) Et_3N, DMAP, EDCI, CH_2Cl_2, r.t., e) TFA, CH_2Cl_2, r.t., 12 h; f) **S2a**, Et_3N, DMAP, EDCI, CH_2Cl_2, r.t., 48 h.

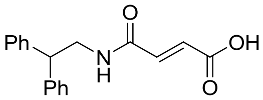

S2a

Fragment **S2a** was synthesized following the described procedure reported in A. Martinez-Cuezva, S. Valero-Moya, M. Alajarin and J. Berna, *Chem. Commun.*, **2015**, *51*, 14501-14504 and showed identical spectroscopic data as those reported therein.

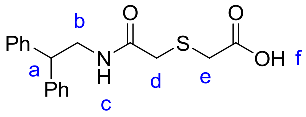

5a

To a solution of 1,4-oxathiane-2,6-dione (500 mg, 3.80 mmol) in dry Et_2O (20 mL) under N_2 atmosphere was added 2,2-diphenylethanamine (820 mg, 4.16 mmol) and pyridine (15.8 mg, 0.20 mmol) and was stirred under reflux for 12 hours. After this time the reaction mixture was neutralized with an aqueous solution of HCl 1M. The aqueous phase was washed with AcOEt (3x 30 mL). The organic phase was dried over anhydrous $MgSO_4$, concentrated under reduced pressure and purified by column chromatography on silica gel using a $CHCl_3$/MeOH (97/3) mixture as eluent to give the title product as a white solid (**5a**, 875 mg, 70%); mp 94-96 ºC; ^1H NMR (400 MHz, $CDCl_3$, 298 K) δ = 7.35-7.20 (m, 10H, Ph), 6.64 (t, *J* = 5.5 Hz, 1H, H_c), 4.25 (t, *J* = 8.1 Hz, 1H, H_a), 3.94 (dd, *J* = 5.5, 8.1 Hz, 2H, H_b), 3.28 (s, 2H, H_e), 2.96 (s, 2H, H_d); ^{13}C NMR (100 MHz, $CDCl_3$, 298 K) δ = 172.5 (CO), 169.4 (CO), 141.5 (C), 129.0 (CH), 128.1 (CH), 127.2 (CH), 50.4 (CH), 44.3 (CH_2), 35.7 (CH_2), 33.4 (CH_2); HRMS (ESI) calcd for $C_{18}H_{18}NO_3S$ [M - H]$^-$ 328.1013, found 328.1003.

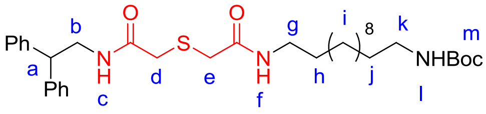

6a

To a suspension of acid **5a** (1.64 g, 5.0 mmol) in dry CH_2Cl_2 (40 mL) under N_2 atmosphere was added amine **S1** (1.00 g, 3.32 mmol) and DMAP (0.49 g, 3.98 mmol) and the mixture was stirred at 0ºC for 10 min. After this time EDCI (0.96 g, 5.0 mmol) was added and the reaction was stirred at room temperature for 48 hours. After this time the reaction mixture was washed with water (2 x 100 mL), HCl 1N (2 x 100 mL), NaOH 1N (2 x 100 mL) and brine (2 x 100 mL), dried over anhydrous $MgSO_4$ and concentrated under reduced pressure. The resulting residue was extensively washed with Et_2O, giving the title product as a white solid (**6a**, 872 mg, 43%); mp 71-73 ºC; ^1H NMR (300 MHz, $CDCl_3$, 298 K) δ = 7.33-7.18 (m, 10H, Ph), 6.97 (t, *J* = 5.6 Hz, 1H, H_f), 6.69 (t, *J* = 5.3 Hz, 1H, H_c), 4.57 (bs, 1H, H_l), 4.26 (t, *J* = 8.1 Hz, 1H, H_a), 3.91 (dd, *J* = 8.1, 5.6 Hz, 2H, H_b), 3.23-3.19 (m, 2H, H_g), 3.10-3.07 (m, 4H, H_{d+k}), 2.81 (s, 2H, H_e), 1.47-1.44 (m, 13H, H_{m+j+h}), 1.28-1.22 (m, 16H, H_i); ^{13}C NMR (75 MHz, $CDCl_3$, 298 K) δ = 168.6

(CO), 168.4 (CO), 156.1 (CO), 141.8 (C), 128.8 (CH), 128.1 (CH), 127.0 (CH), 79.1 (C), 50.6 (CH), 44.2 (CH_2), 40.7 (CH_2), 40.0 (CH_2), 35.6 (CH_2), 35.1 (CH_2), 30.11 (CH_2), 29.6 (CH_2), 29.5 (CH_2), 29.3 (CH_2), 28.5 (CH_3), 27.0 (CH_2), 26.9 (CH_2); HRMS (ESI) calcd for $C_{35}H_{54}N_3O_4S$ $[M + H]^+$ 612.3830, found 612.3849.

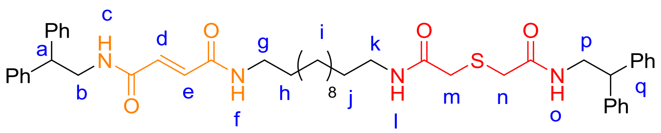

***E*-7a**

To a solution of fragment **6a** (1.41 g, 2.31 mmol) in dry CH_2Cl_2 (30 mL) was added TFA (1.37 mL, 23.1 mmol) and was stirred at room temperature for 12 hours. After this time the solvent and excess of TFA were removed under reduced pressure. The resulting residue was employed in the next step without further purification.

To a solution of the resulting protonated amine (0.82 g, 1.35 mmol) in dry CH_2Cl_2 (35 mL) was added acid **S2a** (0.60 g, 2.04 mmol), Et_3N (0.56 mL, 4.05 mmol) and DMAP (0.20 g, 2.04 mmol). The solution was stirred at 0ºC during 15 min and EDCI (0.392 g, 2.04 mmol) was added. The reaction was stirred at room temperature for 48 hours. After this time the solvent was removed under reduced pressure and the residue was dissolved in a mixture $CHCl_3$:*i*PrOH (3:1) (150 mL). The organic phase was washed with water (2 x 100 mL), HCl 1N (2 x 100 mL), NaOH 1N (2 x 100 mL) and brine (2 x 100 mL). The organic phase was dried over anhydrous $MgSO_4$ and concentrated under reduced pressure. The resulting residue was extensively washed with Et_2O, giving the title product as a white solid (***E*-7a**, 627 mg, 59%); mp 151-153 ºC; ^{1}H NMR (300 MHz, $CDCl_3$ + MeOD, 298 K) δ = 7.31-7.16 (m, 20H, Ph), 6.76 (d, J = 15.2 Hz, 1H, H_e), 6.66 (d, J = 15.2 Hz, 1H, H_d), 4.28-4.22 (m, 2H, H_{a+q}), 3.95-3.85 (m, 4H, H_{b+p}), 3.28-3.11 (m, 4H, H_{g+k}), 3.09 (s, 2H, H_m), 2.84 (s, 2H, H_n), 1.55-1.43 (m, 4H, H_{h+j}), 1.34-1.19 (m, 16H, H_i); signals referred to the NH hydrogens are not observed; ^{13}C NMR (75 MHz, $CDCl_3$ + MeOD) δ = 169.6 (CO), 169.5 (CO), 165.3 (CO), 165.0 (CO), 141.8 (C), 141.7 (C), 132.8 (CH), 132.1 (CH), 128.6 (CH), 128.5 (CH), 127.9 (CH), 127.8 (CH), 126.7 (CH), 126.6 (CH), 50.3 (CH), 50.2 (CH), 44.2 (CH_2), 44.0 (CH_2), 39.7 (CH_2), 35.3 (CH_2), 34.9 (CH_2), 29.4 (CH_2), 29.1 (CH_2), 28.9 (CH_2), 26.8 (CH_2); HRMS (ESI) calcd for $C_{48}H_{61}N_4O_4S$ $[M + H]^+$ 789.4408, found 789.4413.

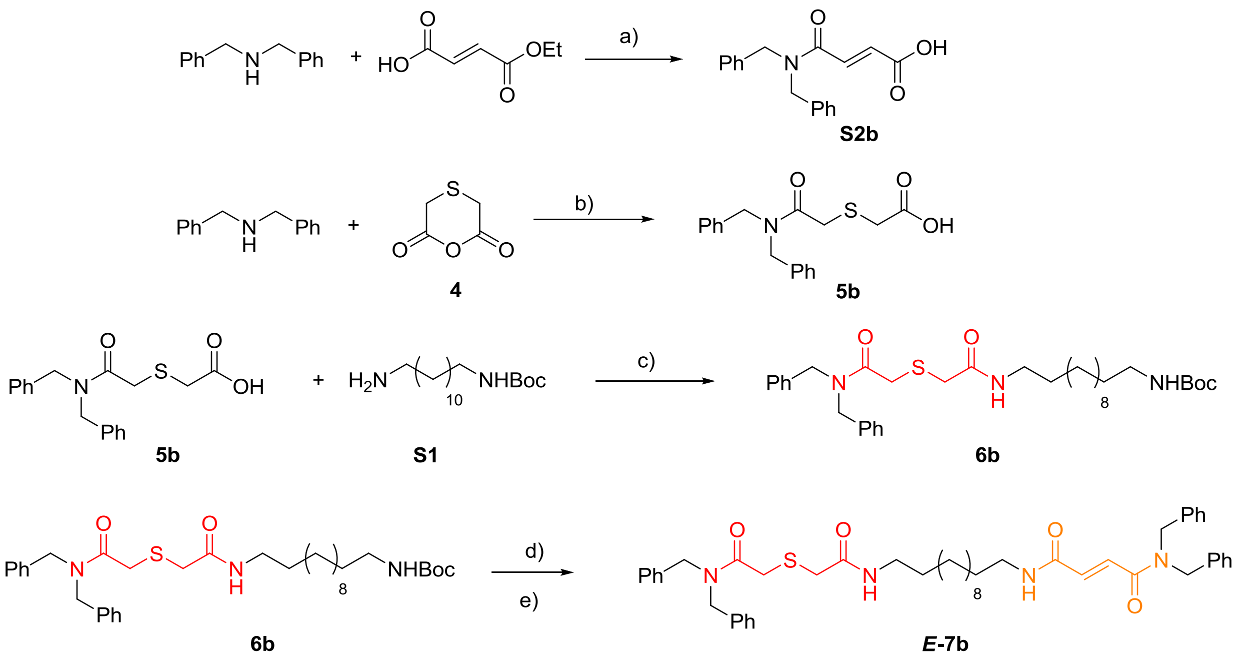

Scheme S4. Synthesis of thread ***E*-7b**: a) TBTU, HOBt, DIPEA, DMF, 0 °C to r.t.; b) pyridine, Et_2O, reflux, overnight; c) Et_3N, DMAP, EDCI, CH_2Cl_2, r.t., d) TFA, CH_2Cl_2, r.t., 12 h; e) **S2b**, Et_3N, DMAP, EDCI, CH_2Cl_2, r.t., 48 h.

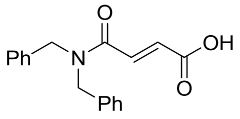

S2b

Fragment **S2b** was synthesized following the described procedure reported in A. Carlone, S. M. Goldup, N. Lebrasseur, D. A. Leigh, A. Wilson, *J. Am. Chem. Soc.,* **2012**, *134*, 8321–8323 and showed identical spectroscopic data as those reported therein.

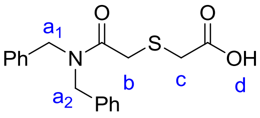

5b

To a solution of 1,4-oxathiane-2,6-dione **4** (500 mg, 3.80 mmol) in dry Et_2O (20 mL) under N_2 atmosphere was added dibenzylamine (0.8 mL, 4.16 mmol) and pyridine (0.3 mL, 0.20 mmol) and was stirred under reflux for 12 hours. After this time the solvent was removed and the residue was dissolved with AcOEt (20 mL). The organic phase was washed with HCl 1N (20 mL), dried over anhydrous $MgSO_4$, concentrated under reduced pressure and purified by column chromatography on silica gel using a $CHCl_3$/MeOH (97/3) mixture as eluent to give the title product as a white solid (**5b**, 1.7 g, 48%); mp 106-108 °C; ^{1}H NMR (200 MHz, DMSO-d_6, 298 K) δ = 12.6 (s, OH_d, 1H), 7.44-7.16 (m, 10H, Ph), 4.56 (s, 2H, H_{a1}), 4.46 (s, 2H, H_{a2}), 3.64 (s, 2H, H_c), 3.39 (s, 2H, H_b); ^{13}C NMR (50 MHz, DMSO-d_6, 298 K) δ = 171.1 (CO), 168.9 (CO), 137.4 (C), 136.9 (C), 128.8 (CH), 128.5 (CH), 127.5 (CH), 127.4 (C), 127.2 (C),

126.9 (CH), 50.4 (CH_2), 47.9 (CH_2), 33.4 (CH_2), 33.3 (CH_2); HRMS (ESI) calcd for $C_{18}H_{20}NO_3S$ $[M + H]^+$ 330.1158, found 330.1146.

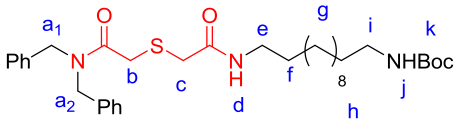

6b

To a suspension of acid **5b** (1.37 g, 4.2 mmol) in dry CH_2Cl_2 (40 mL) under N_2 atmosphere was added amine **S1** (1.5 g, 5 mmol) and DMAP (0.61 g, 5 mmol) and the mixture was stirred at 0ºC for 10 min. After this time EDCI (0.96 g, 5.0 mmol) was added and the reaction was stirred at room temperature for 48 hours. The reaction mixture was washed with water (2 x 100 mL), HCl 1N (2 x 100 mL), NaOH 1N (2 x 100 mL) and brine (2 x 100 mL), dried over anhydrous $MgSO_4$ and concentrated under reduced pressure. The resulting residue was purified by column chromatography on silica gel using a $CHCl_3$/MeOH (40/1) mixture as eluent to give the title product as yellow oil (**6b**, 2.4 g, 94%); ^{1}H NMR (300 MHz, $CDCl_3$, 298 K) δ = 7.46-7.10 (m, 11H, Ph+NH_d), 4.62 (s, 2H, H_{a1}), 4.56-4.47 (m, 3H, H_{a2}+NH_j), 3.44 (s, 2H, H_b), 3.30-3.20 (m, 4H, H_{c+e}), 3.08 (t, J = 6.6 Hz, 2H, H_i), 1.55-1.40 (m, 13H, H_{k+f+h}), 1.30-1.20 (m, 16H, H_g); ^{13}C NMR (75 MHz, $CDCl_3$, 298 K) δ = 169.7 (CO), 168.4 (CO), 156.1 (CO), 136.6 (C), 135.7 (C), 129.2 (CH), 128.9 (CH), 128.1 (C), 128.0 (CH), 127.8 (C), 126.4 (CH), 79.1 (C), 50.8 (CH), 48.8 (CH_2), 40.0 (CH_2), 35.5 (CH_2), 33.3 (CH_2), 30.2 (CH_2), 29.6 (CH_2), 29.4 (CH_2), 29.3 (CH_2), 28.5 (CH_3), 27.1 (CH_2), 26.9 (CH_2); HRMS (ESI) calcd for $C_{35}H_{54}N_3O_4S$ $[M + H]^+$ 612.3830, found 612.3821.

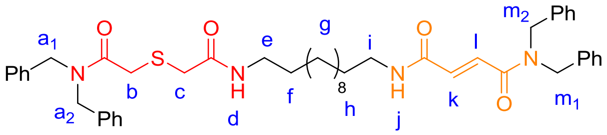

***E*-7b**

To a solution of fragment **6b** (2.4 g, 3.9 mmol) in dry CH_2Cl_2 (50 mL) was added TFA (1.50 mL, 19.5 mmol) and was stirred at room temperature for 12 hours. After this time the solvent and excess of TFA were removed under reduced pressure. The resulting residue was dissolved in CH_2Cl_2:MeOH (1:1) (50 mL) and basic resin Amberlyst® A-21 (1 g) was added. The mixture was stirred for 1 hour. After this time the solution was filtered and the solvent was removed under reduced pressure. The corresponding residue was employed in the next step without further purification.

To a solution of the resulting free amine (0.95 g, 1.85 mmol) in dry CH_2Cl_2 (35 mL) was added acid **S2** (545 mg, 1.85 mmol) and DMAP (225 mg, 1.85 mmol). The solution was stirred at 0ºC during 15 min and EDCI (355 mg, 1.85 mmol) was added. The reaction was stirred at room temperature for 48 hours. After this time the mixture was diluted with CH_2Cl_2 (50 mL) and was washed with water (2 x 100 mL), HCl 1N (2 x 100 mL), NaOH 1N (2 x 100 mL) and brine (2 x 100 mL). The organic phase was dried over anhydrous $MgSO_4$ and concentrated under reduced pressure. The resulting residue was purified by

column chromatography on silica gel using a CH_2Cl_2/MeOH (40/1) mixture as eluent to give the title product as yellow oil (***E*-7b**, 956 mg, 66%); ^{1}H NMR (400 MHz, $CDCl_3$, 298 K) δ = 7.47 (d, J = 14.7 Hz, 1H, H_l), 7.40-7.0 (m, 22H, Ph+H_k+NH_d), 6.42 (t, J = 5.6 Hz, 1H, NH_j), 4.64 (s, 2H, H_{m1}), 4.63 (s, 2H, H_{a1}), 4.56 (s, 2H, H_{m2}), 4.53 (s, 2H, H_{a2}), 3.45 (s, 2H, H_b), 3.32-3.20 (m, 6H, H_{c+e+i}), 1.58-1.43 (m, 4H, H_{f+h}), 1.38-1.19 (m, 16H, H_g); ^{13}C NMR (75 MHz, $CDCl_3$) δ = 169.7 (CO), 168.4 (CO), 166.1 (CO), 164.2 (CO), 136.7 (C), 136.2 (CH), 136.0 (C), 135.8 (C), 129.6 (CH), 129.3 (CH), 129.1 (CH), 128.9 (CH), 128.9 (CH), 128.2 (CH), 128.1 (CH), 128.0 (CH), 127.9 (CH), 127.8 (CH), 127.8 (CH), 126.9 (CH), 126.4 (CH), 50.8 (CH_2), 50.2 (CH_2), 48.8 (CH_2), 48.5 (CH_2), 40.0 (CH_2), 35.6 (CH_2), 33.3 (CH_2), 29.6 (CH_2), 29.6 (CH_2), 29.3 (CH_2), 27.1 (CH_2), 27.0 (CH_2); HRMS (ESI) calcd for $C_{48}H_{61}N_4O_4S$ $[M + H]^+$ 789.4408, found 789.4397.

4. Synthesis of the threads *Z*-7

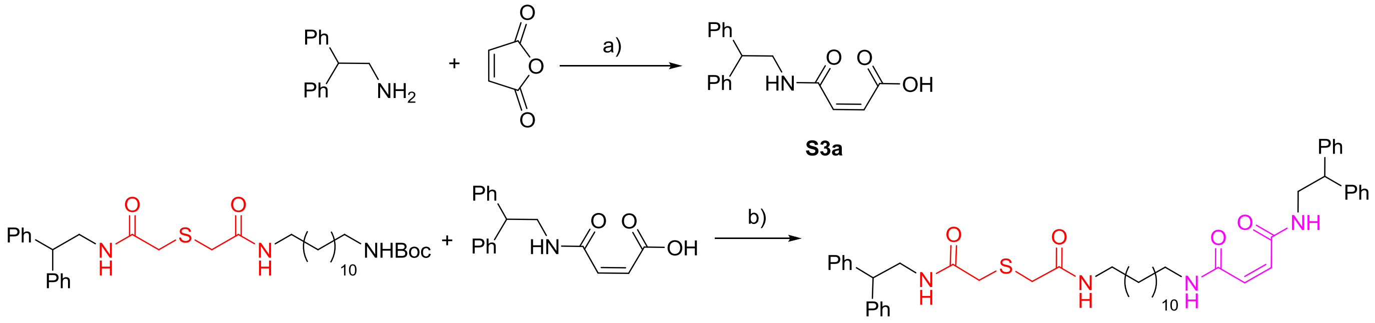

6a S3a *Z*-7a

Scheme S5. Synthesis of thread ***Z*-7a**: a) THF, 0ºC to r.t., 16 hours; c) TFA, CH_2Cl_2, r.t., 12 hours and neutralization; then **S3a,** BOP, Et_3N, CH_2Cl_2, r.t., 5 h.

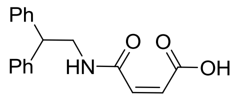

S3a

Fragment **S3a** was synthesized following the described procedure reported in A. Altieri, G. Botteri, F. Dehez, D. A. Leigh, J. K. Y. Wong, F. Zerbetto, *Angew. Chem., Int. Ed.* **2003**, *42*, 2296-2300, and showed identical spectroscopic data as those reported therein.

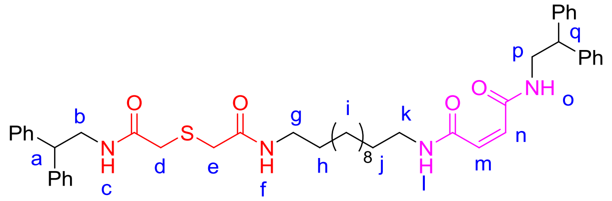

***Z*-7a**

To a solution of fragment **6a** (3.00 g, 3.23 mmol) in dry CH_2Cl_2 (40 mL) was added TFA (2.34 mL, 32.3 mmol) and was stirred at room temperature for 12 hours. After this time the solvent and excess of TFA were removed under reduced pressure. The resulting residue was dissolved in EtOH (30 mL) and

Amberlyst® A21 (3 g) was added. The mixture was stirred at room temperature for 1 hour. The resin was filtered and the solvent was removed under reduced pressure, to give the unprotected amine, which was used in the next step without further purification.

To a suspension of acid **S3a** (0.952 g, 3.23 mmol) in dry CH_2Cl_2 (40 mL) under N_2 atmosphere was added unprotected amine (1.00 g, 3.32 mmol), Et_3N (0.7 mL, 5.09 mmol) and BOP (2.25 g, 5.09 mmol) and the reaction mixture was stirred at room temperature for 5 hours. After this time the reaction mixture was diluted with CH_2Cl_2 (60 mL) and washed with water (2 x 100 mL), HCl 1N (2 x 100 mL), NaOH 1N (2 x 100 mL) and brine (2 x 100 mL), dried over anhydrous $MgSO_4$ and concentrated under reduced pressure. The resulting residue was extensively washed with Et_2O, giving the title product as a white solid (***Z*-7a,** 1.22 mg, 48%); mp 125-127 ºC; ^{1}H NMR (300 MHz, $CDCl_3$, 298 K) δ = 8.72-8.68 (bs, 1H, H_o), 8.20-8.16 (bs, 1H, H_l), 7.33-7.18 (m, 20H, Ph), 6.95 (t, *J* = 6.0 Hz, 1H, H_c), 6.67 (t, *J* = 6.0 Hz, 1H, H_f), 6.01-5.90 (m, 2H, H_{m+n}), 4.26-4.23 (m, 2H, H_{a+q}), 3.94-3.88 (m, 4H, H_{b+p}), 3.26-3.16 (m, 4H, H_{g+k}), 3.08 (s, 2H, H_e), 2.79 (s, 2H, H_d), 1.53-1.46 (m, 4H, H_{h+j}), 1.33-1.22 (bs, 16H, H_i); ^{13}C NMR (75 MHz, $CDCl_3$) δ = 168.5 (CO), 168.4 (CO), 165.1 (CO), 164.7 (CO), 141.9 (C), 141.7 (C), 133.5 (CH), 131.5 (CH), 128.8 (CH), 128.7 (CH), 128.1 (CH), 127.0 (CH), 126.9 (CH), 50.5 (CH), 50.3 (CH), 44.3 (CH_2), 44.1 (CH_2), 40.0 (CH_2), 35.5 (CH_2), 35.1 (CH_2), 29.4 (CH_2), 29.2 (CH_2), 29.1 (CH_2), 27.0 (CH_2), 26.9 (CH_2); HRMS (ESI) calcd for $C_{48}H_{61}N_4O_4S$ $[M + H]^+$ 789.4408, found 789.4428.

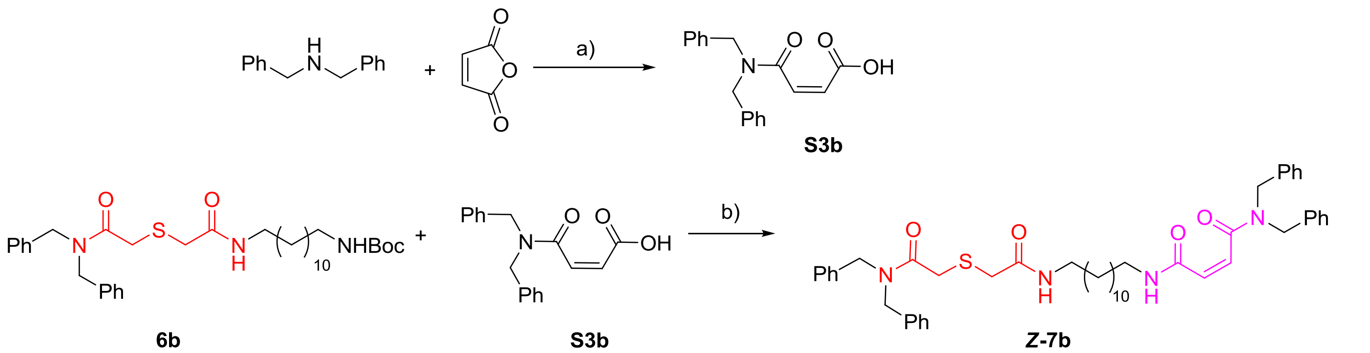

Scheme S6. Synthesis of thread ***Z*-7b**: a) THF, 0ºC to r.t., 16 hours; c) TFA, CH_2Cl_2, r.t., 12 hours and neutralization; then **S3b**, BOP, Et_3N, CH_2Cl_2, r.t., 5 h.

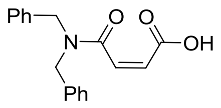

S3b

Fragment **S3b** was synthesized following the described procedure reported in S. Rehn, A. R. Ofial, K. Polborn, H. Mayr, *ARCHIVOC* **2004**, 120-131, and showed identical spectroscopic data as those reported therein.

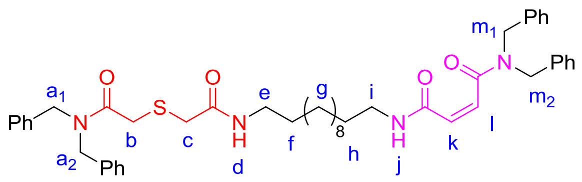

***Z*-7b**

To a solution of the resulting free amine (0.95 g, 1.85 mmol) in dry CH_2Cl_2 (35 mL) was added acid **S3b** (545 mg, 1.85 mmol) and BOP (817 mg, 2.22 mmol). The reaction was stirred at room temperature for 5 hours. After this time the mixture was diluted with CH_2Cl_2 (50 mL) and was washed with water (2 x 100 mL), HCl 1N (2 x 100 mL), NaOH 1N (2 x 100 mL) and brine (2 x 100 mL). The organic phase was dried over anhydrous $MgSO_4$ and concentrated under reduced pressure. The resulting residue was purified by column chromatography on silica gel using a CH_2Cl_2/MeOH (80/1) mixture as eluent to give the title product as yellow oil (***Z*-7b**, 770 mg, 53%); ^{1}H NMR (400 MHz, $CDCl_3$, 298 K) δ = 8.19 (t, J = 5.4 Hz, 1H, NH_j), 7.40-7.10 (m, 21H, Ph+NH_d), 6.43 (d, J = 13.0 Hz, 1H, H_l), 6.15 (d, J = 13.0 Hz, 1H, H_k), 4.63 (s, 2H, H_{a1}), 4.61 (s, 2H, H_{m1}), 4.52 (s, 2H, H_{a2}), 4.46 (s, 2H, H_{m2}), 3.44 (s, 2H, H_b), 3.33-3.202 (m, 6H, H_{c+e+i}), 1.60-1.45 (m, 4H, H_{f+h}), 1.38-1.23 (m, 16H, H_g); ^{13}C NMR (100 MHz, $CDCl_3$) δ = 169.6 (CO), 168.3 (CO), 167.9 (CO), 164.8 (CO), 136.6 (C), 136.1 (C), 135.7 (C), 135.4 (C), 133.1 (CH), 129.2 (CH), 129.2 (CH), 128.9 (CH), 128.8 (CH), 128.6 (CH), 128.3 (CH), 128.1 (CH), 128.0 (CH), 127.9 (CH), 127.8 (CH), 127.7 (CH), 126.9 (CH), 126.4 (CH), 50.8 (CH_2), 50.78 (CH_2), 48.8 (CH_2), 47.6 (CH_2), 40.0 (CH_2), 39.8 (CH_2), 35.5 (CH_2), 33.2 (CH_2), 29.6 (CH_2), 29.5 (CH_2), 29.4 (CH_2), 29.3 (CH_2), 27.1 (CH_2); HRMS (ESI) calcd for $C_{48}H_{61}N_4O_4S$ $[M + H]^+$ 789.4408, found 789.4387.

5. General procedure for the preparation of the [2]rotaxanes 3 and *E*-8

The thread (1 equiv.) and Et_3N (24 equiv.) in anhydrous $CHCl_3$ (250 mL) were stirred vigorously whilst solutions of *p*-xylylenediamine (8 equiv.) in anhydrous $CHCl_3$ (20 mL) and isophthaloyl chloride (8 equiv.) in anhydrous $CHCl_3$ (20 mL) were simultaneously added over a period of 4 h using motor-driven syringe pumps. After a further 4 h the resulting suspension was filtered through a Celite® pad, washed with water (2 x 50 mL), an aqueous solution of HCl 1N (2 x 50 mL), a saturated solution of $NaHCO_3$ (2 x 50 mL) and brine (2 x 50 mL). The organic phase was dried over $MgSO_4$ and the solvent removed under reduced pressure. The resulting solid was subjected to column chromatography (silica gel) to yield unconsumed thread and [2]rotaxane.

Rotaxane 3a

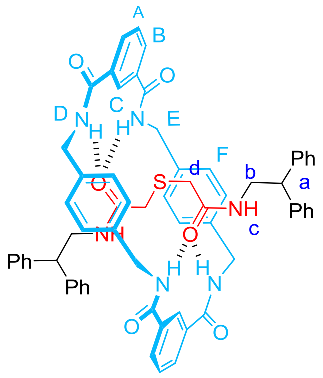

Rotaxane **3a** was obtained following the described method from thread **2a** (1.00 g, 1.96 mmol). The solid crude was subjected to column chromatography on silica gel using $CHCl_3$/MeOH (97/3) mixture as eluent to give the title product as a white solid (**3a**, 264 mg, 13%); mp 115-117 ºC; ^{1}H NMR (300 MHz, $CDCl_3$, 298 K) δ = 8.13-8.07 (m, 6H, H_{B+C}), 7.56 (t, J = 7.7 Hz, 2H, H_A), 7.46 (t, J = 5.0 Hz, 4H, H_D), 7.31-7.10 (m, 20H, Ph), 6.94 (s, 8H, H_F), 6.40 (t, J = 5.0 Hz, 2H, H_c), 4.45 (d, J = 5.0 Hz, 8H, H_E), 3.99 (t, J = 8.1 Hz, 2H, H_a), 3.53 (t, J = 8.1, 5.0 Hz, 4H, H_b), 1.91 (s, 4H, H_d); ^{13}C NMR (75 MHz, $CDCl_3$, 298 K) δ = 169.4 (CO), 167.1 (CO), 141.8 (C), 137.6 (C), 134.6 (C), 131.0 (CH), 129.3 (CH), 129.1 (CH), 129.0 (CH), 128.1 (CH), 127.2 (CH), 125.5 (CH), 66.0 (CH_2), 50.3 (CH), 44.6 (CH_2), 15.4 (CH_2); HRMS (ESI) calcd for $C_{64}H_{61}N_6O_6S$ $[M + H]^+$ 1041.4368, found 1041.4372.

Rotaxane 3b

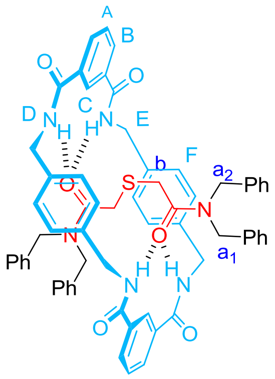

Rotaxane **3b** was obtained following the described method from thread **2b** (1.00 g, 1.96 mmol). The solid crude was subjected to column chromatography on silica gel using $CHCl_3$/MeOH (97/3) mixture as eluent to give the title product as a white solid (**3b**, 224 mg, 11%); mp 165-167 ºC; ^{1}H NMR (400 MHz, $CDCl_3$, 298 K) δ = 8.38 (s, 2H, H_C), 8.13 (d, J = 7.5 Hz, 4H, H_B), 7.52-7.43 (m, 6H, H_{A+D}), 7.32-7.23 (m, 12H, Ph), 7.18-7.14 (m, 4H, Ph), 6.95 (s, 8H, H_F), 6.83-6.77 (m, 4H, Ph), 4.46 (d, J = 5.1 Hz, 8H, H_E), 4.41 (s, 4H, H_{a1}), 4.17 (s, 4H, H_{a2}), 2.55 (s, 4H, H_d); ^{13}C NMR (100 MHz, $CDCl_3$, 298 K) δ = 170.8 (CO), 166.2

(CO), 137.9 (C), 136.2 (C), 134.9 (C), 134.0 (C), 131.8 (CH), 129.3 (CH), 129.2 (CH), 129.1 (CH), 129.0 (CH), 128.4 (CH), 128.2 (CH), 126.5 (CH), 123.8 (CH), 51.5 (CH_2), 49.8 (CH_2), 44.0 (CH_2), 35.1 (CH_2); HRMS (ESI) calcd for $C_{64}H_{61}N_6O_6S$ $[M + H]^+$ 1041.4368, found 1041.4353.

Rotaxane *E*-8a

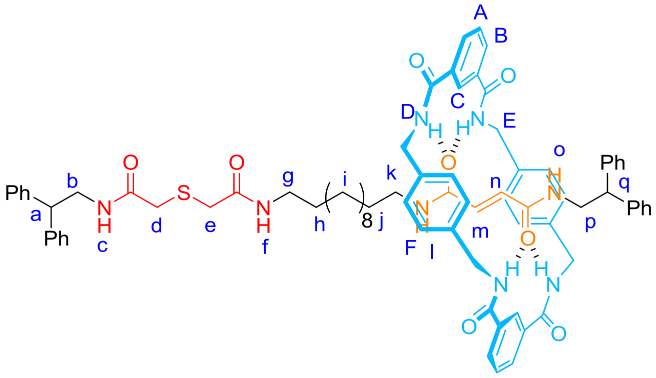

Rotaxane ***E*-8a** was obtained following the described method from thread ***E*-7a** (0.4 g, 0.51 mmol), using a mixture $CHCl_3$:MeCN (10:1) as solvent, due to the low solubility of thread ***E*-7a** in pure $CHCl_3$. The solid crude was subjected to column chromatography on silica gel using $CHCl_3$/MeOH (97/3) mixture as eluent to give the title product as a white solid (***E*-8a**, 168 mg, 25%); mp 103-105 ºC; ^{1}H NMR (300 MHz, $CDCl_3$, 298 K) δ = 8.29 (s, 2H, H_C), 8.15 (bs, 2H, NH), 8.02 (d, J = 9.0 Hz, 4H, H_B), 7.86 (bs, 2H, NH), 7.54 (t, J = 9.0 Hz, 2H, H_A), 7.31-7.14 (m, 20H, Ph), 6.97 (s, 8H, H_F), 5.83 (d, J = 15.0 Hz, 1H, H_m), 5.74 (d, J = 15.0 Hz, 1H, H_n), 4.41 (s, 8H, H_E), 4.30-4.21 (m, 2H, H_{a+q}), 3.89-3.85 (m, 4H, H_{b+p}), 3.24-3.07 (m, 6H, H_{e+g+k}), 2.89 (s, 2H, H_d), 1.53-1.41 (m, 4H, H_{h+j}), 1.25-1.14 (m, 16H, H_i); ^{13}C NMR (75 MHz, $CDCl_3$, 298 K) δ = 168.8 (CO), 168.7 (CO), 166.9 (CO), 166.1 (CO), 165.7 (CO), 141.8 (C), 141.7 (C), 136.9 (C), 133.6 (C), 131.4 (CH), 129.3 (CH), 129.0 (CH), 128.2 (CH), 128.0 (CH), 127.3 (CH), 127.1 (CH), 125.0 (CH), 50.7 (CH_2), 50.6 (CH_2), 45.1 (CH_2), 44.5 (CH_2), 44.3 (CH_2), 40.3 (CH_2), 40.1 (CH_2), 40.0 (CH_2), 35.9 (CH_2), 35.4 (CH_2), 29.6-29.3 (CH_2), 27.3 (CH_2), 27.0 (CH_2); HRMS (ESI) calcd for $C_{80}H_{89}N_8O_8S$ $[M + H]^+$ 1321.6519, found 1321.6500.

Rotaxane *E*-8b

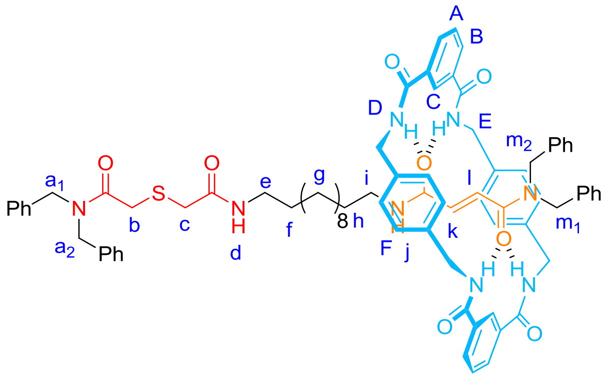

Rotaxane ***E*-8b** was obtained following the described method from thread ***E*-7b** (950 mg, 1.20 mmol). The solid crude was subjected to column chromatography on silica gel using a CH_2Cl_2/AcOEt (1/1) mixture as eluent to elute the unconsumed thread, and then pure AcOEt, to give the title product as a white solid (***E*-8b**, 733 mg, 46%); mp 78-80 ºC; ^{1}H NMR (400 MHz, $CDCl_3$, 298 K) δ = 8.40 (s, 2H, H_C), 8.14 (d, *J* = 7.7 Hz, 4H, H_B), 7.83 (t, *J* = 5.5 Hz, 1H, NH_j), 7.59 (t, *J* = 5.2 Hz, 4H, NH_D), 7.53 (t, *J* = 7.7 Hz, 2H, H_A), 7.40-7.28 (m, 10H, Ph), 7.23-7.03 (m, 9H, Ph+NH_d), 6.94 (s, 8H, H_F), 6.69-6.63 (m, 2H, Ph), 5.97 (s, 2H, H_{k+l}), 4.61 (s, 2H, H_{a1}), 4.52 (s, 2H, H_{a2}), 4.33 (s, 2H, H_{m1}), 4.22 (s, 2H, H_{m2}), 4.50-4.30 (m, 8H, H_E), 3.40 (s, 2H, H_b), 3.28-3.16 (m, 6H, H_{c+e+i}), 1.62-1.46 (m, 4H, H_{f+h}), 1.38-1.19 (m, 16H, H_g); ^{13}C NMR (100 MHz, $CDCl_3$, 298 K) δ = 169.7 (CO), 168.6 (CO), 166.3 (CO), 165.9 (CO), 165.5 (CO), 137.5 (C), 136.7 (C), 136.0 (C), 135.8 (C), 134.6 (C), 133.7 (C), 133.6 (CH), 131.7 (CH), 129.4 (CH), 129.3 (CH), 129.1 (CH), 129.0 (CH), 128.9 (CH), 128.5 (CH), 128.3 (CH), 128.1 (CH), 128.0 (CH), 127.8 (CH), 126.5 (CH), 126.4 (CH), 125.7 (CH), 123.9 (CH), 51.4 (CH_2), 50.9 (CH_2), 48.9 (CH_2), 44.0 (CH_2), 40.6 (CH_2), 40.0 (CH_2), 35.6 (CH_2), 33.3 (CH_2), 29.5 (CH_2), 29.4 (CH_2), 29.3 (CH_2), 29.0 (CH_2), 27.1 (CH_2), 26.9 (CH_2); HRMS (ESI) calcd for $C_{80}H_{89}N_8O_8S$ $[M + H]^+$ 1321.6519, found 1321.6542.

6. Photoisomerization of the molecular shuttles 8

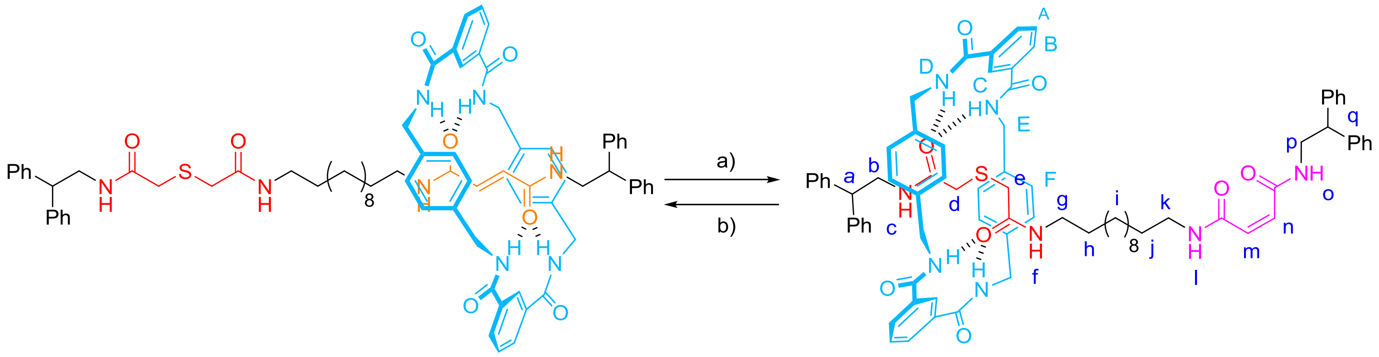

Scheme S7. a) 254 nm, CH_2Cl_2, *Z*-**8a**, 49%; b) 312 nm, CH_2Cl_2, ***E*-8a**, 46%.

Rotaxane ***E*-8a** (100 mg, 0.075 mmol) in anhydrous CH_2Cl_2 (150 mL) was stirred vigorously under N_2 bubbling. The solution was irradiated with a 312 nm lamp during 40 min at room temperature. After this time, the solvent was removed under reduced pressure. The resulting solid was subjected to column chromatography (silica gel) using a $CHCl_3$:MeOH (97:3) mixture as eluent, to recovered unconsumed ***E*-8a** and ***Z*-8a** (***Z*-8a**, 49 mg, 49%); mp 106-108 ºC; ^{1}H NMR (400 MHz, $CDCl_3$, 298 K) δ = 8.92 (bs, 1H, NH), 8.42 (s, 2H, H_C), 8.20-8.08 (m, 5H, H_B + NH), 7.72 (bs, 4H, H_D), 7.54 (t, *J* = 7.1 Hz, 2H, H_A), 7.27-7.14 (m, 20H, Ph), 7.10-7.00 (m, 9H, H_F + NH), 6.81 (s, 1H, NH), 5.83 (s, 2H, H_{m+n}), 4.66-4.25 (m, 8H, H_E), 4.18-4.13 (m, 2H, H_{a+q}), 3.78-3.73 (m, 4H, H_{b+p}), 2.98-2.96 (m, 2H, H_k), 2.70-2.51 (m, 6H, H_{d+e+g}), 1.34-1.10 (m, 20H, H_{h+i+j}); ^{13}C NMR (75 MHz, $CDCl_3$, 298 K) δ = 169.1 (CO), 166.6 (CO), 165.9 (CO),

164.6 (CO), 141.9 (C), 141.3 (C), 137.4 (C), 133.9 (C), 131.7 (CH), 129.4 (CH), 129.3 (CH), 129.1 (CH), 129.0 (CH), 128.9 (CH), 128.1 (CH), 127.9 (CH), 127.3 (CH), 127.1 (CH), 50.5 (CH), 50.3 (CH), 44.4 (CH_2), 40.1 (CH_2), 35.2 (CH_2), 29.8 (CH_2), 29.1 (CH_2), 29.1 (CH_2), 29.0 (CH_2), 28.8 (CH_2), 29.3 (CH_2), 26.8 (CH_2); HRMS (ESI) calcd for $C_{80}H_{89}N_8O_8S$ $[M + H]^+$ 1321.6519, found 1321.6501.

The obtained compound ***Z*-8a** was re-isomerized again to the initial co-conformer ***E*-8a** in 46% yield by irradiation at 312 nm of a solution of the *cis* isomer in DCM during 45 min.

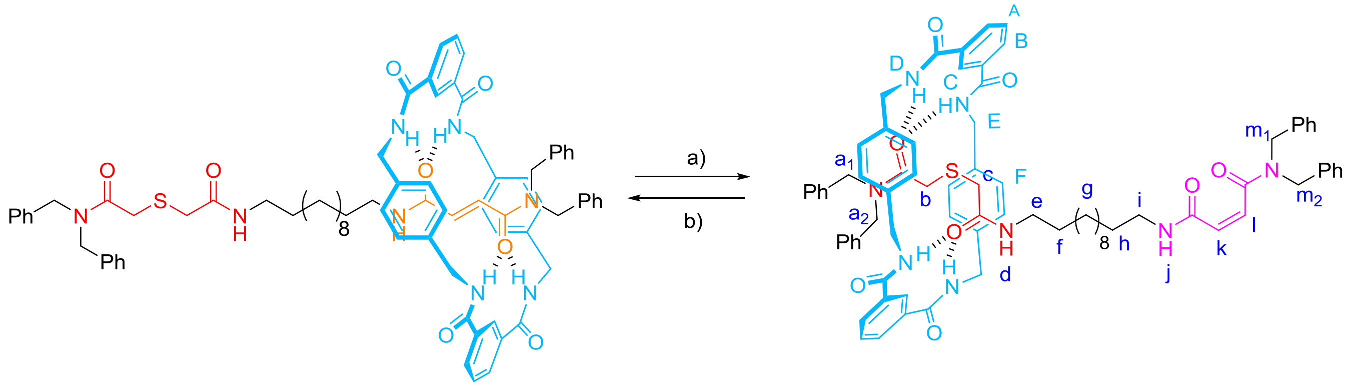

Scheme S8. a) 254 nm, CH_2Cl_2, ***Z*-8b**, 53%; b) 312 nm, CH_2Cl_2, ***E*-8b**, 58%.

Rotaxane ***E*-8b** (100 mg, 0.075 mmol) in anhydrous CH_2Cl_2 (150 mL) was stirred vigorously under N_2 bubbling. The solution was irradiated with a 312 nm lamp during 40 min at room temperature. After this time, the solvent was removed under reduced pressure. The resulting solid was subjected to column chromatography (silica gel) using a $CHCl_3$:acetone (2:1) mixture as eluent, to recovered unconsumed ***E*-8b** and ***Z*-8b** (***Z*-8b**, 53 mg, 53%); mp 99-101ºC; ^{1}H NMR (400 MHz, $CDCl_3$, 298 K) δ = 8.32 (s, 2H, H_C), 8.15 (d, J = 8.6 Hz, 4H, H_B), 7.94 (t, J = 5.2 Hz, 1H, NH_j), 7.65 (t, J = 5.2 Hz, 4H, NH_D), 7.52 (t, J = 7.7 Hz, 2H, H_A), 7.40-7.07 (m, 28H, Ph+H_F), 5.03 (t, J = 5.2 Hz, 1H, NH_d), 6.39 (d, J = 12.8 Hz, 1H, H_l), 5.97 (d, J = 12.8 Hz, 1H, H_k), 4.60 (s, 2H, H_{m1}), 4.55-4.40 (m, 14H, $H_{a1+a2+m2+E}$), 3.04 (s, 2H, H_b), 2.88-2.64 (m, H, H_{e+i}), 2.66 (s, 2H, H_c), 1.20-0.95 (m, 20H, H_{f+g+h}); ^{13}C NMR (100 MHz, $CDCl_3$, 298 K) δ = 170.2 (CO), 169.4 (CO), 168.1 (CO), 166.3 (CO), 164.6 (CO), 138.0 (C), 136.6 (C), 136.0 (C), 135.5 (C), 135.4 (C), 134.1 (C), 132.4 (CH), 131.4 (CH), 129.3 (CH), 129.2 (CH), 129.0 (CH), 128.9 (CH), 128.3 (CH), 128.2 (CH), 128.0 (CH), 127.9 (CH), 127.8 (CH), 127.0 (CH), 126.5 (CH), 124.4 (CH), 51.4 (CH_2), 51.3 (CH_2), 49.1 (CH_2), 48.2 (CH_2), 44.2 (CH_2), 40.3 (CH_2), 39.9 (CH_2), 34.7 (CH_2), 32.9 (CH_2), 29.3 (CH_2), 29.2 (CH_2), 29.0 (CH_2), 26.9 (CH_2); HRMS (ESI) calcd for $C_{80}H_{89}N_8O_8S$ $[M + H]^+$ 1321.6519, found 1321.6561.

The obtained compound ***Z*-8b** was re-isomerized again to the initial co-conformer ***E*-8b** in 58% yield by irradiation at 312 nm of a solution of the *cis* isomer in DCM during 45 min.

7. Chalcogenide-$TiCl_4$ catalyzed Baylis-Hillman reaction

Reaction conditions for this transformation reported in S. Kinoshita, H. Kinoshita, T. Iwamura, S.-I. Watanabe, T. Kataoka, *Chem.-Eur. J.* **2003**, *9*, 1496-1502.

General procedure: To a solution of *p*-nitrobenzaldehyde (3.8 mg, 0.025 mmol), 3-butyn-2-one (5.1 mg, 0.075 mmol) and the corresponding sulfide derivative "S" (0.1 equiv.) in dry CH_2Cl_2 at 0-5ºC was added $TiCl_4$ (0.025 mmol, solution 1M in CH_2Cl_2). The reaction was stirred under nitrogen at the established temperature for 3 hours. After this time a saturated solution of $NaHCO_3$ (0.5 mL) was added. The reaction mixture was diluted with CH_2Cl_2 and extracted with H_2O. The organic phase was dried over $MgSO_4$ and the solvent removed under reduced pressure. The corresponding residue was analyzed by ^1H NMR spectroscopy and the conversion and diastereomeric ratio of ***E*-9** and ***Z*-9** was calculated.

Table S1. Screening of the reaction conditions for the BH transformation of *p*-nitrobenzaldehyde and 3-butyn-2-one.

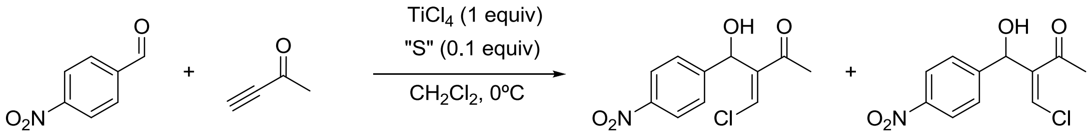

1 equiv. 3 equiv. *E*-9 *Z*-9

"S"	[M]	T (ºC)	time (h)	Conv.[b] (%)[b]	d.r.[b] (*E*:*Z*)
**2a**	0.33	0	2	95	89:11
**2b**	0.33	0	2	96	90:10
**3a**[b]	0.33	0	2	-	-
***E*-8a**[b]	0.33	0	2	-	-
**2a**	0.10	0	2	62	82:8
**2b**	0.10	0	2	70	83:7
**2b**	0.10	5	3	92	91:9

[a] Determined by ^1H NMR; [b] No reaction due to solubility issues.

Table S2. Screening of different sulfur-containing species “S” under the optimized reaction conditions.

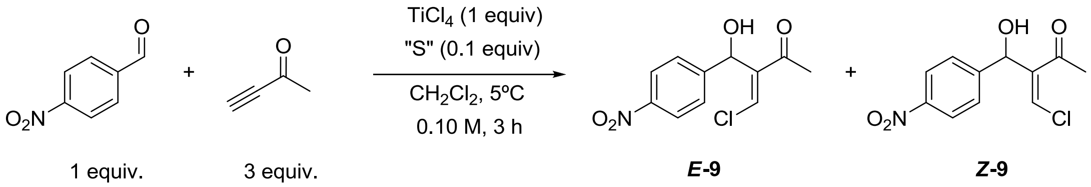

“S”	conv (%)[a]	*E*:*Z*[a]
**2a**	94	85:15
**2b**	92	91:9
**3a**	90	55:45
**3b**	85	50:50
***E*-8a**	70	56:44
***E*-8b**	71	80:20
***Z*-8b**	54	50:50

[a] Determined by ^{1}H NMR.

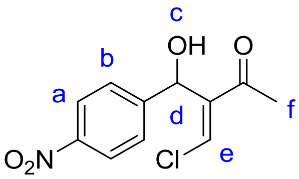

***E*-9**

Compound ***E*-9** was described in S. Kinoshita, H. Kinoshita, T. Iwamura, S.-I. Watanabe, T. Kataoka, *Chem. Eur. J.* **2003**, *9*, 1496-1502, and showed identical spectroscopic data as those reported therein; ^{1}H NMR (300 MHz, $CDCl_3$, 298 K) δ = 8.15 (d, J = 8.8 Hz, 2H, H_a), 7.57-7.51 (m, 3H, H_{b+e}), 6.01 (d, J = 11.2 Hz, 1H, H_d), 4.41 (d, J = 11.2 Hz, 1H, OH_c), 2.34 (s, 3H, H_f).

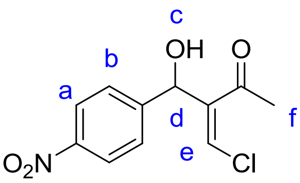

***Z*-9**

Compound ***Z*-9** was described in S. Kinoshita, H. Kinoshita, T. Iwamura, S.-I. Watanabe, T. Kataoka, *Chem. Eur. J.* **2003**, *9*, 1496-1502, and showed identical spectroscopic data as those reported therein; ^{1}H NMR (300 MHz, $CDCl_3$, 298 K) δ = 8.20 (d, J = 8.8 Hz, 2H, H_a), 7.53 (dd, J = 0.6, 8.8 Hz, 2H, H_b), 6.80 (d, J = 1.0 Hz, 1H, H_e), 5.61 (d, J = 4.0 Hz, 1H, H_d), 3.52 (d, J = 4.0 Hz, OH_c), 2.44 (s, 3H, H_f).

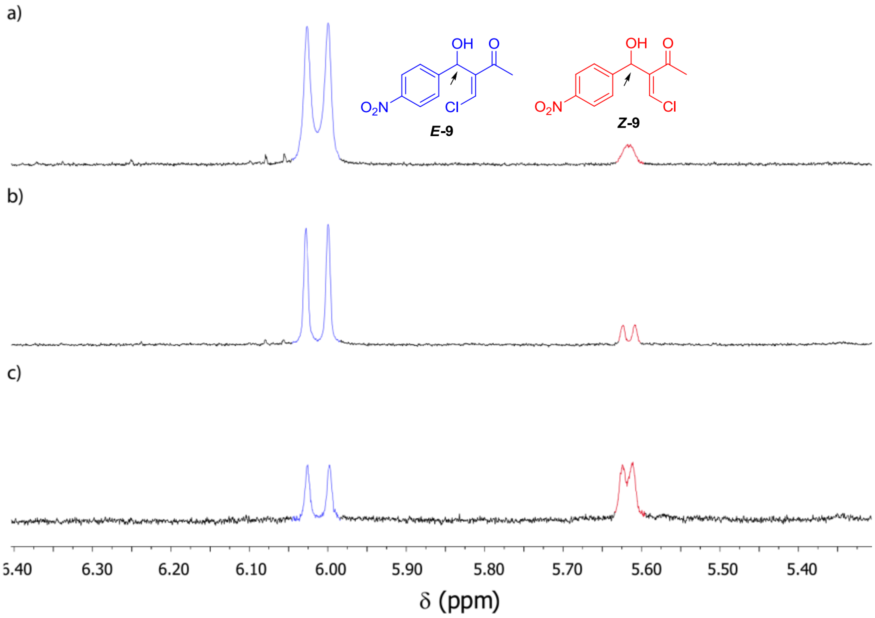

Figure S1. Partial ^{1}H NMR (400 MHz, $CDCl_3$) spectrum of: a) selected signals related to ***E*-9** and ***Z*-9** when thread **2b** was employed as catalyst (d.r.: 91:9); b) when rotaxane ***E*-8b** was employed as catalyst (Catalyst ON, d.r.: 80:20); c) when rotaxane ***Z*-8b** was employed as catalyst (Catalyst OFF, d.r.: 50:50).

8. Crystal data and structure refinement for rotaxane 3a

Table S3. Crystal data and structure refinement for **3a**

	**3a**
Empirical formula	C_{66} H_{62} N_7 O_7 S
Formula weight	1097.29
T [K]	100(2)
Wavelength [Å]	1.54178
Crystal system	Triclinic
Space group	P(-1)
a (Å)	10.2417(8)
b (Å)	17.4702(14)
c (Å)	18.1698(15)
α (°)	117.240(3)
β (°)	90.580(3)
γ (°)	92.649(3)
V [Å^3]	2885.6(4)
Z	2
ρ [g·cm^{-3}]	1.263
μ [mm^{-1}]	0.990
F_{000}	1158
Crystal size [mm^3]	0.22 x 0.17 x 0.04
θ range (º)	2.74-66.67
h	-12 to 12
k	-20 to 20

l	-21 to 21
Reflections collected	88755
Independent reflections	9949
R(int)	0.0363
Refinement method	Full-matrix least-squares on F2
Parameters	735
Restraints	3
Goodness-of-fit on F^2	1.175
$R1$ [I > 2σ(I)]	0.0446
$wR2$ [I > 2σ(I)]	0.1443
$R1$ (all data)	0.0497
$wR2$ (all data)	0.1477
$\Delta\rho$ [e·Å^{-3}]	0.565 /-0.299

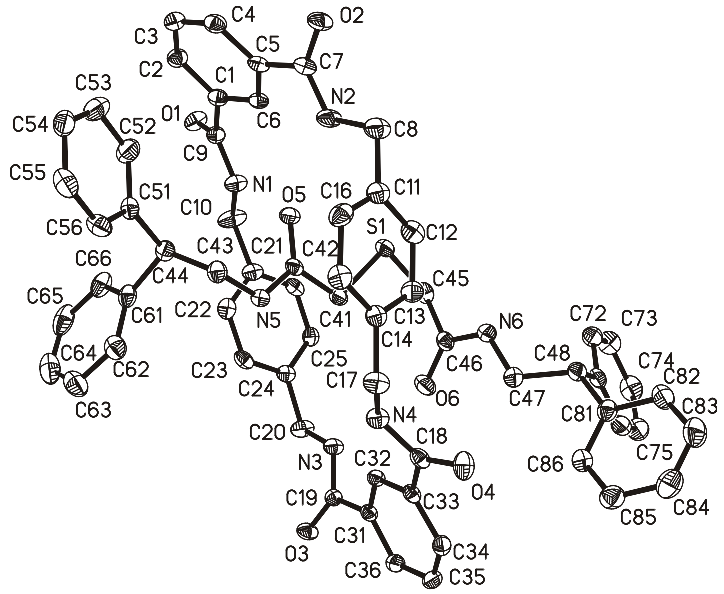

Figure S2. Molecular structure of rotaxane **3a** with thermal ellipsoids drawn at 50% probability.

X-ray Structure Determinations.

A colorless prism-like specimen of $C_{67}H_{66}N_6O_7S$, approximate dimensions 0.040 mm x 0.170 mm x 0.220 mm, was used for the X-ray crystallographic analysis. The X-ray intensity data were measured on a Bruker D8 QUEST system equipped with a multilayer monochromator and a Cu K/a Incoatec microfocus sealed tube (λ = 1.54178 Å).

A total of 2452 frames were collected. The total exposure time was 81.73 hours. The frames were integrated with the Bruker SAINT software package using a narrow-frame algorithm. The integration of the data using a triclinic unit cell yielded a total of 88755 reflections to a maximum θ angle of 66.67° (0.84 Å resolution), of which 9949 were independent (average redundancy 8.921, completeness = 97.5%, R_{int} = 3.63%, R_{sig} = 1.77%) and 8893 (89.39%) were greater than $2\sigma(F^2)$. The final cell constants of a = 10.2417(8) Å, b = 17.4702(14) Å, c = 18.1698(15) Å, α = 117.240(3)°, β = 90.580(3)°, γ = 92.649(3)°,

volume = 2885.6(4) Å^3, are based upon the refinement of the XYZ-centroids of 9037 reflections above 20 σ(I) with 5.699° < 2θ < 133.3°. Data were corrected for absorption effects using the multi-scan method (SADABS). The ratio of minimum to maximum apparent transmission was 0.880. The calculated minimum and maximum transmission coefficients (based on crystal size) are 0.8116 and 0.9615.
The final anisotropic full-matrix least-squares refinement on F^2 with 735 variables converged at R1 = 4.46%, for the observed data and wR2 = 14.77% for all data. The goodness-of-fit was 1.175. The largest peak in the final difference electron density synthesis was 0.565 e^-/Å^3 and the largest hole was -0.299 e^-/Å^3 with an RMS deviation of 0.066 e^-/Å^3. On the basis of the final model, the calculated density was 1.263 g/cm^3 and F(000), 1158 e^-.

Table S4. Hydrogen bonds for rotaxane **3a** [Å and (°)].

D-H...A	d(D-H)	d(H...A)	d(D...A)	<(DHA)
N(1)-H(01)...O(5)	0.88	2.20	3.083(3)	176.8
N(2)-H(02)...O(5)	0.88	2.10	2.976(3)	177.5
N(3)-H(03)...O(6)	0.88	2.10	2.905(3)	152.6
N(4)-H(04)...O(7)#1	0.88	2.27	3.080(3)	153.5
N(5)-H(05)...O(7)#1	0.88	1.96	2.822(3)	165.3
N(6)-H(06)...O(2)#2	0.88	2.02	2.881(3)	164.6
O(7)-H(98)...O(1)#3	0.834(10)	1.948(11)	2.766(2)	167(3)
O(7)-H(99)...O(3)	0.833(10)	1.927(10)	2.760(2)	179(3)
C(6)-H(6)...O(5)	0.95	2.30	2.969(3)	126.9
O(7)-H(98)...C(10)#3	0.834(10)	2.88(3)	3.359(3)	118(2)

Symmetry transformations used to generate equivalent atoms: #1 -x+2,-y+1,-z+1 #2 -x+1,-y,-z #3 -x+1,-y+1,-z+1

A CH···π interaction between one of the methylenic hydrogen atoms of the thread and one of the *p*-xylylene rings of the tetralactam is observed. This interaction is featured by a distance of 3.4 Å between the C41 and the centroid (CD1) and angle of 86º between the C41-H41A bond and the nearest carbon of the centroid (Figure S3).

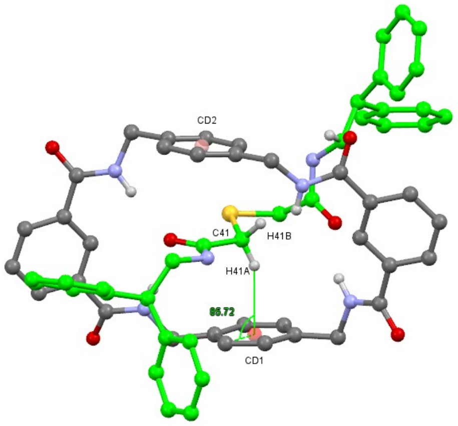

Figure S3. Molecular structure of rotaxane **3a** showing a stabilising established by CH···π interaction (3.4 Å, 86º).

9. ^{1}H and ^{13}C NMR Spectra of synthesized compounds

2a (^{1}H NMR, 400 MHz, $CDCl_3$, 298K)

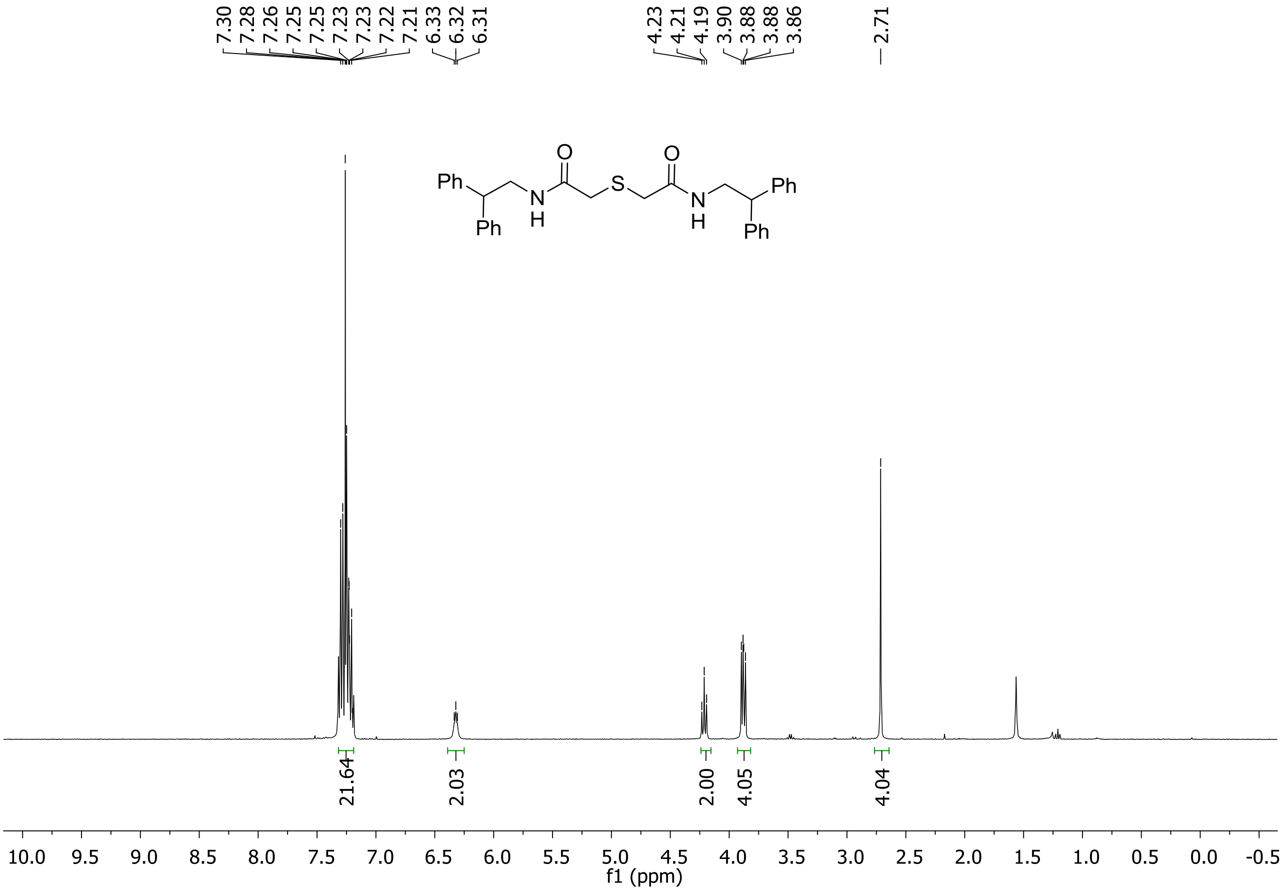

2a (^{13}C NMR, 100 MHz, $CDCl_3$, 298K)

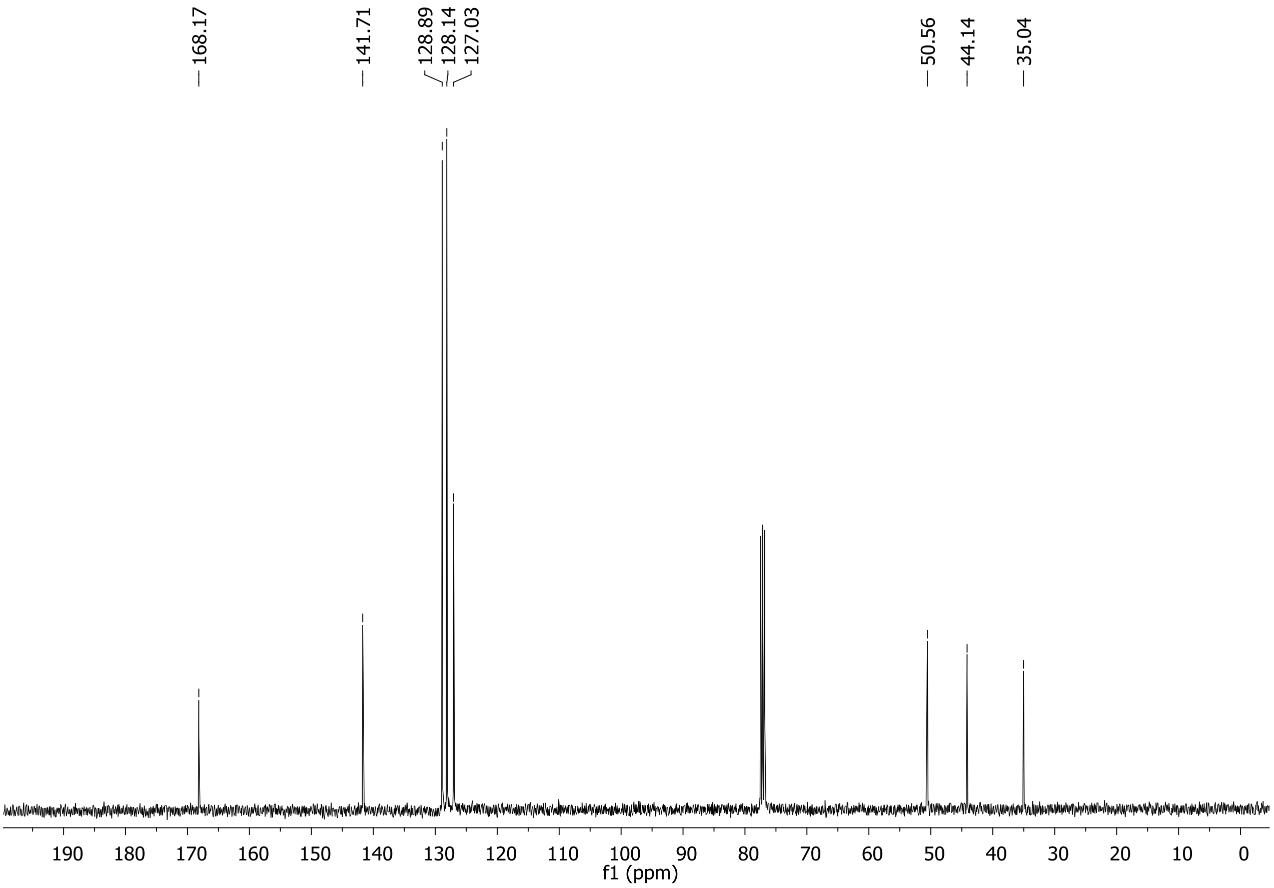

2b (^{1}H NMR, 400 MHz, $CDCl_3$, 298K)

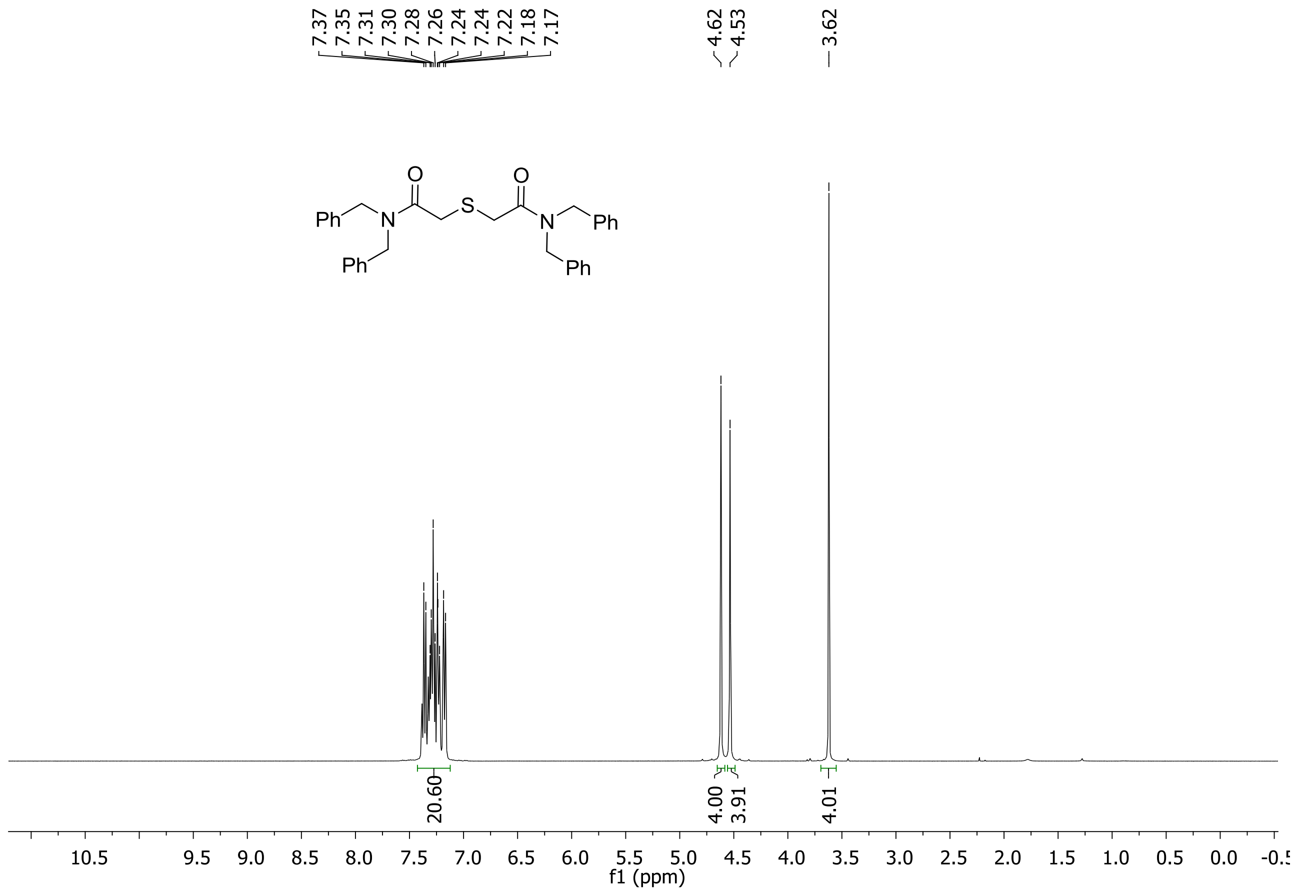

2b (^{13}C NMR, 100 MHz, $CDCl_3$, 298K)

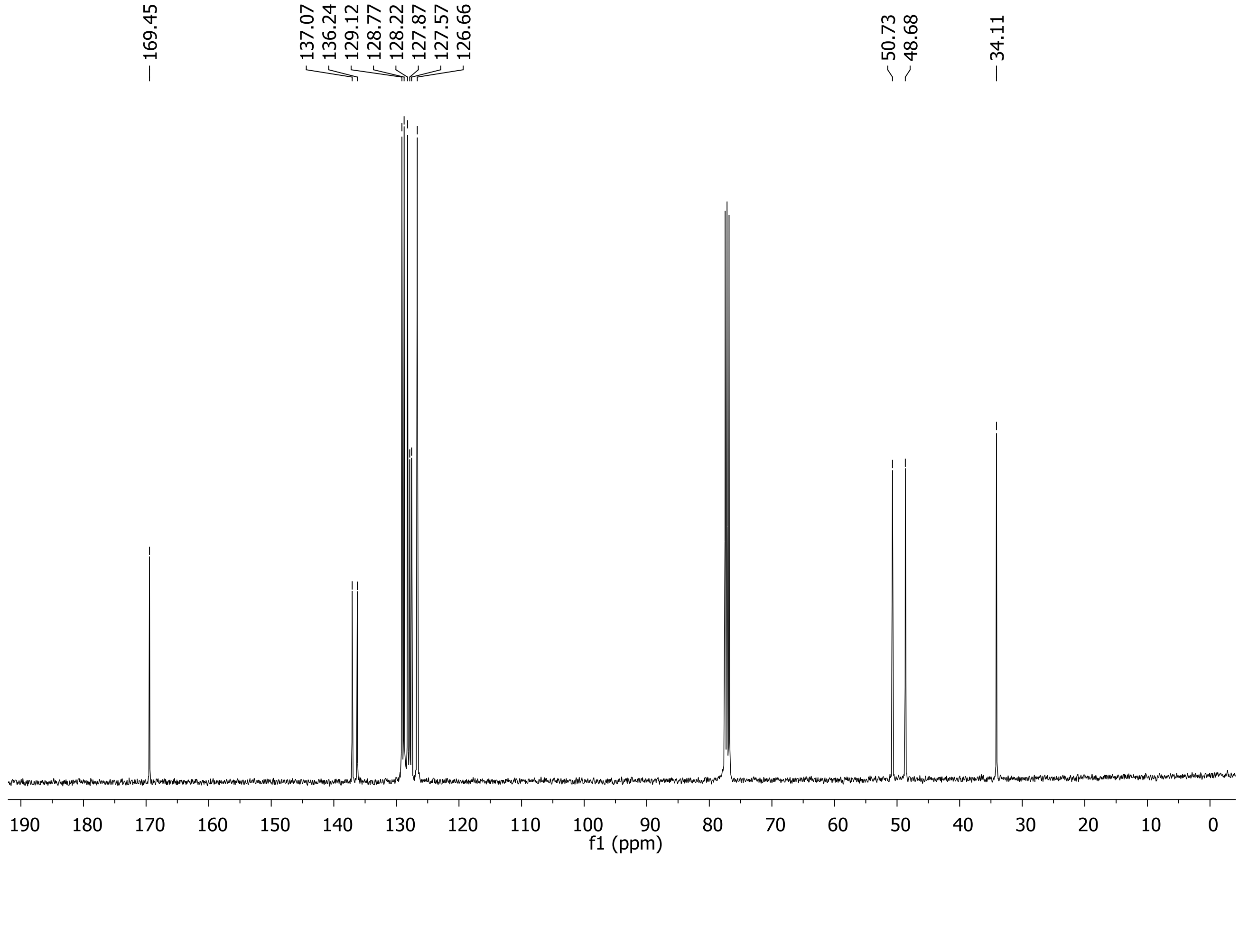

5a (^{1}H NMR, 400 MHz, $CDCl_3$, 298K)

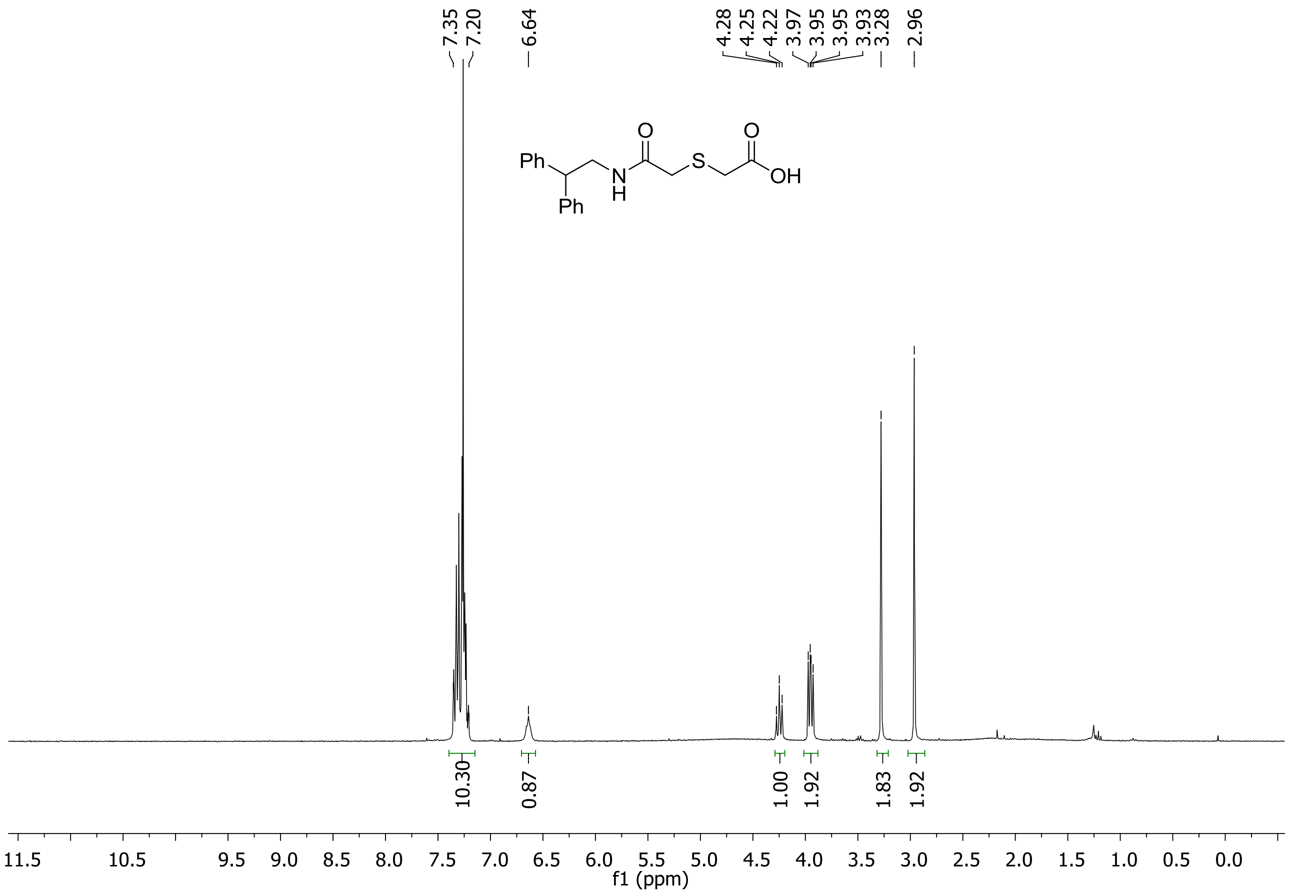

5a (^{13}C NMR, 100 MHz, $CDCl_3$, 298K)

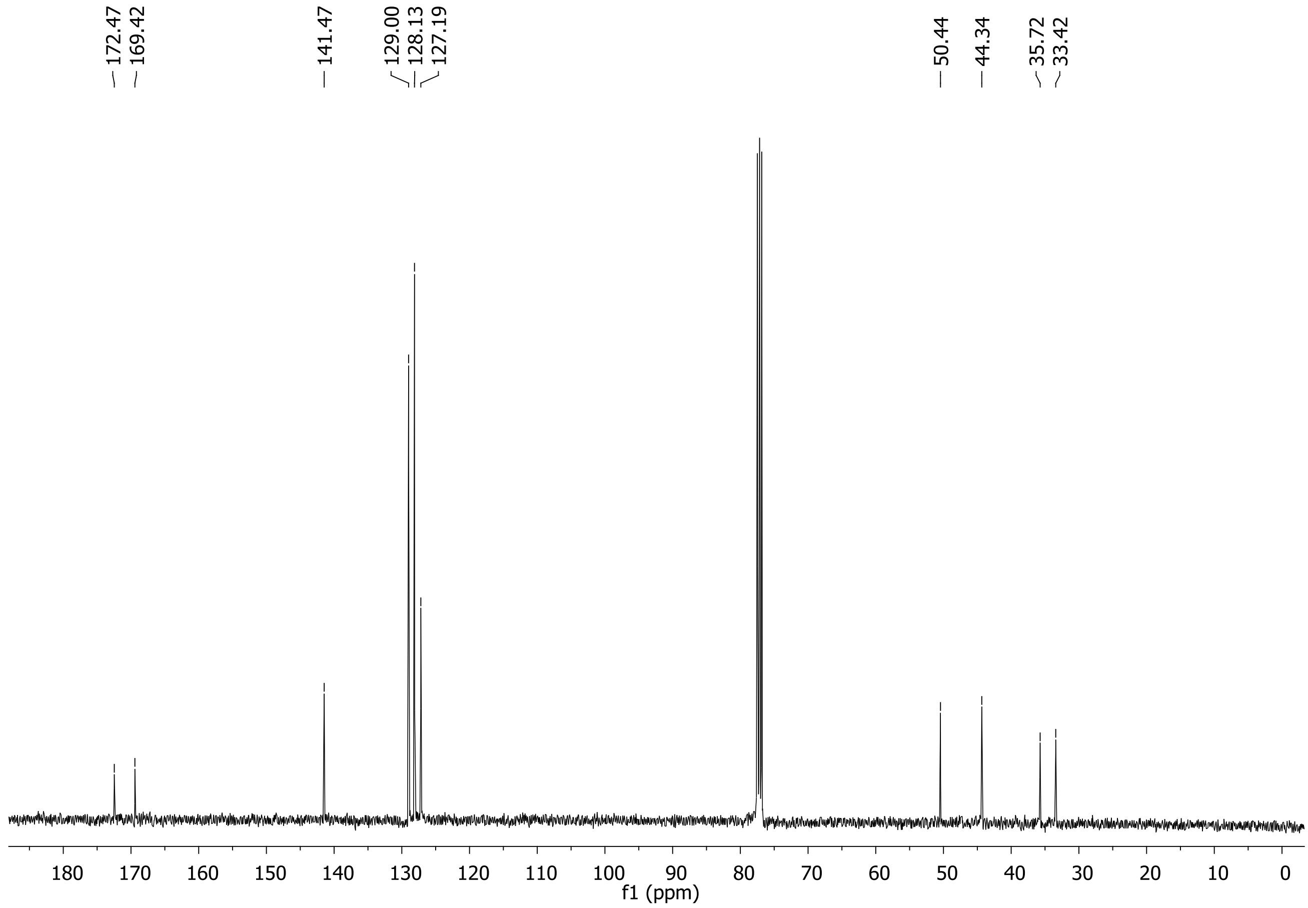

5b (^{1}H NMR, 200 MHz, DMSO-d_6, 298K)

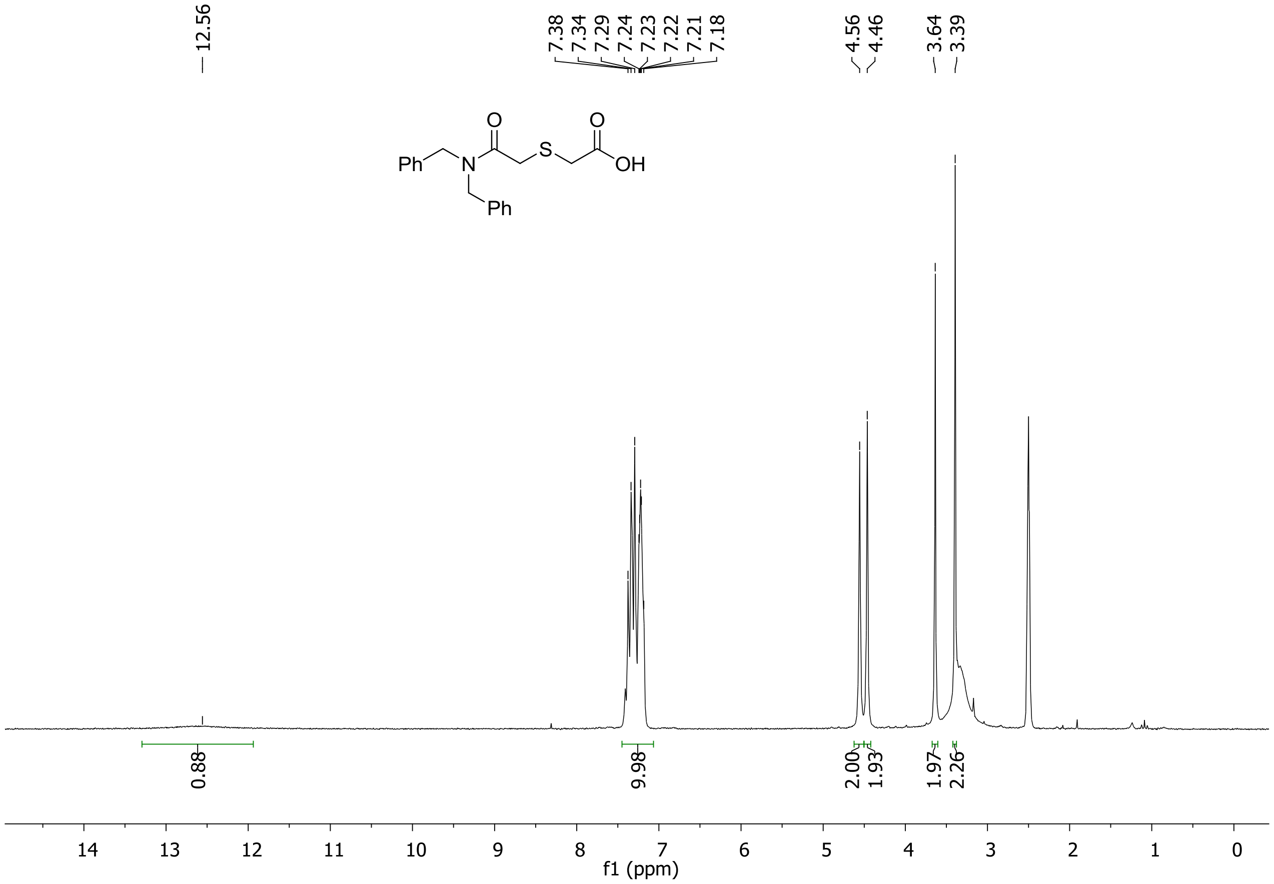

5b (^{13}C NMR, 50 MHz, DMSO-d_6, 298K)

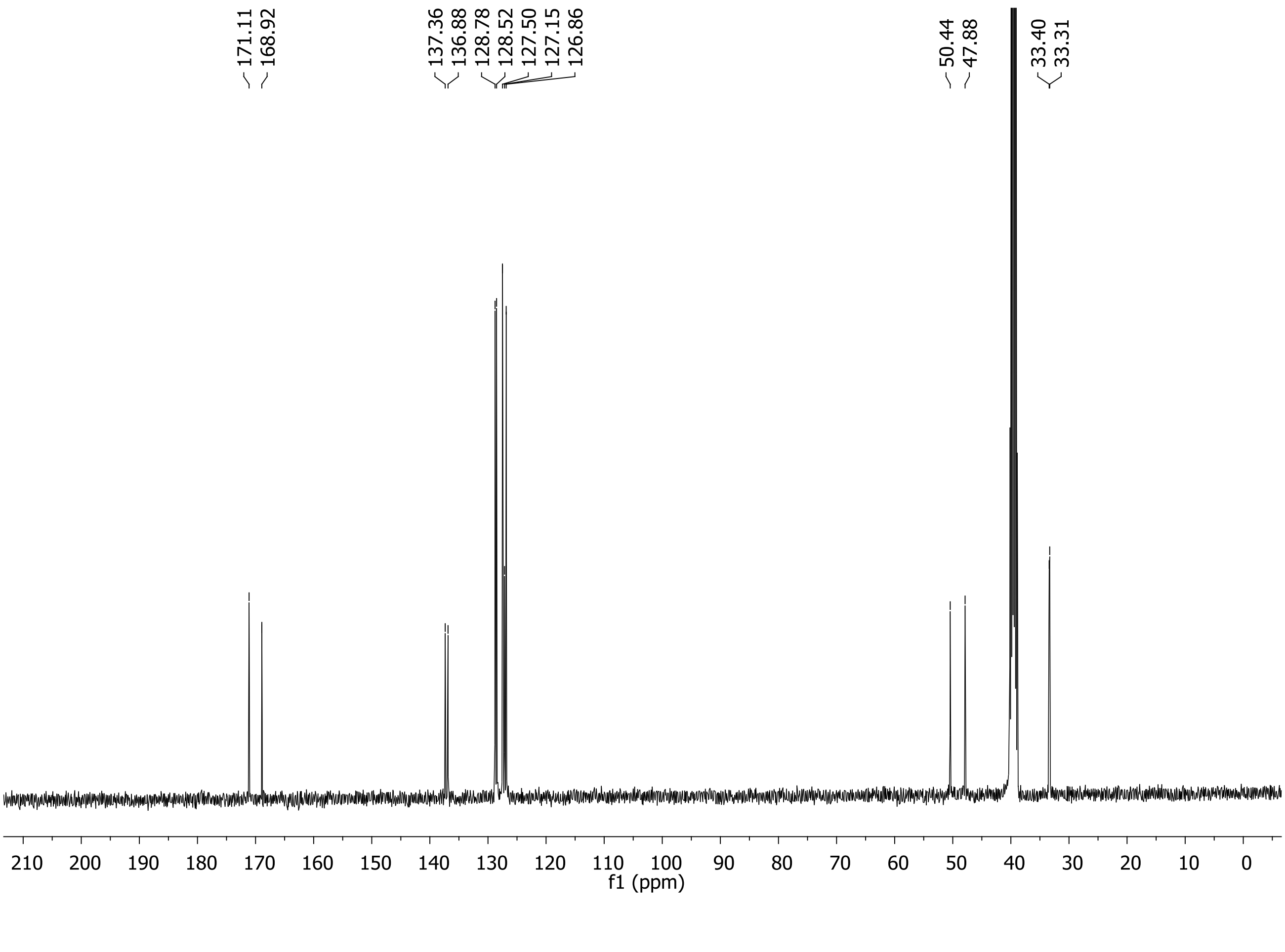

6a (^{1}H NMR, 300 MHz, $CDCl_3$, 298K)

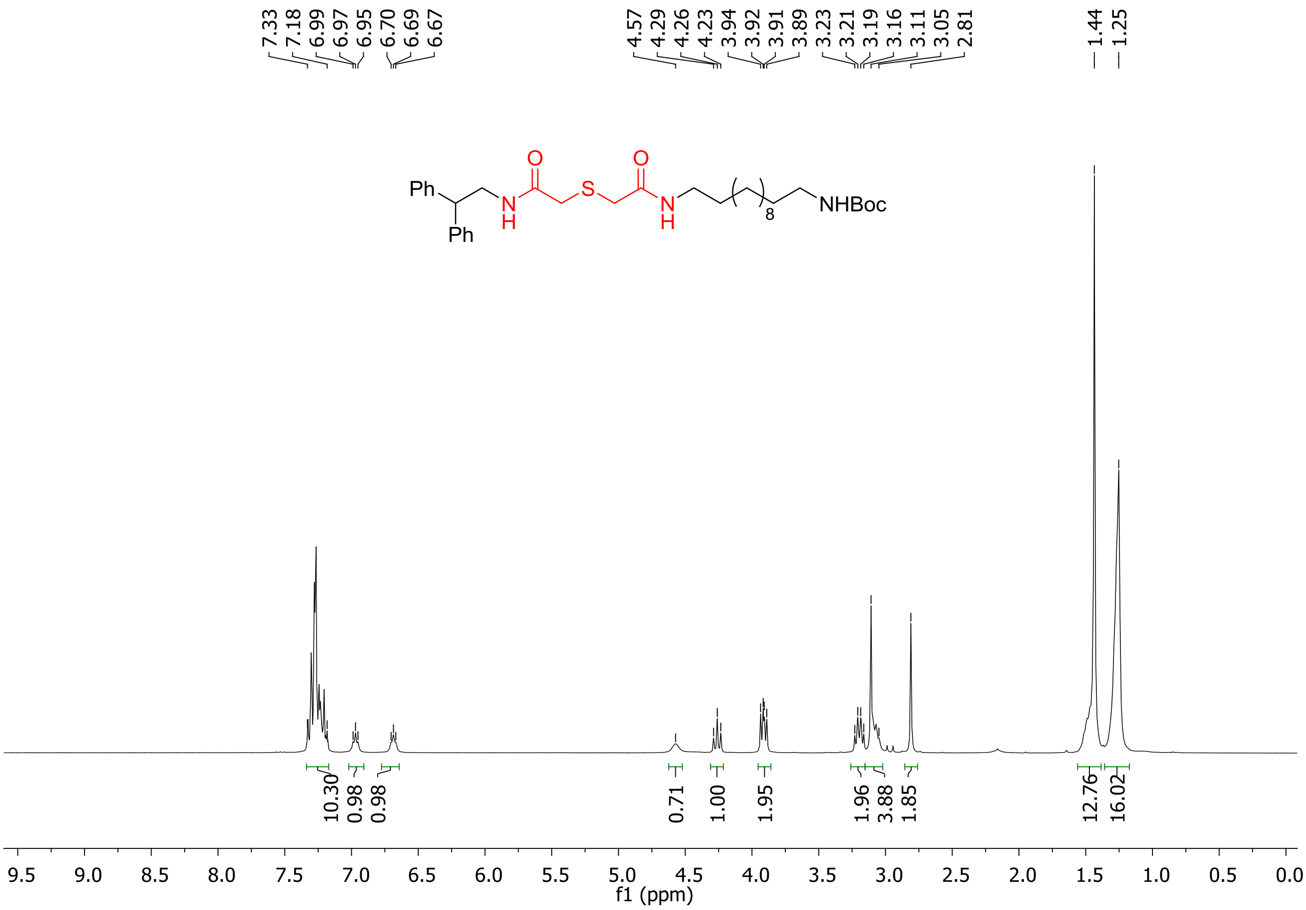

6b (^{13}C NMR, 75 MHz, $CDCl_3$, 298K)

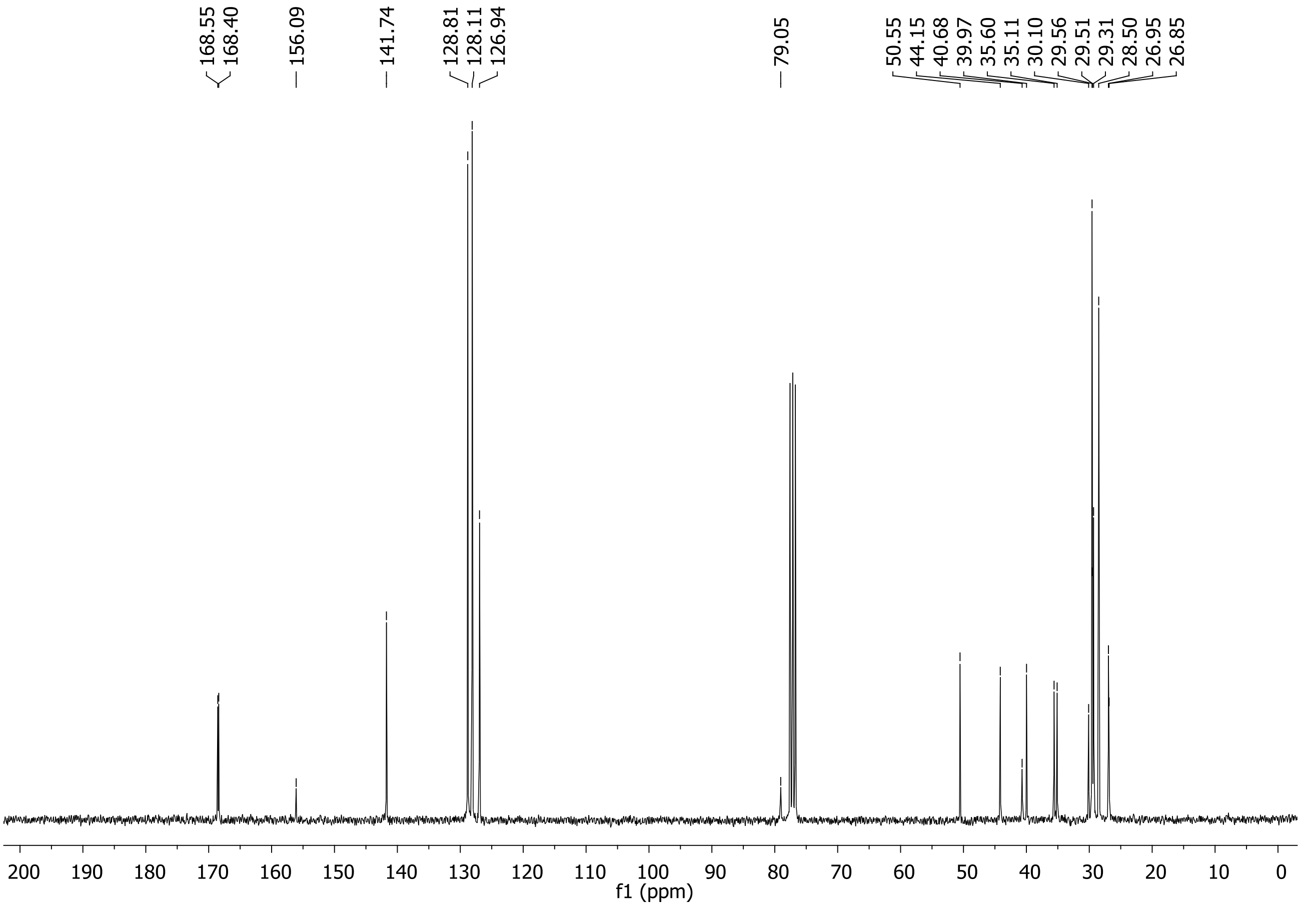

***E*-7a** (^{1}H NMR, 300 MHz, $CDCl_3$ + MeOD, 298K)

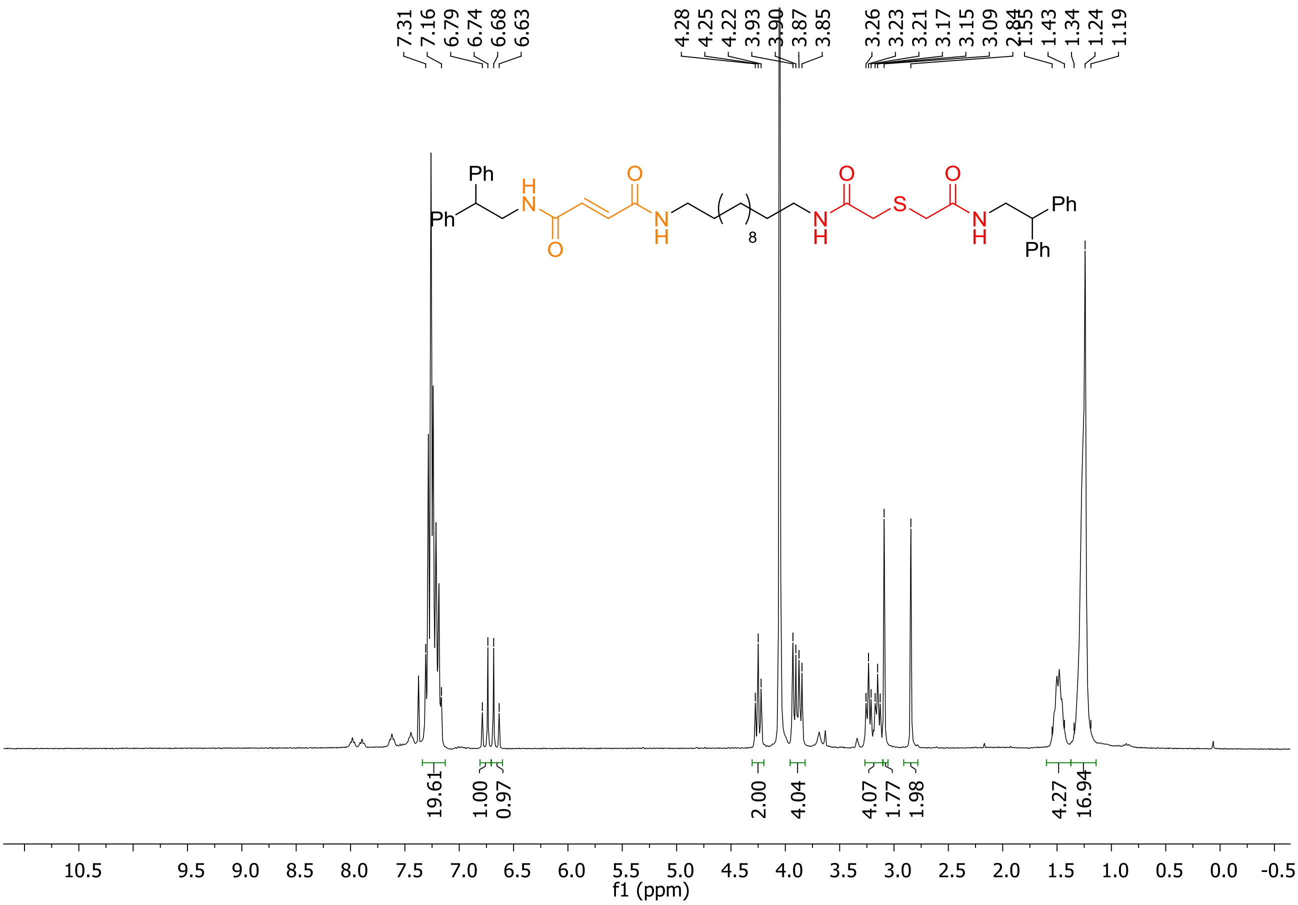

***E*-7a** (^{13}C NMR, 75 MHz, $CDCl_3$ + MeOD, 298K)

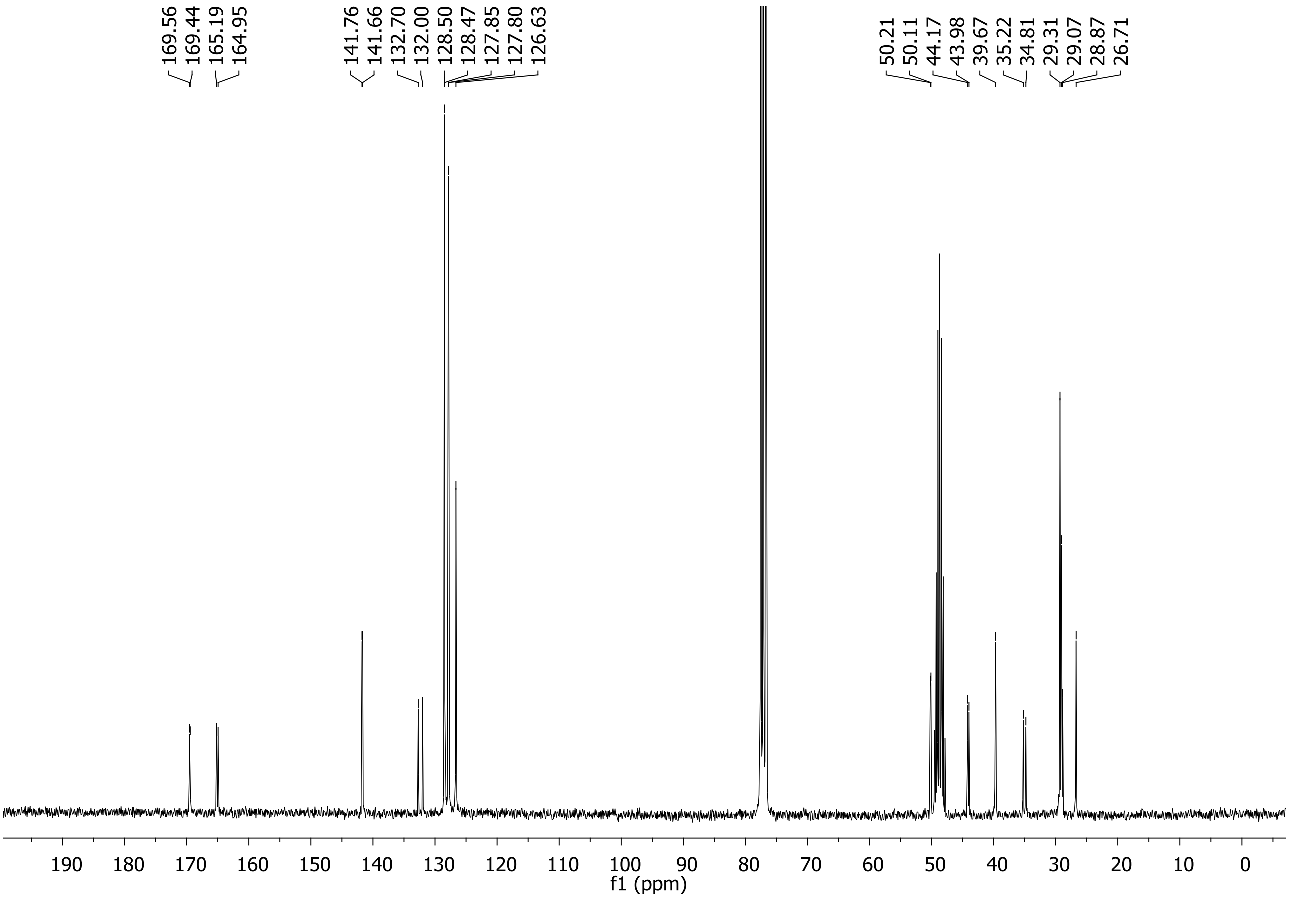

6b (^{1}H NMR, 300 MHz, $CDCl_3$, 298K)

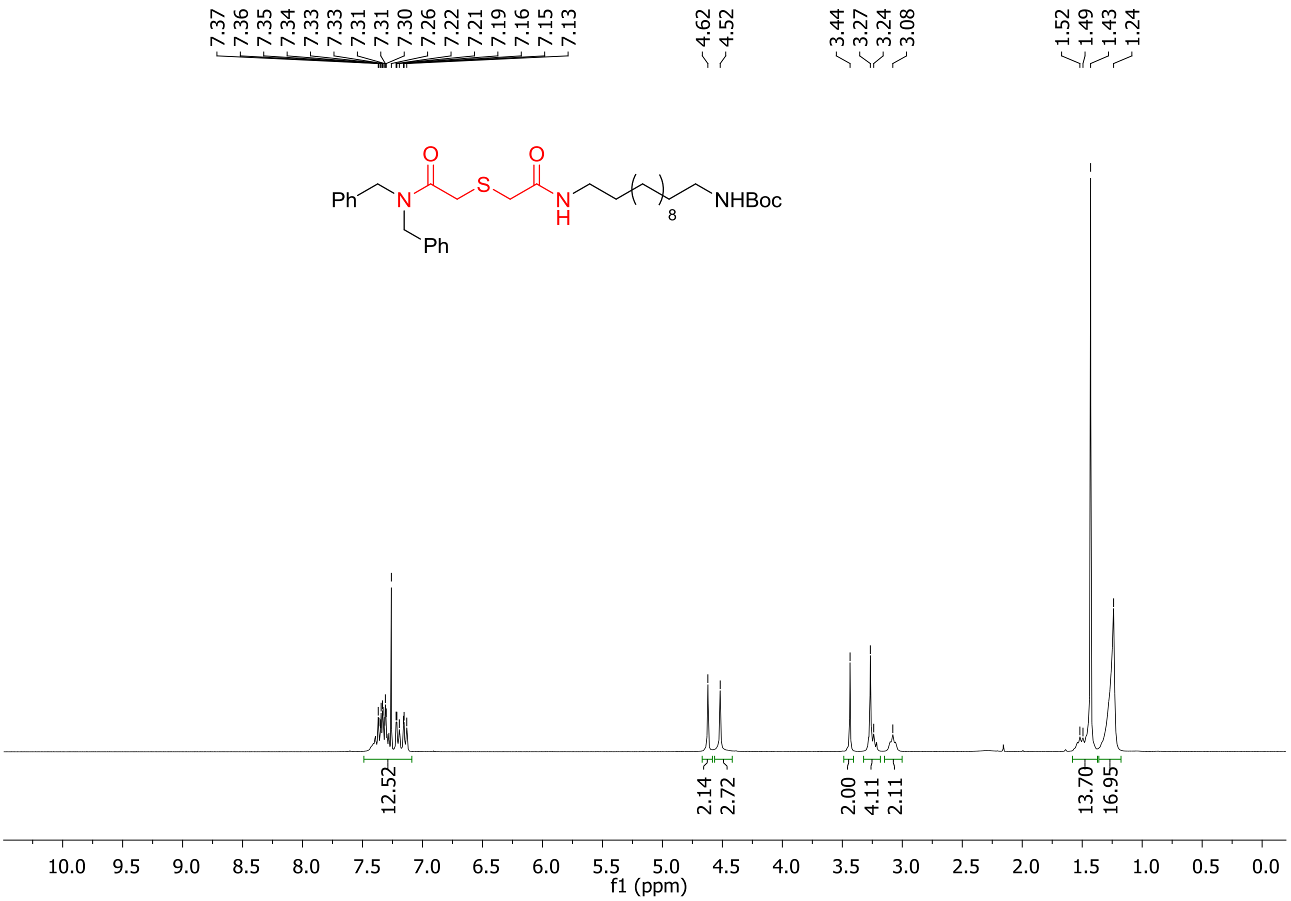

6b (^{13}C NMR, 75 MHz, $CDCl_3$, 298K)

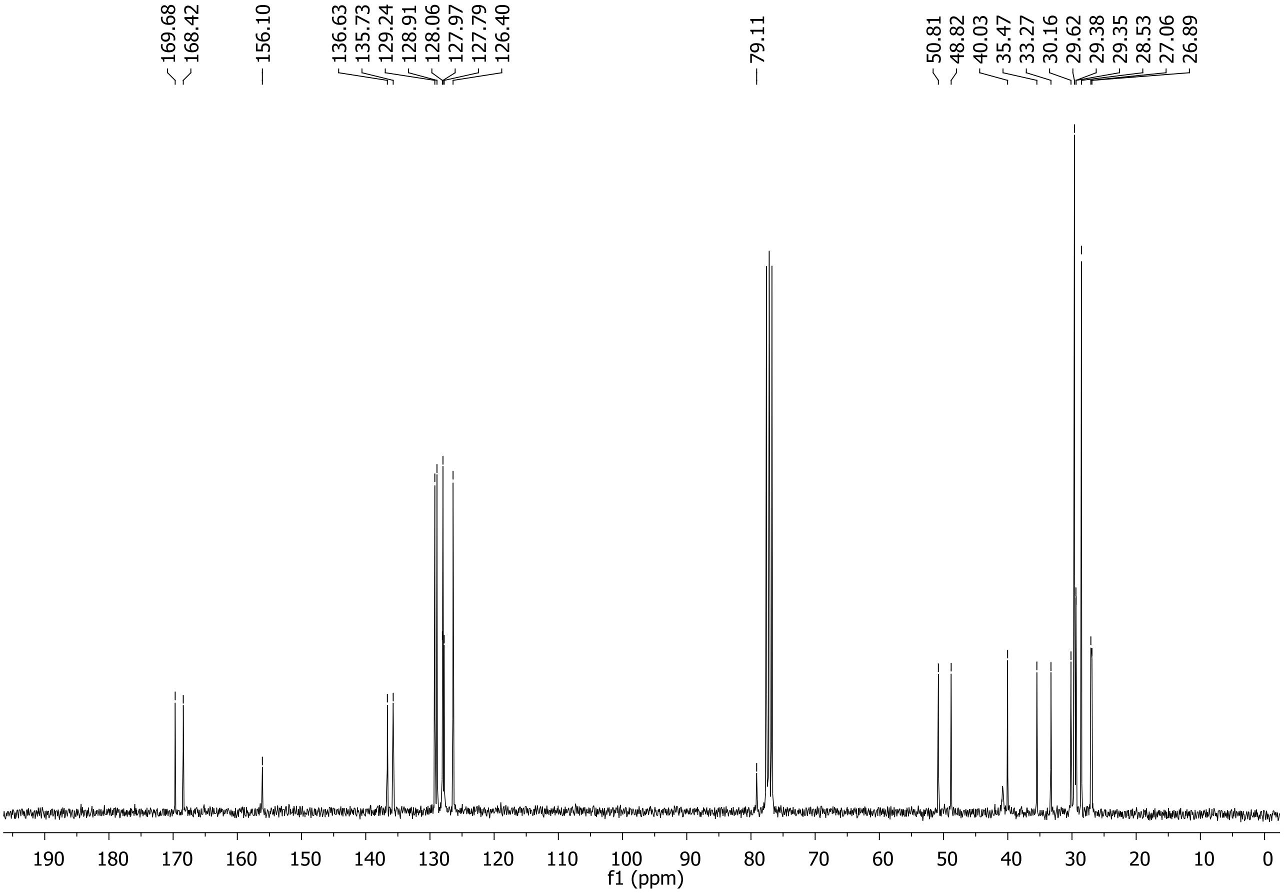

***E*-7b** (^{1}H NMR, 400 MHz, $CDCl_3$, 298K)

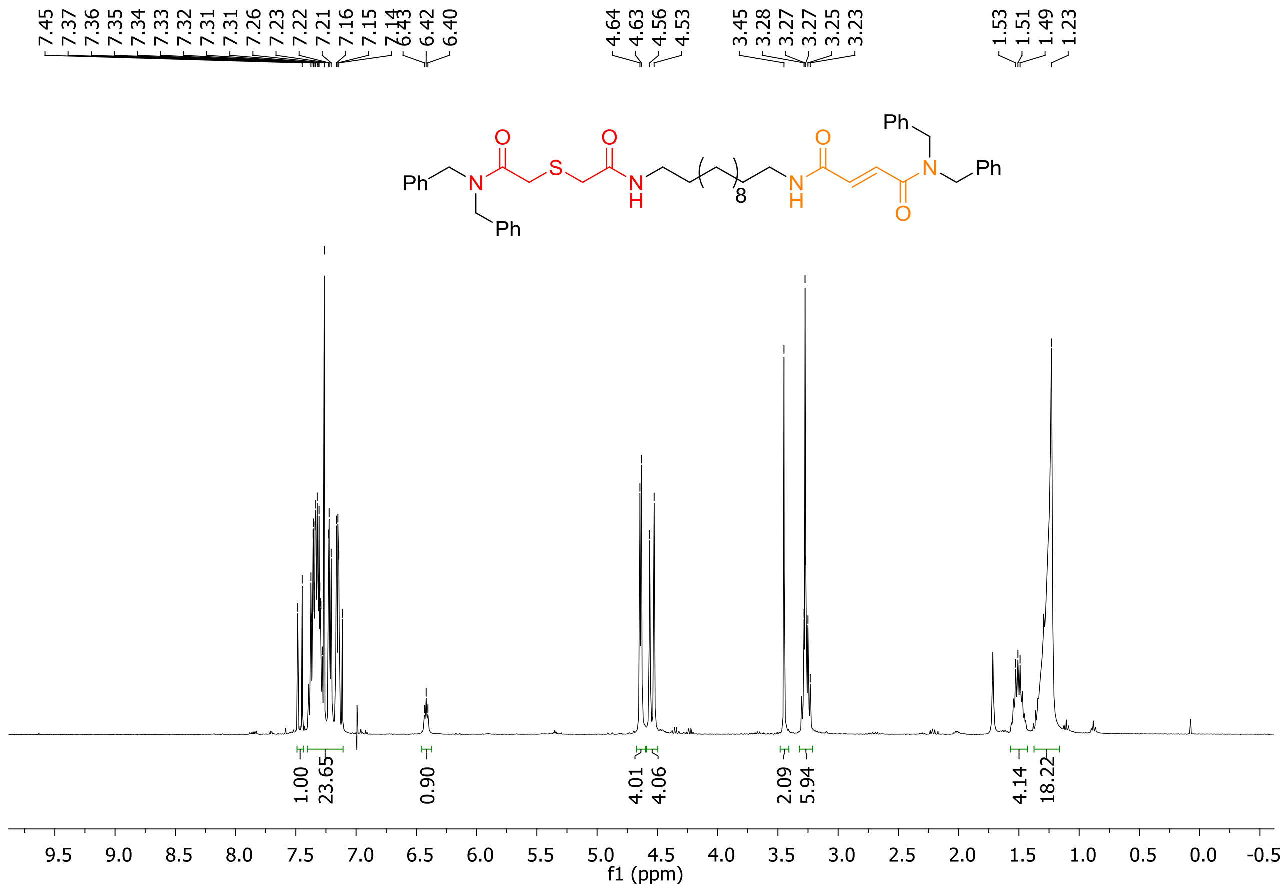

***E*-7b** (^{13}C NMR, 75 MHz, $CDCl_3$, 298K)

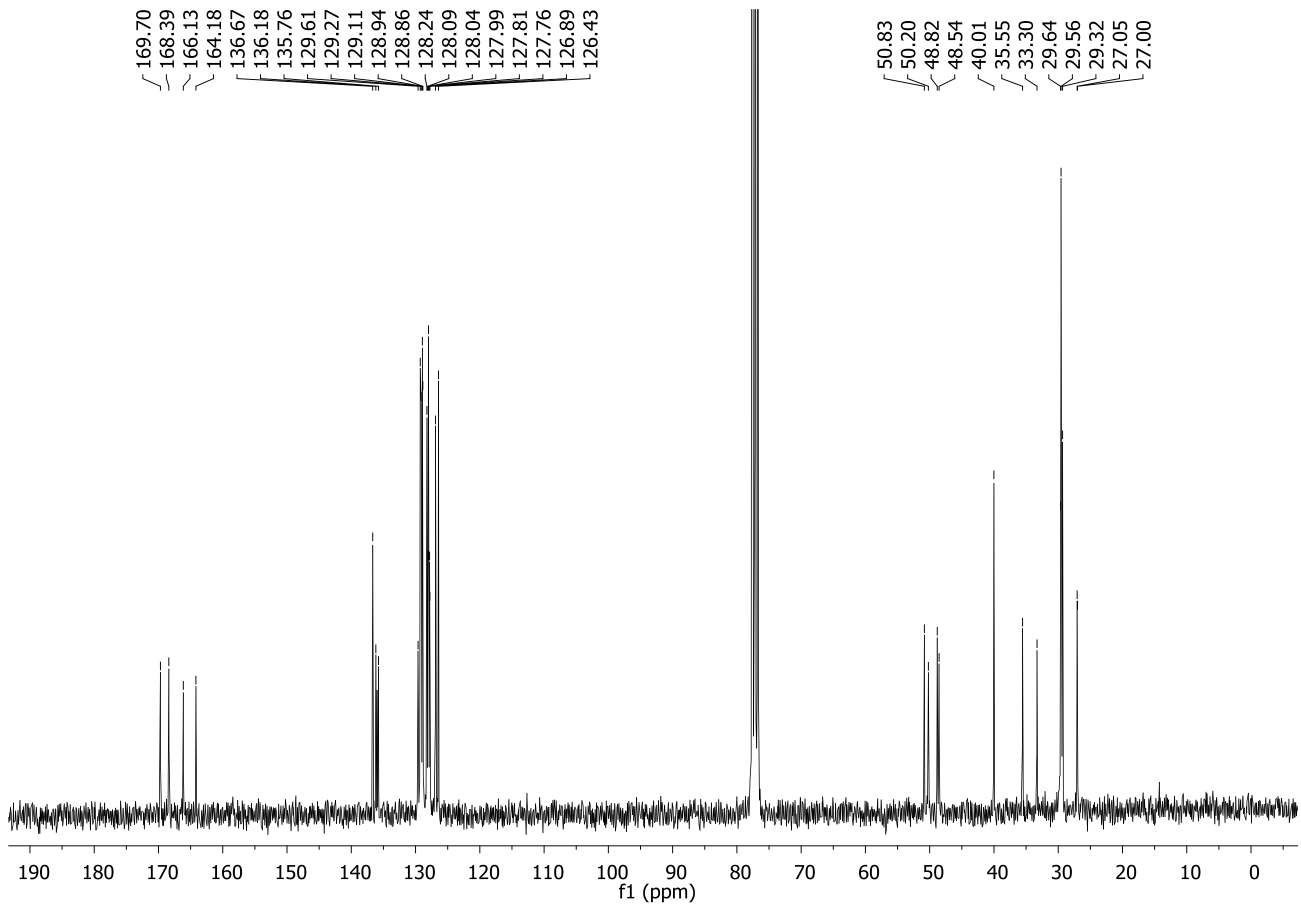

***Z*-7a** (^{1}H NMR, 300 MHz, $CDCl_3$, 298K)

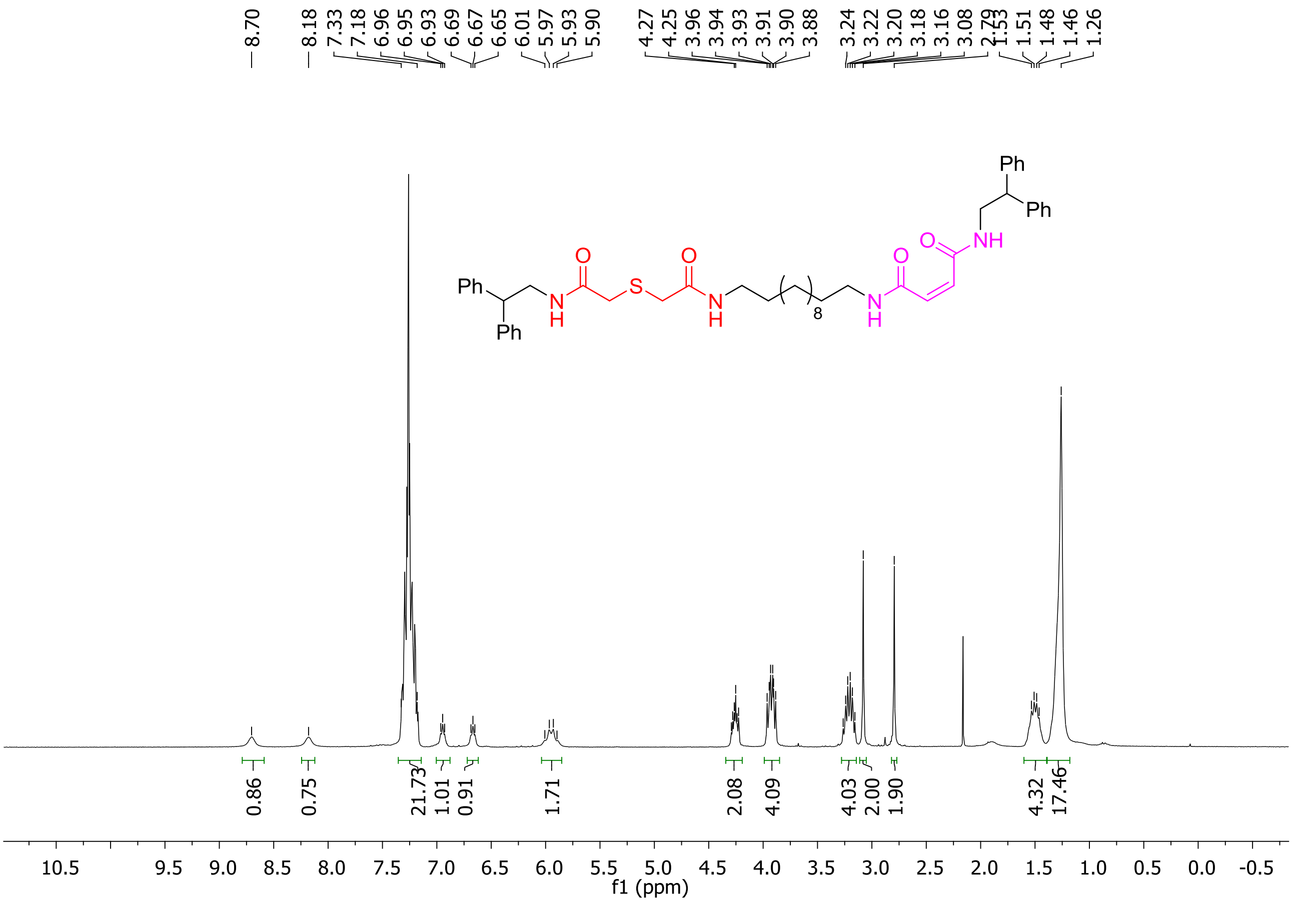

***Z*-7a** (^{13}C NMR, 75 MHz, $CDCl_3$, 298K)

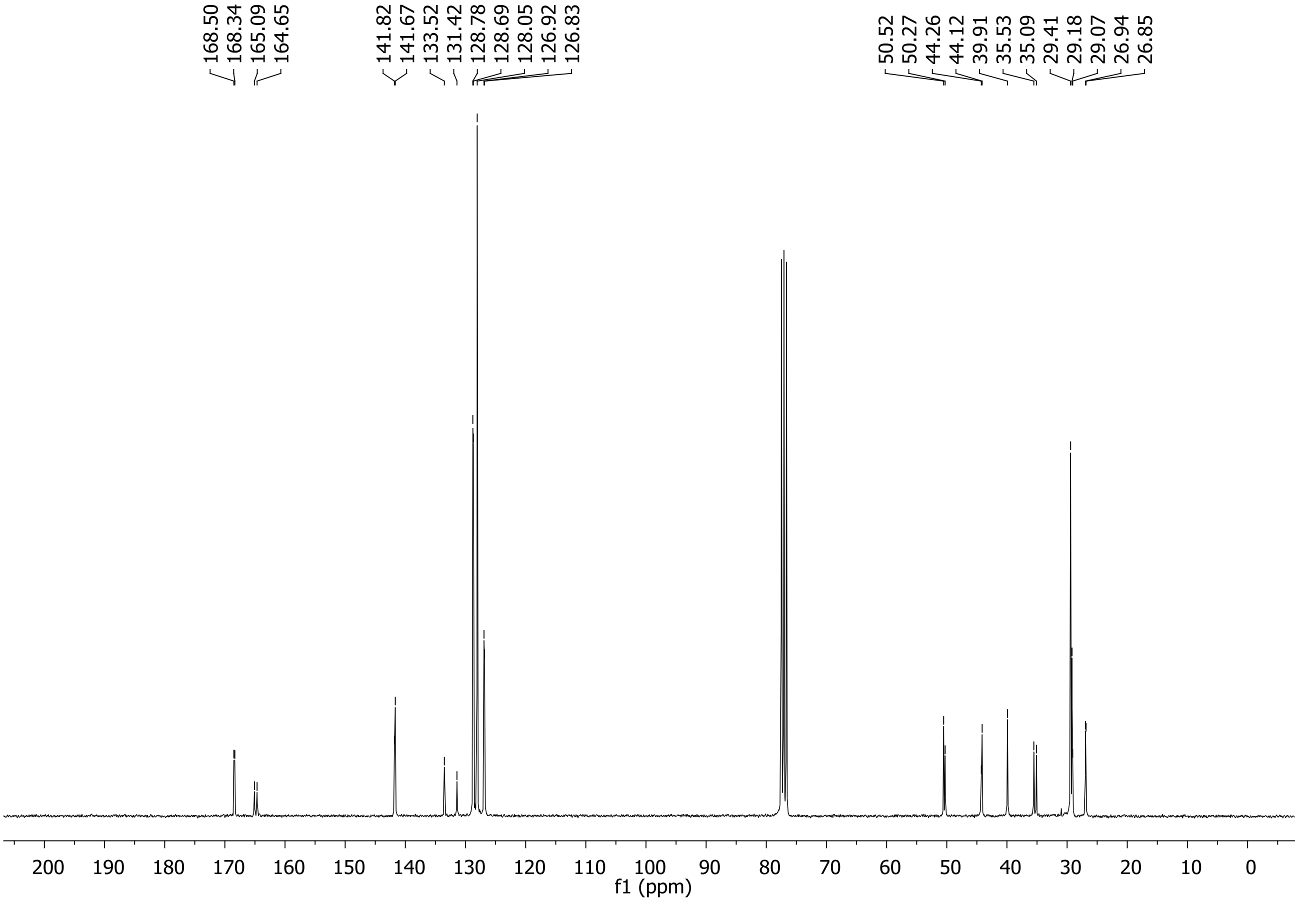

***Z*-7b** (^{1}H NMR, 400 MHz, $CDCl_3$, 298K)

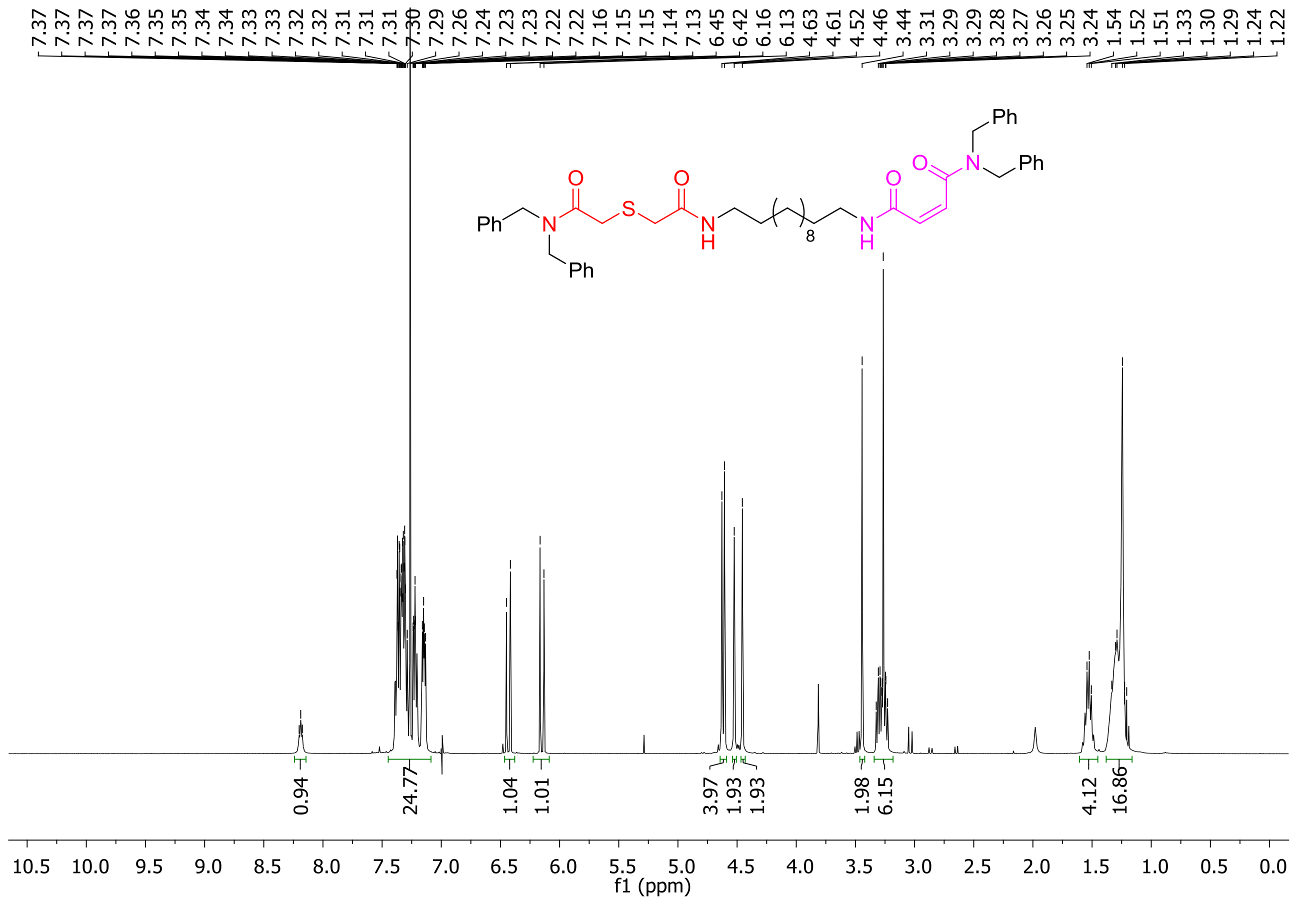

***Z*-7b** (^{13}C NMR, 100 MHz, $CDCl_3$, 298K)

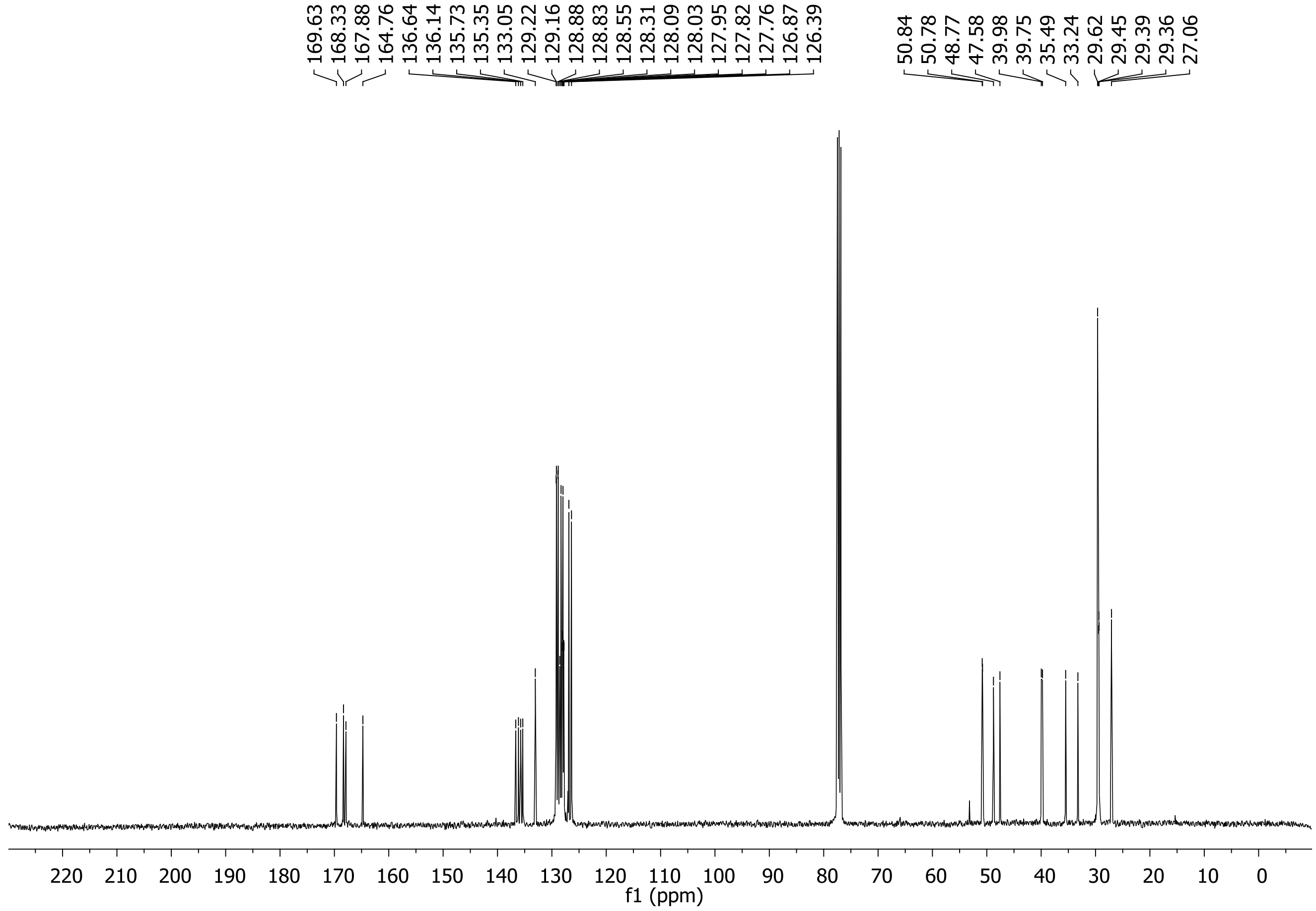

3a (^{1}H NMR, 300 MHz, $CDCl_3$, 298K)

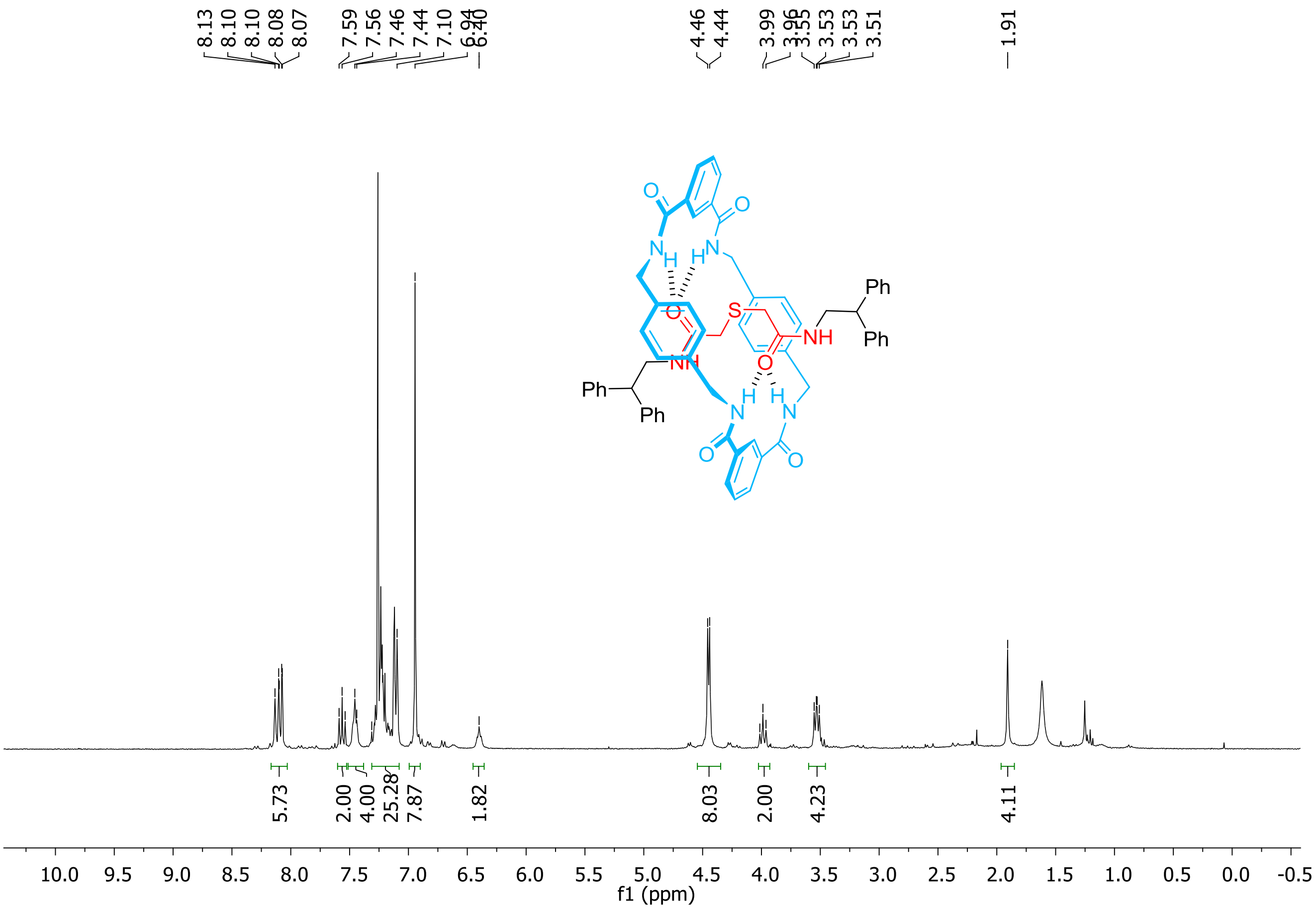

3a (^{13}C NMR, 100 MHz, $CDCl_3$, 298K)

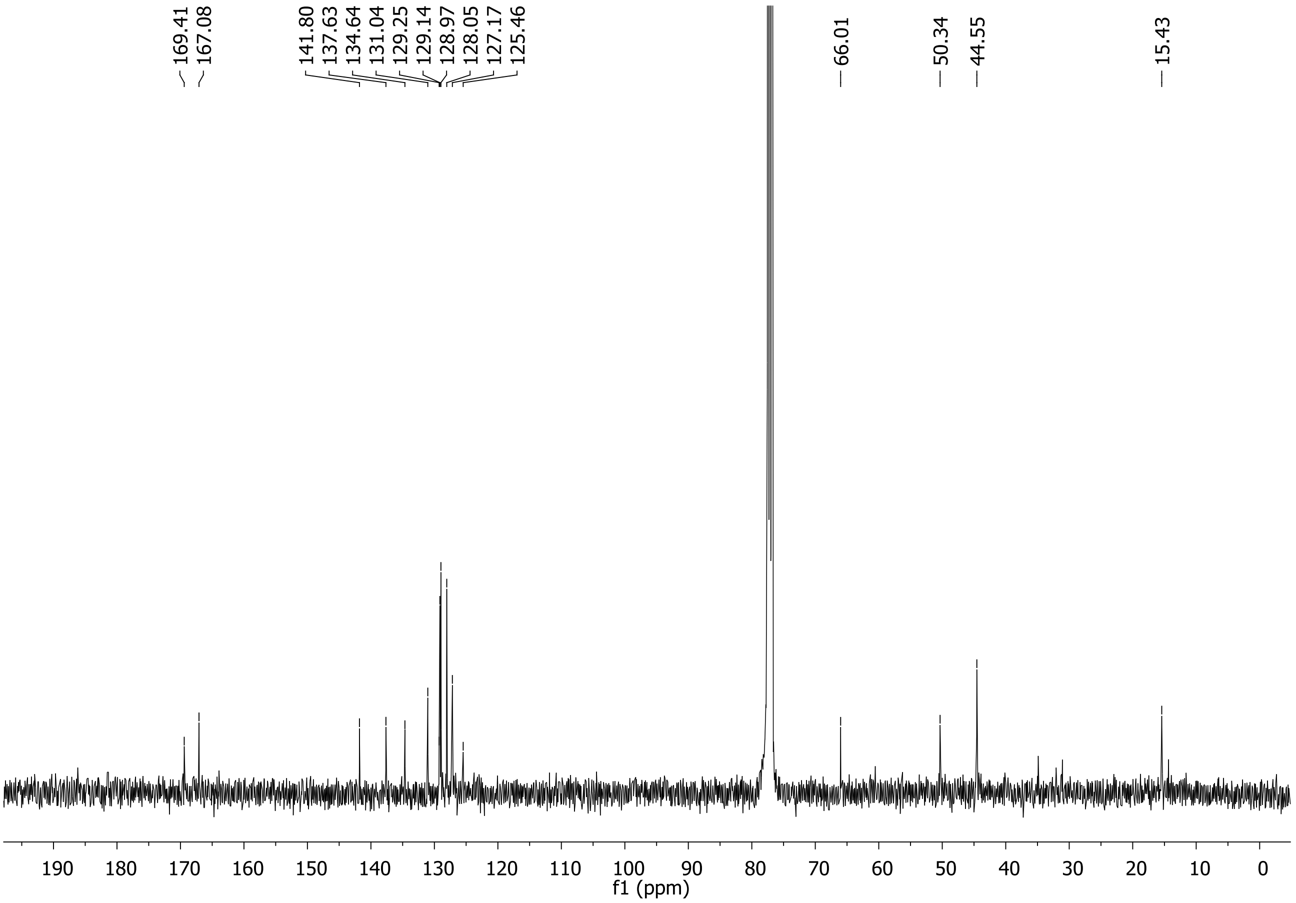

3b (^{1}H NMR, 400 MHz, $CDCl_3$, 298K)

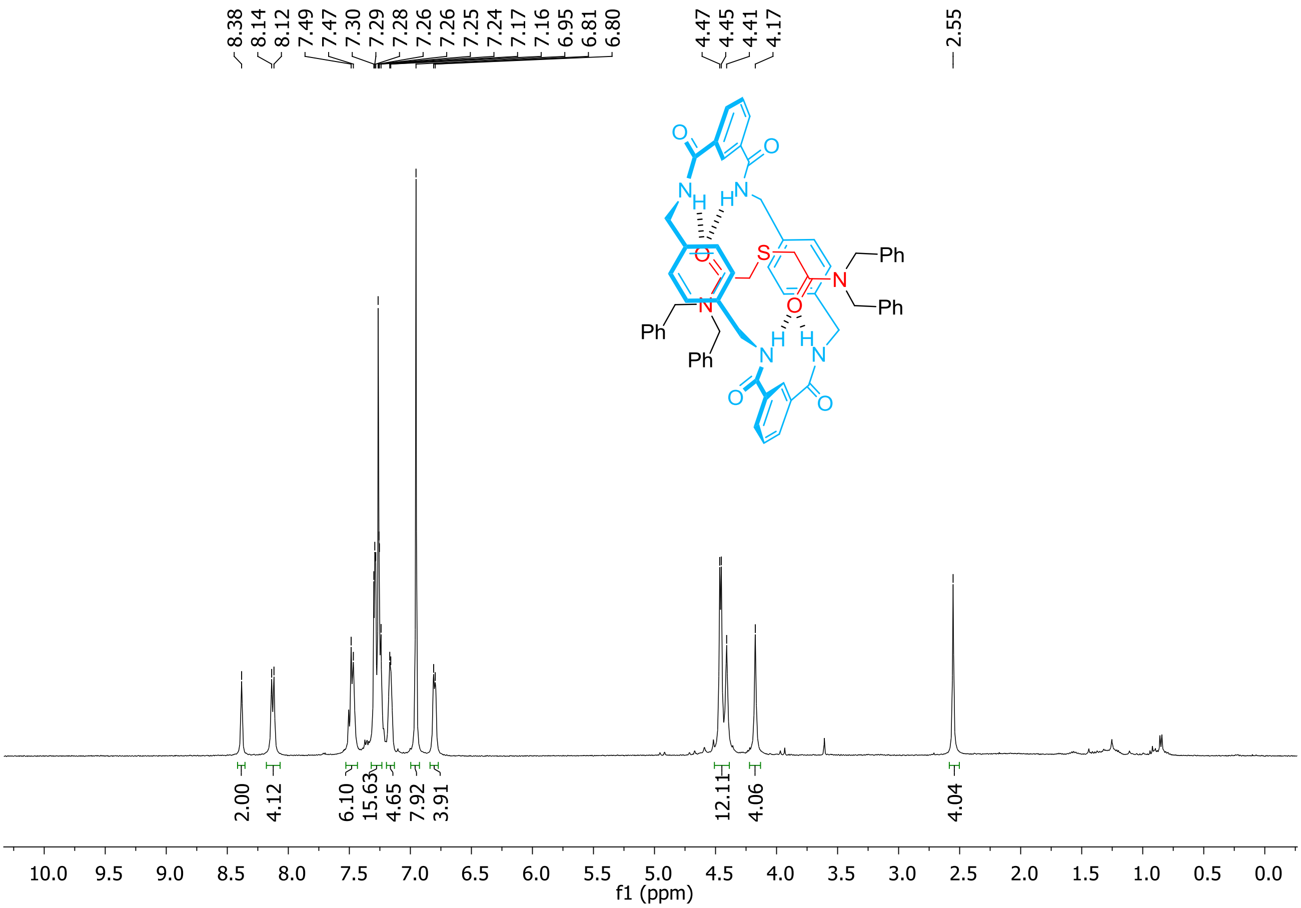

3b (^{13}C NMR, 4100 MHz, $CDCl_3$, 298K)

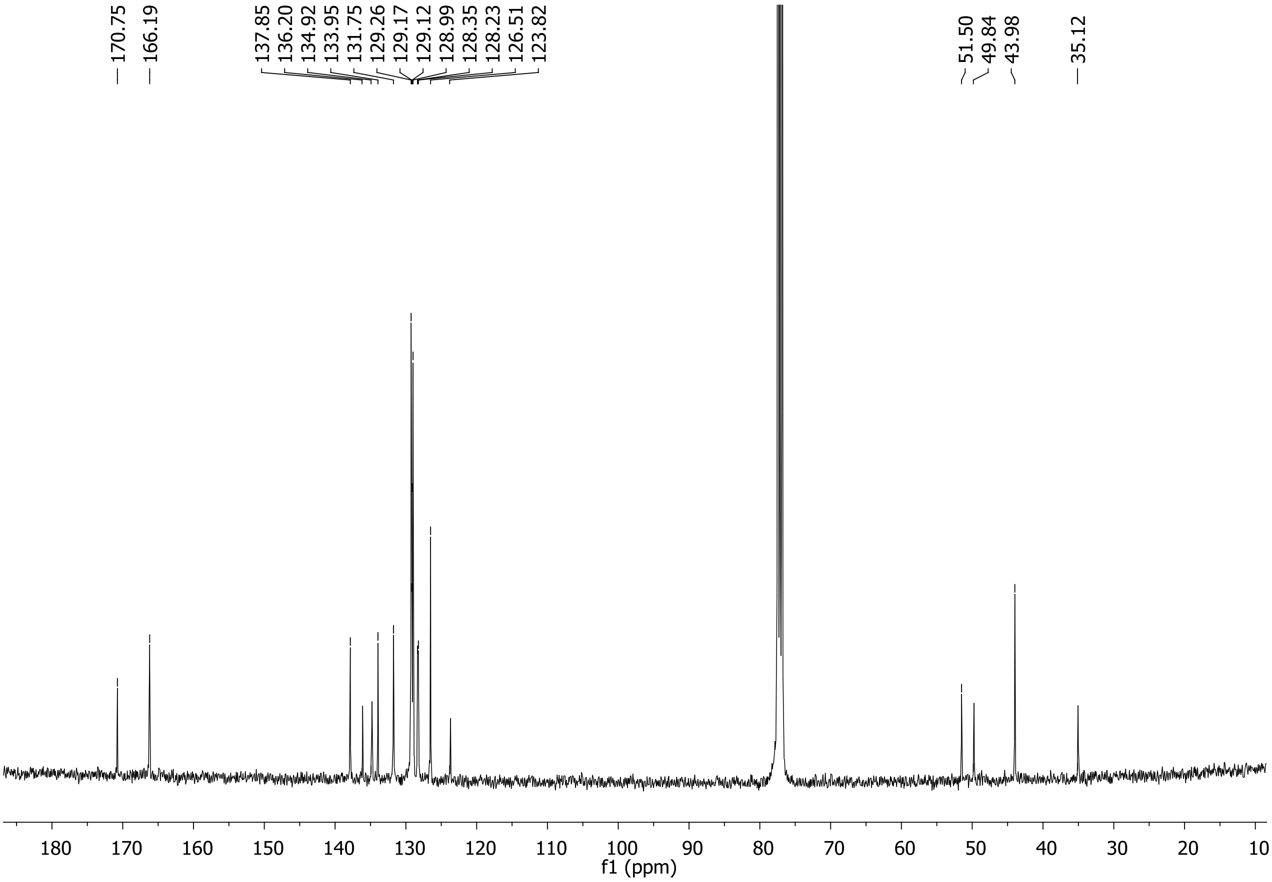

***E*-8a** (^{1}H NMR, 300 MHz, $CDCl_3$, 298K)

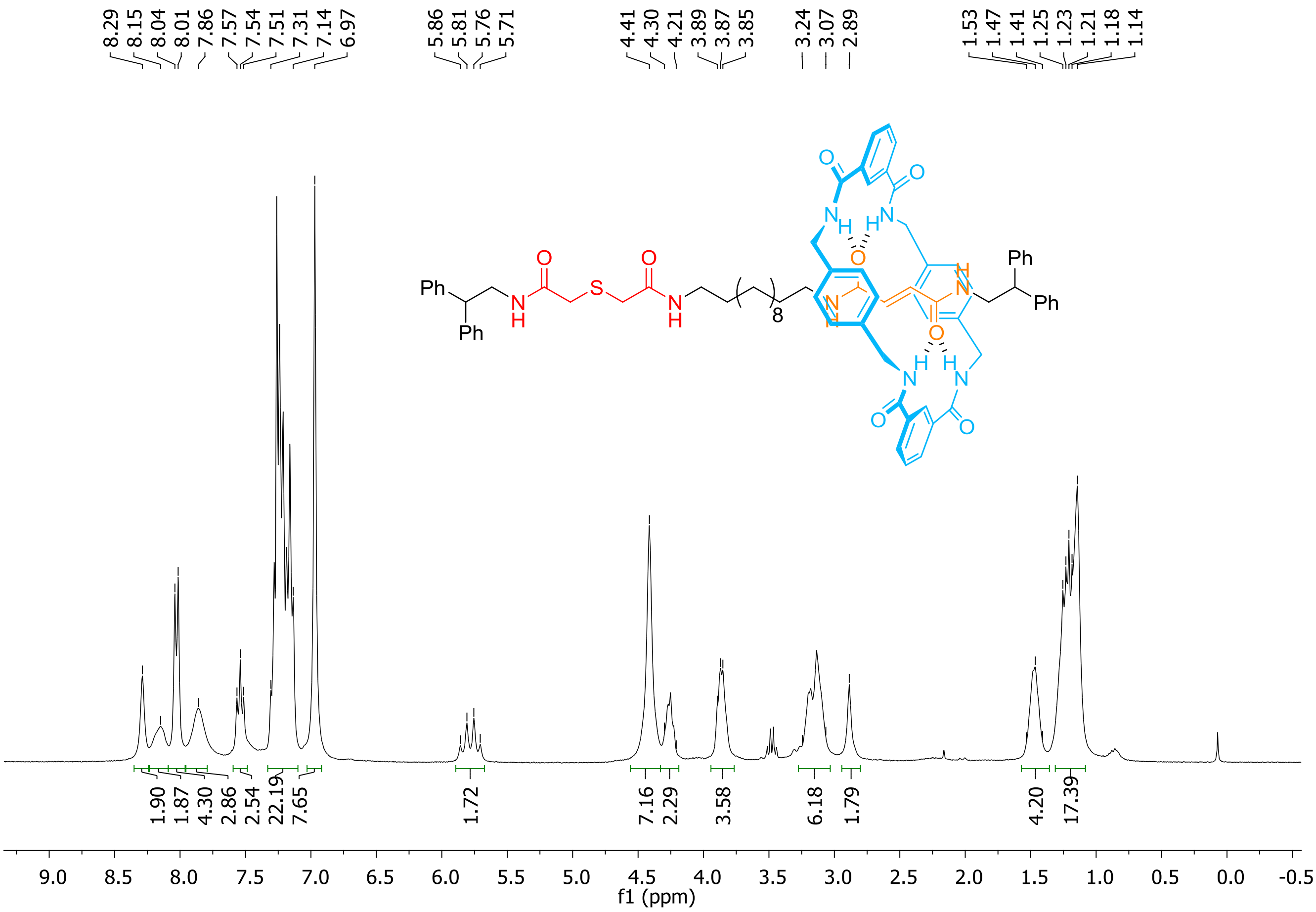

***E*-8a** (^{13}C NMR, 75 MHz, $CDCl_3$, 298K)

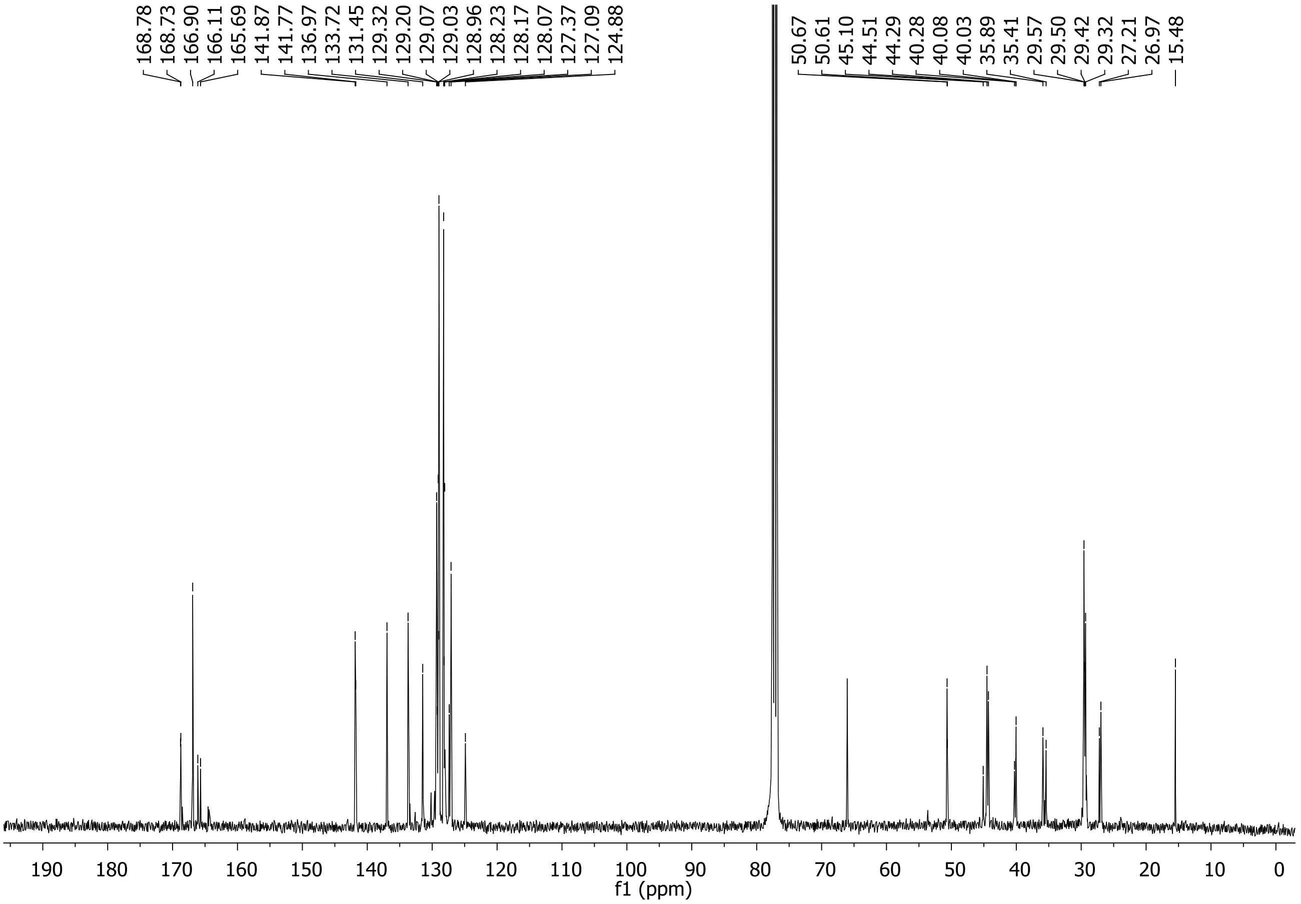

***E*-8b** (^{1}H NMR, 400 MHz, $CDCl_3$, 298K)

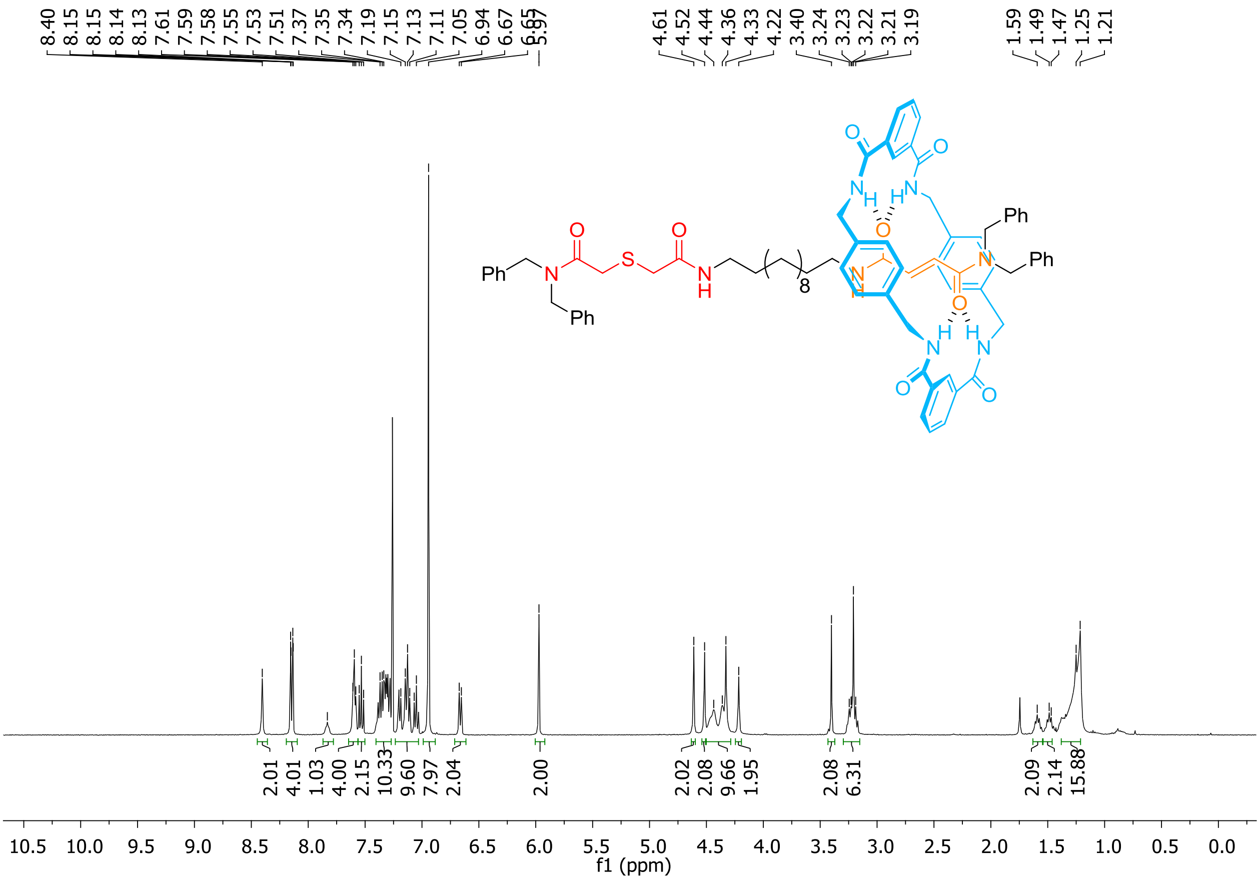

***E*-8b** (^{13}C NMR, 100 MHz, $CDCl_3$, 298K)

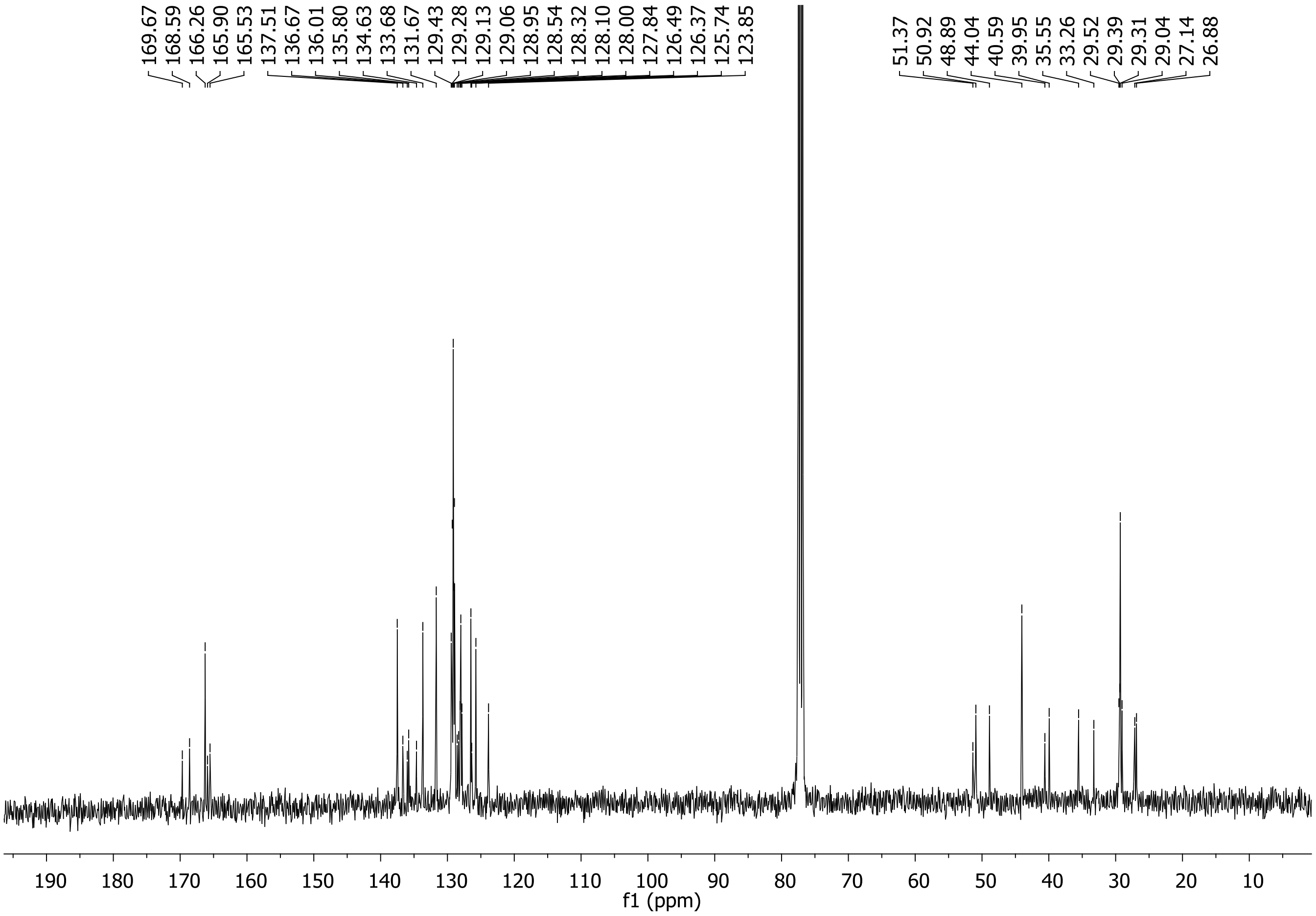

***Z*-8a** (^{1}H NMR, 400 MHz, $CDCl_3$, 298K)

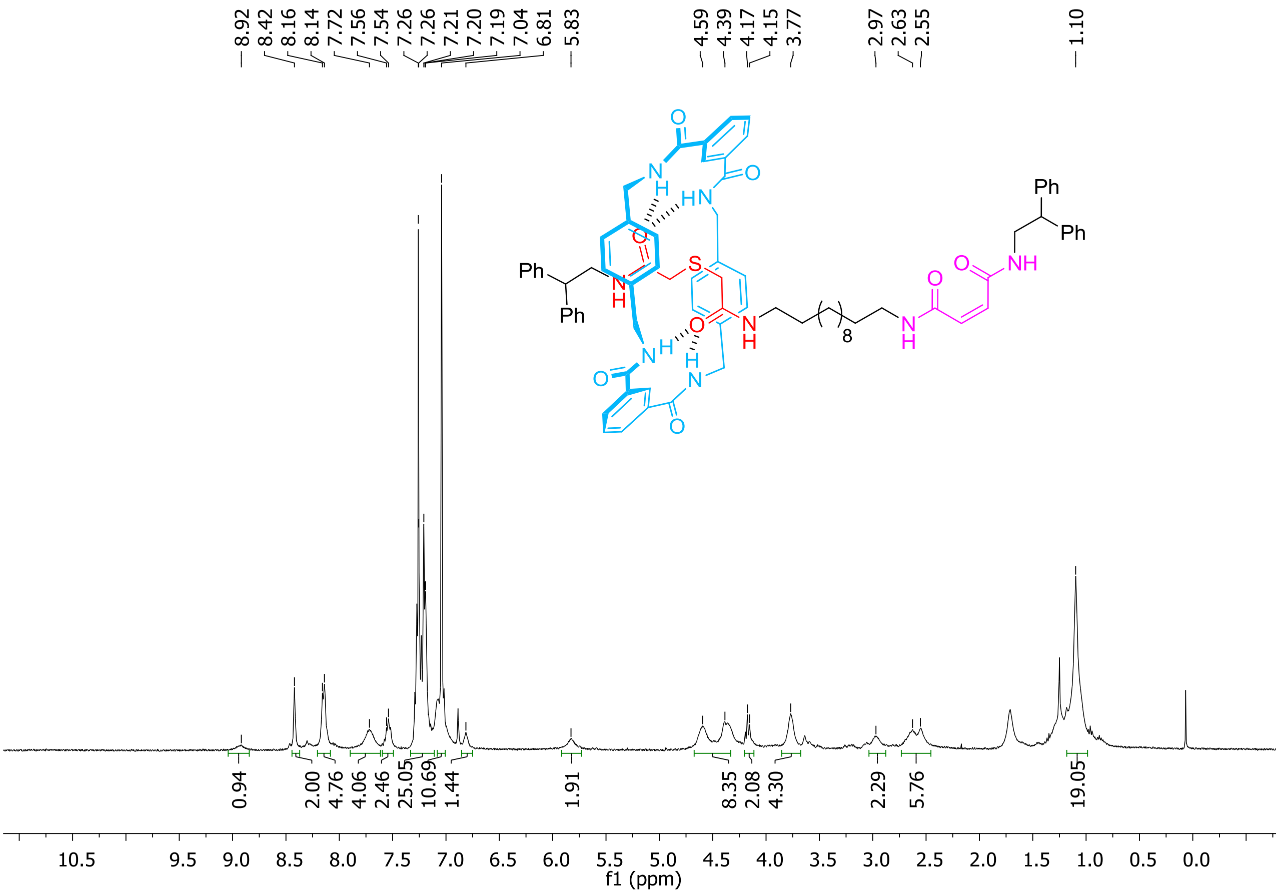

***Z*-8a** (^{13}C NMR, 100 MHz, $CDCl_3$, 298K)

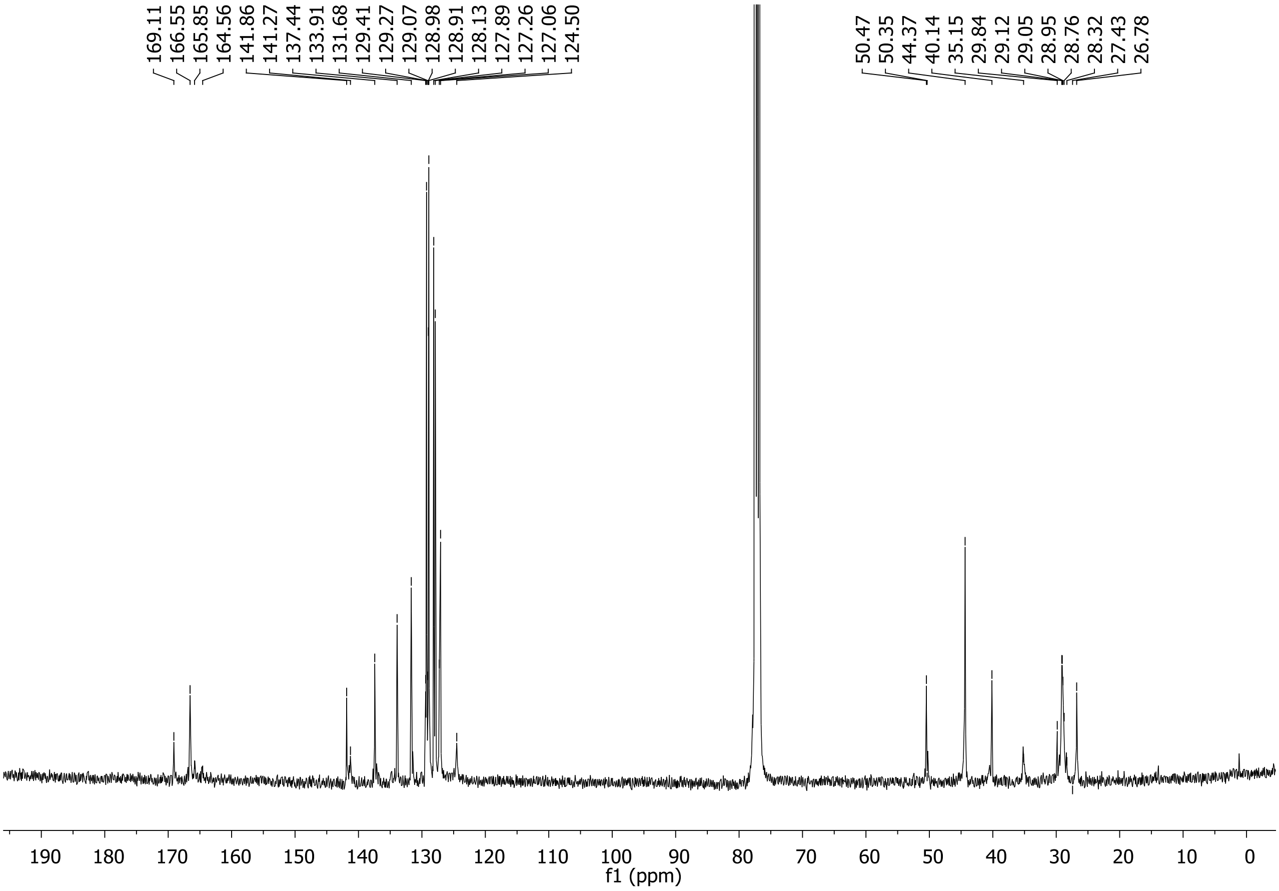

***Z*-8b** (^{1}H NMR, 400 MHz, $CDCl_3$, 298K)

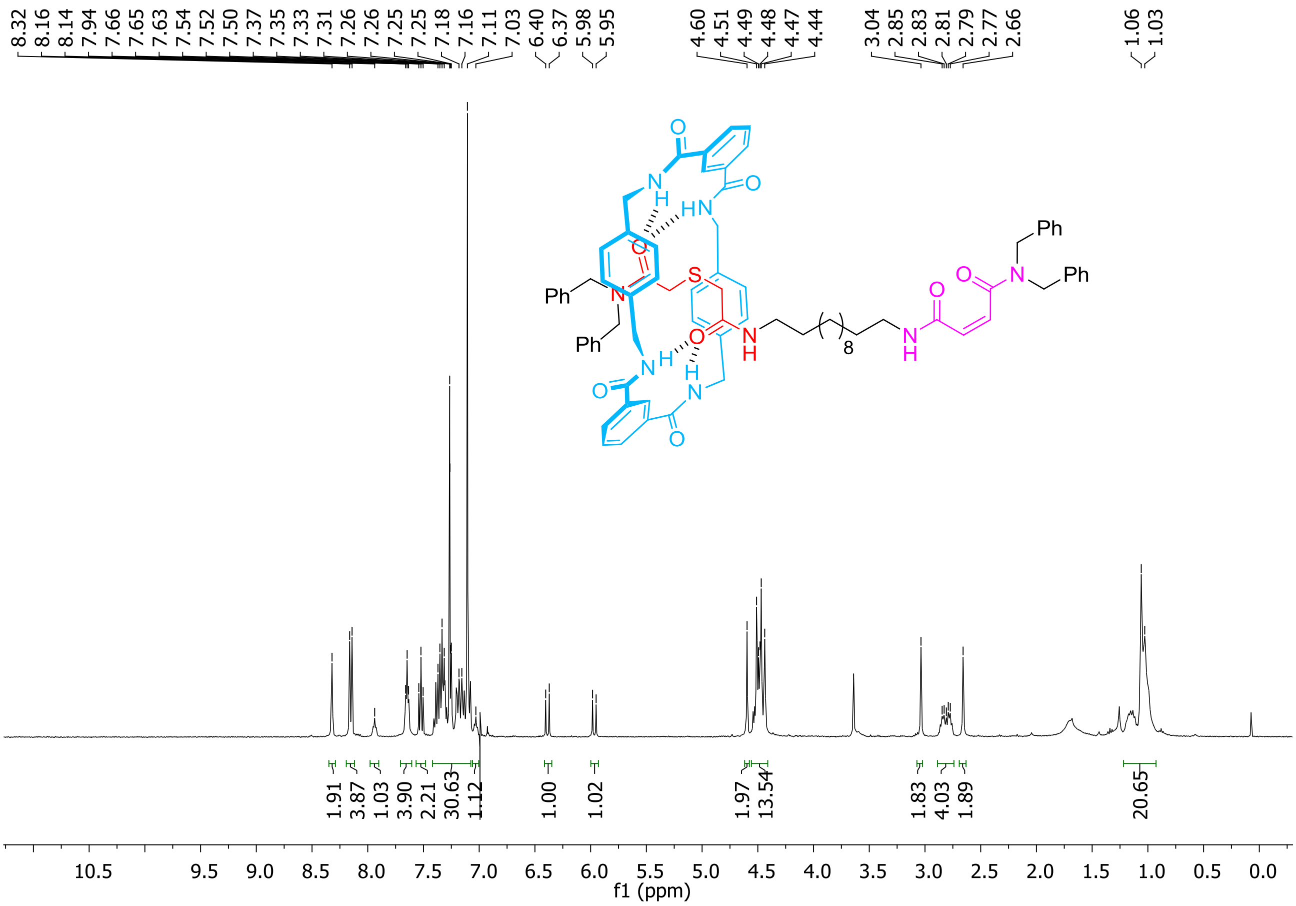

***Z*-8b** (^{13}C NMR, 100 MHz, $CDCl_3$, 298K)

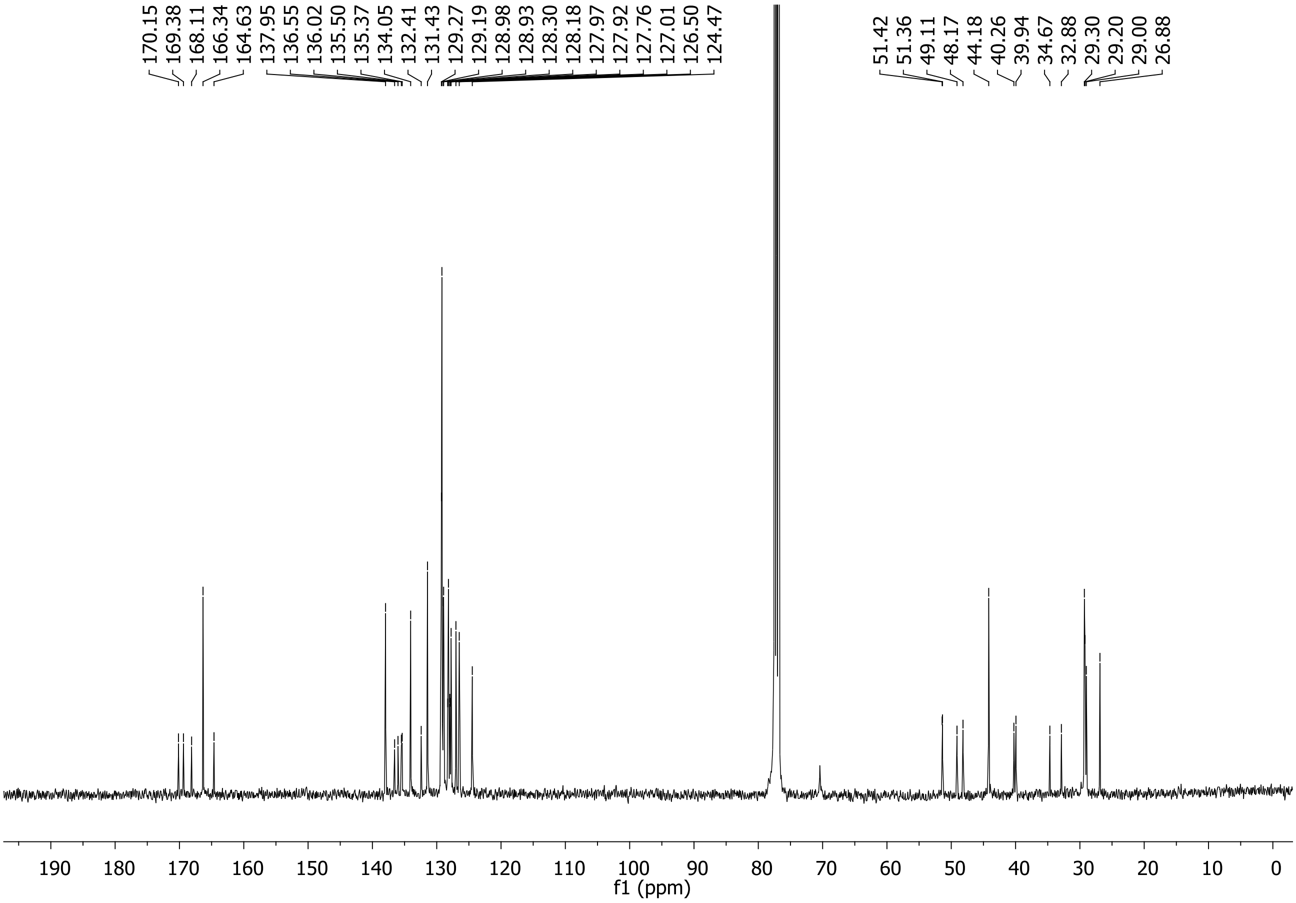

***E*-9** (^{1}H NMR, 300 MHz, $CDCl_3$, 298K)

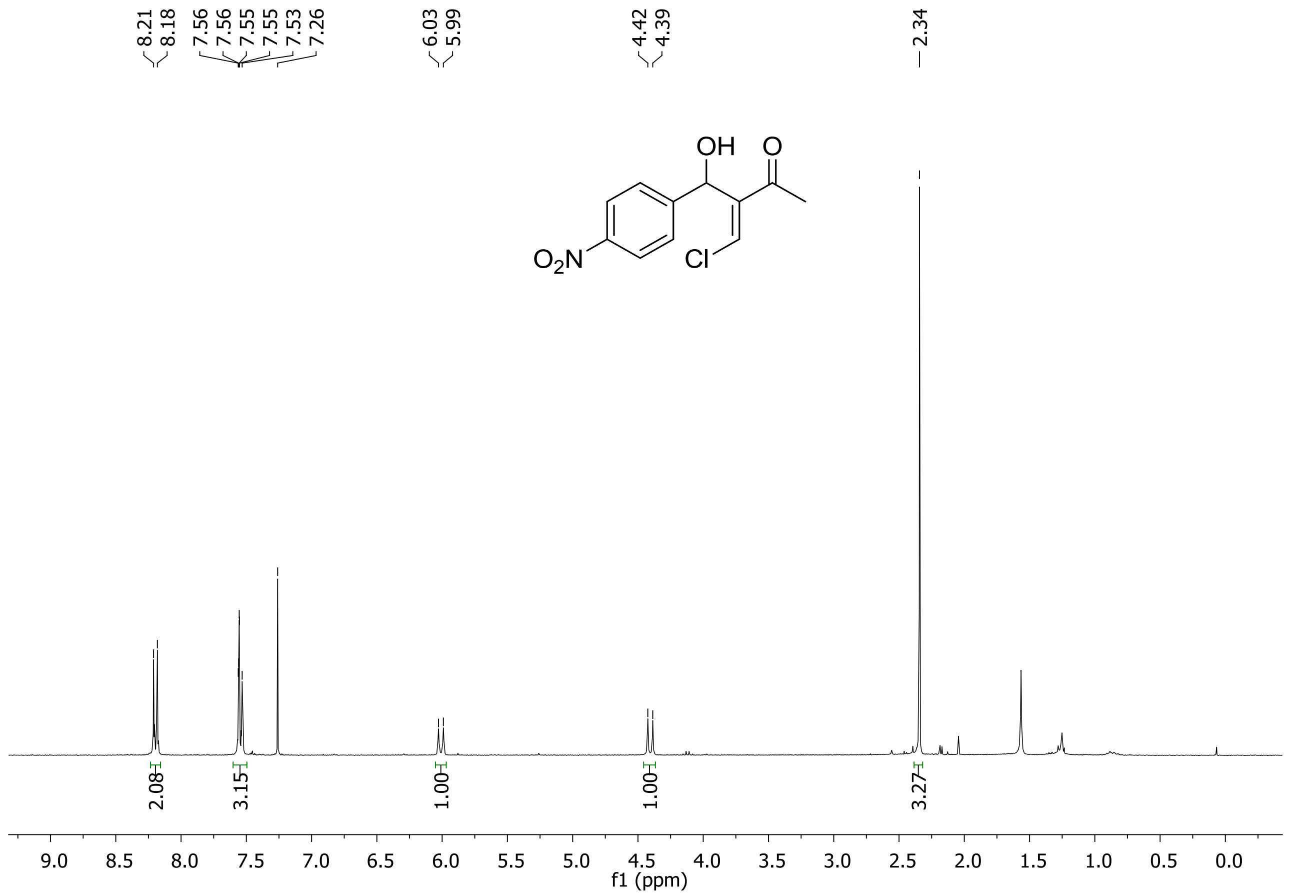

***Z*-9** (^{1}H NMR, 300 MHz, $CDCl_3$, 298K)

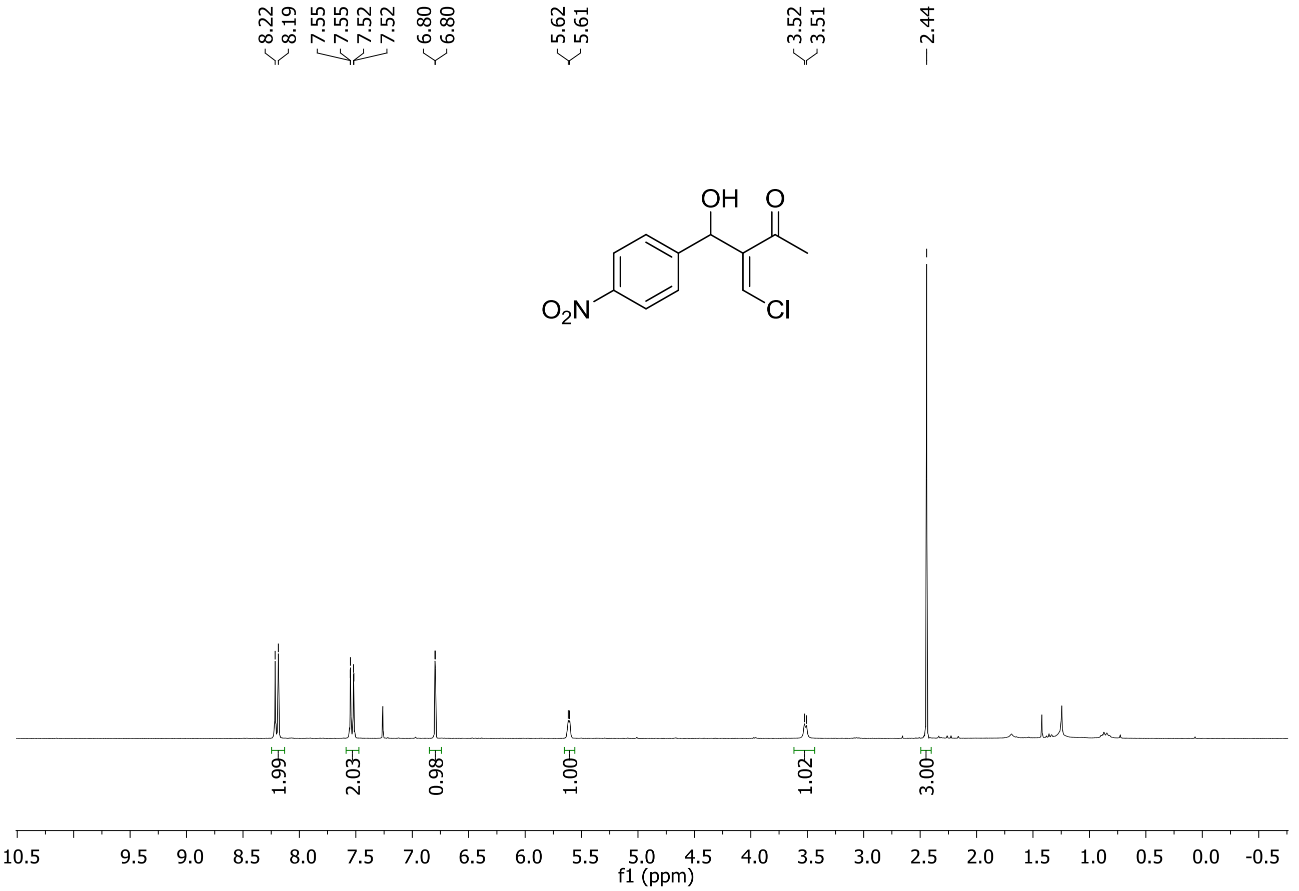

Supplement: Supplementary file 1 [file SC-008-C7SC00724H-s001.pdf]
